# Integrative network fusion-based multi-omics study for biomarker identification and patient classification of rheumatoid arthritis

**DOI:** 10.1186/s13020-023-00750-8

**Published:** 2023-05-04

**Authors:** Zihe Ding, Wenjia Chen, Hao Wu, Weijie Li, Xia Mao, Weiwei Su, Yanqiong Zhang, Na Lin

**Affiliations:** 1 Research Center of Traditional Chinese Medicine Theory and Literatures, Institute of Chinese Materia Medica, China Academy of Chinese Medical Sciences, No. 16, Nanxiaojie, Dongzhimennei, Beijing, 100700 China; 2 School of Life Sciences, Sun Yat-sen University, Guangzhou, 510275 China

**Keywords:** Rheumatoid arthritis, Patient classification, Biomarker identification, Precision medicine, Integration of the multi-omics data

## Abstract

**Background:**

Cold-dampness Syndrome (RA-Cold) and Hot-dampness Syndrome (RA-Hot) are two distinct groups of rheumatoid arthritis (RA) patients with different clinical symptoms based on traditional Chinese medicine (TCM) theories and clinical empirical knowledge. However, the biological basis of the two syndromes has not been fully elucidated, which may restrict the development of personalized medicine and drug discovery for RA diagnosis and therapy.

**Methods:**

An integrative strategy combining clinical transcriptomics, phenomics, and metabolomics data based on clinical cohorts and adjuvant-induced arthritis rat models was performed to identify novel candidate biomarkers and to investigate the biological basis of RA-Cold and RA-Hot.

**Results:**

The main clinical symptoms of RA-Cold patients are joint swelling, pain, and contracture, which may be associated with the dysregulation of T cell-mediated immunity, osteoblast differentiation, and subsequent disorders of steroid biosynthesis and phenylalanine metabolism. In contrast, the main clinical symptoms of RA-Hot patients are fever, irritability, and vertigo, which may be associated with various signals regulating angiogenesis, adrenocorticotropic hormone release, and NLRP3 inflammasome activation, leading to disorders of steroid biosynthesis, nicotinamide, and sphingolipid metabolism. IL17F, 5-HT, and IL4I1 were identified as candidate biomarkers of RA-Cold, while S1P and GLNS were identified as candidate biomarkers of RA-Hot.

**Conclusions:**

The current study presents the most comprehensive metabonomic and transcriptomic profiling of serum, urine, synovial fluid, and synovial tissue samples obtained from RA-Cold and RA-Hot patients and experimental animal models to date. Through the integration of multi-omics data and clinical independent validation, a list of novel candidate biomarkers of RA-Cold and RA-Hot syndromes were identified, that may be useful in improving RA diagnosis and therapy.

**Supplementary Information:**

The online version contains supplementary material available at 10.1186/s13020-023-00750-8.

## Introduction

Rheumatoid arthritis (RA) is a complex chronic autoimmune disease involving multiple organs with high morbidity and mortality, which seriously affects the quality of patients’ lives [1]. Growing clinical evidence shows that RA patients may be divided into various subtypes with different clinical symptoms, signs and often receiving different therapeutic strategies [2]. Due to the complexity of RA and the overlapping of multiple symptoms, precise clinical diagnosis and treatment are challenging [3]. Traditional Chinese medicine (TCM) syndromes are distinctive symptom groups summarized based on TCM theories and clinical empirical knowledge for thousands of years. TCM holds syndromes as the core diagnostic criteria and therapeutic guidance [4]. Notably, a total of 196 TCM syndromes has been included in the *International Classification of Diseases, 11th Revision* (ICD-11, the latest version 2021-05), implying that syndrome-based diagnosis in TCM has been recognized by modern healthcare systems [5, 6]. Cold-dampness Syndrome (also known as Cold Pattern in ICD-11 with a code TM1-SE73) and Hot-dampness Syndrome (also known as Hot Pattern in ICD-11 with a TM1-SE72) are the two most common TCM syndromes of RA, accounting for 19.82% and 43.86% in clinics, respectively [7]. Especially, the main clinical manifestation of RA patients with Cold-dampness Syndrome is severe arthralgia induced by cold stimulations, often treated by the TCM prescriptions with the efficacy of warming meridians, dispelling cold and dredging collaterals, such as Wutou Decoction (WTD) [8, 9]. In contrast, RA patients with hot-dampness syndrome show local burning, redness and swelling, severe pain in the joints, better treated by heat clearing and dehumidification prescriptions, such as Baihu Guizhi Decoction (BHGZD) [10, 11]. Despite many years of research, no biomarkers for differentiate RA patients with cold and hot-dampness syndromes are available to use in clinical practice.

Recent advances in high-throughput technologies have provided new opportunities to understand the pathophysiology of RA. The integration of the multi-omics data means that clinical phenotypes (phenomics), proteins (proteomics), genes (genomics), RNAs (transcriptomics) and metabolites (metabonomics) can be analyzed simultaneously and illustrated by interaction networks with links at various molecular levels [12–14]. Accumulating studies have indicated that the integrated analysis of multi-omics data may provide useful insight into RA pathogenesis, identification of therapeutic targets and biomarker discovery. For example, Luan et al. [15] identified diagnostic biomarkers for seronegative and seropositive RA patients by integrated analysis of serum metabonomics and lipidomics. Souto-Carneiro et al. [16] identified novel biomarkers for seronegative rheumatoid arthritis and psoriatic arthritis by performing ^1^H nuclear magnetic resonance (NMR)-based metabolomic and lipidomic analyses of serum samples from a large cohort of patients in both categories, followed by a validation cohort analysis to confirm the biomarker as a diagnostic multivariable model for the two pathologies.

**Fig. 1:**
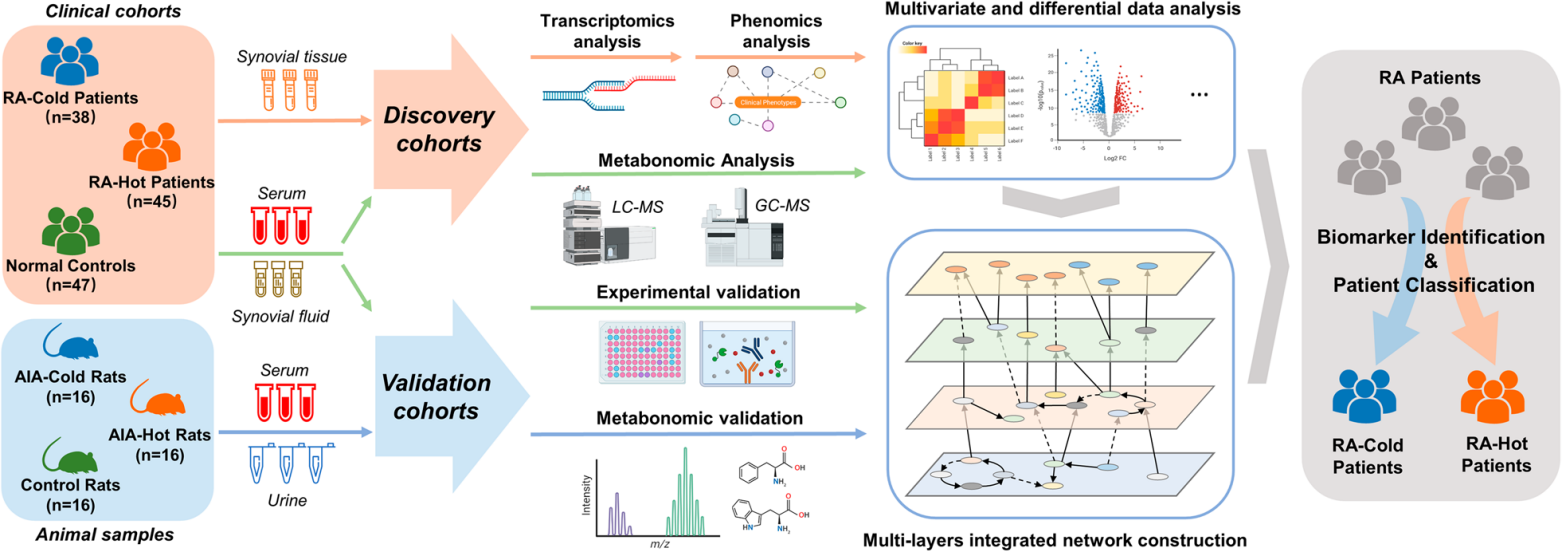
The flowchart of the integrative network fusion-based multi-omics strategy for our current study

In the current study, the phenomics, transcriptomics and metabonomics data were integrated and analyzed for elucidating the scientific connotation and identifying biomarkers of RA with cold and hot-dampness syndromes. We firstly collected multiple types of biological samples from clinical patients and experimental animal models to cover systemic and local specific pathological features. Multi-omics data mining based on biological network fusion was then proposed to expound the differences comprehensively and systematically between the two types of RA patient cohorts, and to further identify the relevant syndrome biomarkers (Fig. 1).

## Materials and methods

### Clinical sample collection

The study was approved by the medical ethics committee of the Third Affiliated Hospital of Beijing University of Chinese Medicine (BZYSY-2021KYKT8L-02). Written informed consents were obtained from all study subjects. Serum, synovial fluid, and synovial tissue samples were collected from RA patients between October 2017 and January 2022 in 10 hospitals, including Wang Jing Hospital of Chinese Academy of traditional Chinese Medicine (CACMS) (Beijing, China), Xi’an Red Cross Hospital (Xi’an, China), Peking University Shougang Hospital (Beijing, China), Baotou Mongolian TCM Hospital (Baotou, China), Henan Rheumatism Hospital (Zhengzhou, China), General Hospital of Southern Theatre Command (Guangzhou, China), Guang’anmen Hospital of CACMS, (Beijing, China), First Teaching Hospital of Tianjin University of TCM (Tianjin, China), and Gansu Provincial TCM Hospital (Lanzhou, China), and from normal control subjects in Beijing Sports Hospital (Beijing, China), Wang Jing Hospital of CACMS (Beijing, China) and First Teaching Hospital of Tianjin University of TCM (Tianjin, China). Patients were clinically diagnosed with Cold-dampness Syndrome of RA (RA-Cold) and Hot-dampness Syndrome of RA (RA-Hot) according to the Guidelines of Diagnosis and Treatment of Rheumatoid Arthritis Disease and Syndrome Combination (2018) [2]. Patients meeting the diagnostic criteria were potentially eligible for the study according to the following criteria: (1) Age at 18 to 85 year old; (2) clearly diagnosed as RA and are classified as Cold/Hot-dampness syndromes after the doctor’s differentiation witness; (3) the included patients have stable vital signs, clear consciousness, and certain expression skills; (4) voluntary submission of written informed consent prior to enrollment. Subjects in any one or more of the following categories were excluded from our analysis: (1) the presence of type I or II diabetes; (2) active viral and/or bacterial infection; (3) the presence of osteoarthritis; (4) pregnant and lactating women; (5) those who use TCM-based prescriptions and formulas within 2 weeks; (6) those who have participated in other drug clinical trials within 2 weeks.

Venous blood was collected in the morning before breakfast from all the participants, and then sera were separated and stored at − 80 °C until use. Synovial fluid was extracted from the patients’ knee joint, and the synovial tissues were isolated and preserved with liquid nitrogen.

### Animal model establishment and sample preparation

Male Lewis rats (n = 48, 6–8-week-old, 200 ± 20 g in weight) were purchased from Beijing Vital River Laboratory Animal Technology Co., Ltd. (production license no. SCXK 2016-0006, Beijing, China). All animals were maintained under specific pathogen-free conditions in a temperature-controlled room at a constant temperature of 24 ± 1 °C with a 12 h light/dark cycle and had free access to the standard rodent diet and water ad libitum. The study was approved by the Research Ethics Committee of the Institute of Basic Theory of Chinese Medicine, China Academy of Chinese Medical Sciences, Beijing, China (SYXK 2016-0021, Beijing, China). All animal studies were treated in accordance with the guidelines and regulations for the use and care of animals of the Center for Laboratory Animal Care, China Academy of Chinese Medical Sciences.

A total of 48 rats were randomly divided into three groups: ① normal control group (n = 16); ② AIA-Cold (RA-Cold model group, n = 16); ③ AIA-Hot (RA-Hot model group, n = 16). Among them, AIA-Cold and AIA-Hot rat models were established and evaluated according to our previous studies [9, 11]. Urine and serum samples were collected 28th d after Freund complete adjuvant stimulation, after centrifugation at 3000 rpm for 15 min, the supernatant was reserved and stored at − 80 °C for subsequent metabonomics analysis.

### Clinically phenotypic data collection

The keywords of typical clinical and biochemical features of RA, such as “rheumatoid arthritis”, “vasculitis”, “anti-citrullinated protein antibody positivity”, “rheumatoid factor positive”, “joint swelling”, “joint stiffness”, “polyarticular arthritis”, “digital flexor tenosynovitis”, “swan neck-like deformities of the fingers”, “interphalangeal joint erosions”, “arthralgia”, “elevated erythrocyte sedimentation rate”, and “elevated circulating C-reactive protein concentration”, and the primary and secondary symptoms of cold and hot-dampness syndromes were used to collect the clinical symptom-related gene sets of RA-Cold and RA-Hot from the SoFDA platform (http://www.tcmip.cn/Syndrome/front/#/) [9, 11].

### Transcriptomics detection and data analysis

Gene expression profilings in the synovial tissues of the inflamed joints obtained from clinical samples were detected using the Agilent SurePrint Gene Expression Microarray (Agilent technologies, Santa Clara, CA, USA). Differential expression data analysis was performed using the DESeq2 R package (1.16.1). DESeq2 provides statistical routines for determining differential expression in digital gene expression data using a model based on negative binomial distribution. The *P*-values were adjusted using Benjamini and Hochberg’s approach for controlling the false discovery rate. Genes with an adjusted *P*-value < 0.05 found by DESeq2 were assigned as differentially expressed genes. We used the ClusterProfiler R package to test the statistical enrichment of differential expression genes in KEGG pathways, where corrected *P*-values below 0.05 were considered significantly enriched by differentially expressed genes.

### Metabonomic detection and data analysis

For synovial fluid samples, take 200 mg of thawed samples, add 50%/50% acetonitrile water extraction system of 1.5 mL ice, homogenize with electric homogenizer, centrifuge at 12,000 rpm and 4 °C for 20 min, take the upper layer, freeze–dry and re dissolve into liquid samples. For body fluid samples such as serum, synovial fluid and urine, an aliquot of 50 µL of thawed body fluid sample was deproteinized with 150 µL of MeOH: ACN (1:1, v/v) precooled to − 20 °C. After vortex mixed for 30 s and sonication for 10 min in an ice bath, samples were overnight at − 20 °C to improve protein precipitation and then centrifuged at 12,000 rpm for 15 min at 4 °C, 2 µL of supernatant was subjected to LC–MS and GC–MS analysis.

Then, metabonomic analysis was performed on a Shimadzu Nexera XR LC-20AD HPLC system equipment with SCIEX Triple TOF 5600+. In order to capture serum metabolic characteristics as comprehensively as possible, two different types of chromatographic columns [ACQUITY UPLC BEH amide column (2.1 × 150 mm, 1.7 μm) and ACQUITY UPLC BEH C18 column (2.1 × 100 mm, 1.8 μm)] were used for untargeted metabonomics analysis. Samples were injected into the GC–MS (Agilent 7890, Agilent, Palo Alto, CA, USA) system equipped a DB-5MS capillary column (Agilent 0.25 mm × 30 m × 0.25 μm, J&W Scientific, Folsom, CA, USA) and FID detector. The volume of injection was 1 µL, no streaming. Helium (99.9996%) was used as the carrier gas, the front inlet purge flow was 3 mL min^−1^, and the gas flow rate through the column was 1 mL min^−1^. The initial temperature was kept at 50 °C for 1 min, then raised to 300 °C at a rate of 10 °C min^− 1^, then kept for 9 min at 300 °C. The injection, transfer line, and ion source temperatures were 280 °C, 270 °C, and 220 °C, respectively. The energy was − 70 eV in electron impact mode. The mass spectrometry data were acquired in full-scan mode with the m/z range of 50–500 at a rate of 20 spectra per second after a solvent delay of 460 s.

The raw data of metabonomic analysis were imported to the Progenesis QI for peak alignment to obtain the peak area list and the identification result list. The nonparametric univariate method (Mann–Whitney–Wilcoxon test) was used to analysis metabolites that differed in abundance between the different subgroups corrected for false discovery rate (FDR) to ensure that the peak of each metabolite was reproducibly detected in the samples. Metabolites selected as biomarker candidates for further statistical analysis were identified based on variable importance in the projection (VIP) threshold of 1 from the tenfold cross-validated OPLS-DA model, which was validated at a univariate level with FDR < 0.05. The online HMDB data (https://hmdb.ca/), LIPIDMAPS (https://www.lipidmaps.org/), KEGG (https://www.kegg.jp/) and METLIN (https://metlin.scripps.edu) were used to align the molecular mass data to identify metabolites.

### Integrative network fusion-based multi-omics data analysis

The intersection of differentially expressed genes and the clinical symptom-related genes of RA-Cold and RA-Hot groups was used for constructing the “disease-syndrome-symptom” association network using the gene-gene interactions collected from STRING v11.0 database (https://www.string-db.org/). Then, Cytoscape 3.8.2 software and Origin 9.1 software were used to visualize the biomolecular network and calculate the topological features including node’s degree, betweenness and closeness for the selection of the key network targets according to our previous studies [9, 11, 17]. After that, David v6.8, an online bioinformatics analysis platform, (david.abcc.ncifcrf.gov/) was used to annotate and enrich the biological functions of the key network targets.

In addition, MetaboAnalyst platform (https://www.metaboanalyst.ca/) was used to collect and analyze the interactions between metabolites and genes associated with RA-Cold and RA-Hot. We input the key network targets and differential metabolites of the same Syndrome into the “Network Analysis” module of MetaboAnalyst platform and constructed the integrated metabolite-protein networks, which were used to illustrate the associations between the metabolic and transcriptional characteristics of RA-Cold and RA-Hot, and subsequently screening the candidate biomarkers of the two groups.

### Enzyme-linked immunosorbent assay (ELISA)

The expression levels of 5-hydroxy-l-typtophan (5-HT, Cat. No. ml057425), Glutaminase (GLNS, Cat. No. ml063074), Sphingosine 1-phosphate (S1P, Cat. No. ml038623), Interleukin-17F (IL17F, Cat. No. ml063074) and Interleukin-4-Induced-1 (IL4I1, Cat. No. ml025219) proteins in the serum and synovial fluid of RA patients were estimated using the ELISA kit (ML Bio, Shanghai, China) according to the manufacturer’s instructions. The absorbance was measured using a Multiskan™ GO microplate spectrophotometer (Thermo Fisher Scientific, Waltham, MA, United States).

### Statistical analyses

All experiments were repeated at least three times. The experimental results are expressed as means and standard deviations (S.D.). One-way analysis of variance was used for analysis between groups, and Student’s *t*-tests were used for pairwise comparisons. Results with P values less than 0.05 were considered statistically significant. GraphPad Prism 8.0 software (GraphPad, CA, USA) was used for graphics. The Mann–Whitney–Wilcoxon test or Welch’s *t*-test, was performed to measure the significance of each peak, with results adjusted for multiple testing using FDR correction. The principal component analysis (PCA), partial least squares-discriminant analysis (PLS-DA) and variable importance in the projection (VIP_plsda_) were calculated using the statTarget package. Spearman’s rank correlation was used for a measure of correlation between two variables. The nodes represent clinical parameters or metabolites and genes, and two nodes were connected if they were significantly correlated (adjusted *P*-value < 0.05 and r-value > 0.2). The ordinal regression was performed by using the ordinalgmifs package for fitting an ordinal response model [18]. Coupling the receiver operating characteristic curve (ROC) with its area under the curve (AUC), a widely used method to estimate the diagnostic potential of a classifier in clinical applications, was performed using the pROC package [19]. Performance metrics (AUC, Accuracy, Precision, Specificity, Sensitivity and F1-measure) are calculated based on confusion matrix analysis. All packages were implemented using the freely available R language.

## Results

### Clinical data and patients’ characteristics

The demographic information of the study participants including 38 RA-Cold and 45 RA-Hot patients was summarized in Table 1 and Additional file 1. These samples were randomly divided into two independent cohorts. Among them, the discovery cohort, including 26 RA patients (10 with RA-Cold and 16 with RA-Hot) and 20 normal controls (NCs) was used for metabonomic and transcriptomic detections and data analysis; The validation cohort, including 57 RA patients (28 with RA-Cold and 29 with RA-Hot) and 27 NCs, was used for biochemical experimental validation of the candidate biomarkers. The statistical results in Table 1 showed that there were significant differences in age, ESR, and CRP indicators between the NC and RA patient groups (all P < 0.05), while there were no differences with statistical significance in gender, age, and any clinical biochemical parameters between the RA-Cold and RA-Hot groups.

**Table 1:** Clinical and demographic characteristics of the study participants

Characteristics	RA-Cold (n = 38)	RA-Hot (n = 45)	NC (n = 47)
Female: male	7.25	6.5	0.81
Age, mean (SD), years	53.1 (11.3)**	53.1 (14.3)**	38.2 (19.8)
RF, mean (SD), IU/mL	105.6 (127.1)	94.4 (94.9)	–
ESR, mean (SD), mm/h	48.3 (33.5)**	55.9 (28.2)**	9.6 (8.4)
CRP, mean (SD), mg/L	33.1 (34.6)**	39.7 (39.6)**	16.6 (17.5)
DAS28-ESR, mean (SD)	5.1 (1.5)	5.4 (1.2)	–
DAS28-CRP mean (SD)	5.1 (1.1)	5.3 (1.1)	–

*RF* rheumatoid factor, *ESR* erythrocyte sedimentation rate, *CRP* C-reactive protein, *DAS28-ESR* disease activity score 28-joint count erythrocyte sedimentation rate, *DAS28-CRP* disease activity score 28-joint count C-reactive protein

**Comparison with the NC group, *P* < 0.01

### Phenomics and transcriptomics analyses of RA-Cold and RA-Hot patients

**Fig. 2:**
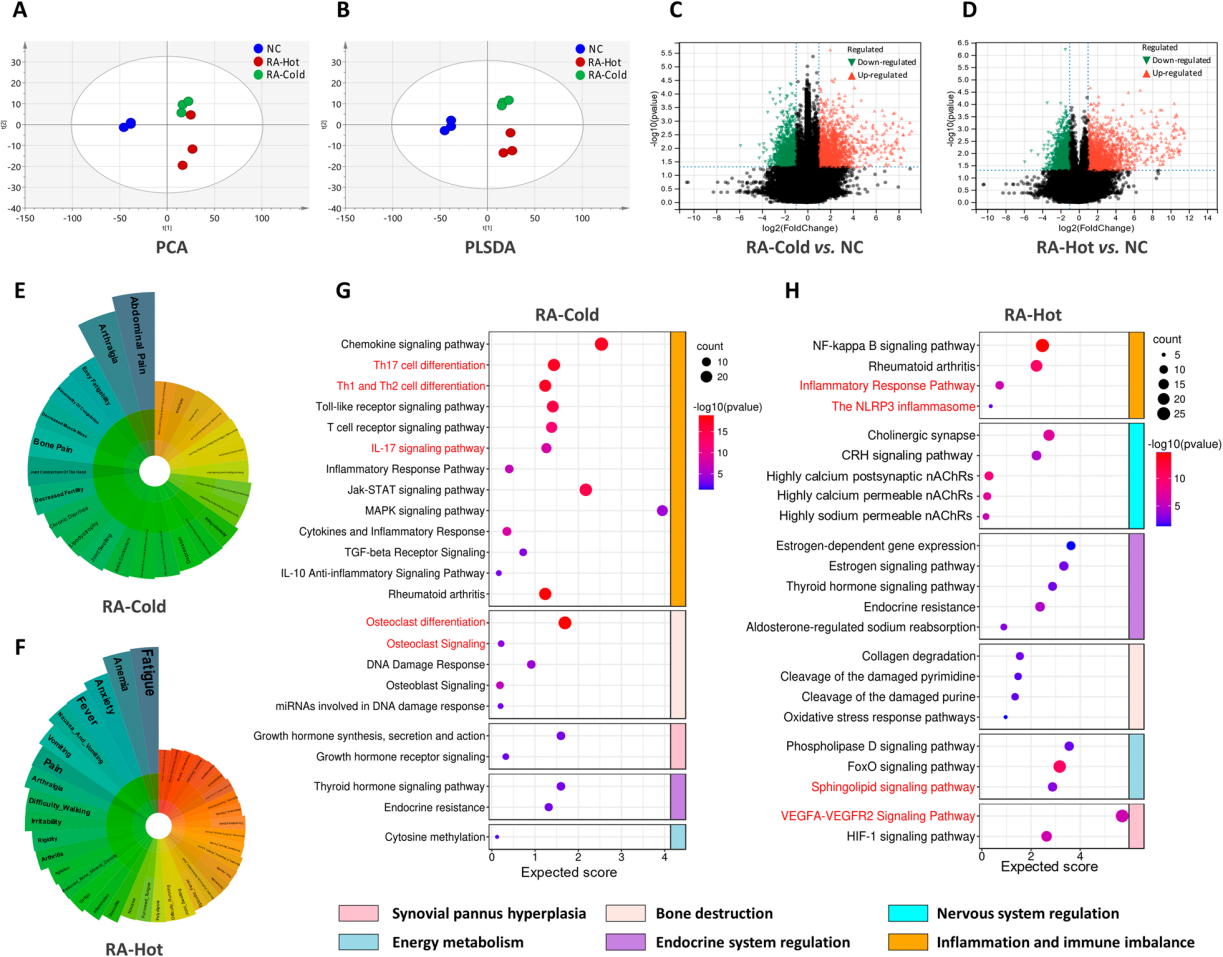
Phenomics and transcriptomics analysis of RA-Cold and RA-Hot patients. PCA and PLS-DA scatter plots (**A**, **B**) and differential gene volcano plots (**C**, **D**) of transcriptomics profilings of RA-Cold and RA-Hot. Polar bars of clinical phenotypes (**E**, **F**) and biological function enrichment plots of RA-Cold and RA-Hot key network targets (**G**, **H**).

To analyze whether RA-Cold and RA-Hot have differences and associations in gene expression, we carried out the multivariate and univariate analyses to identify differentially expressed genes from clinical synovial tissue samples. The well-established PCA and PLS-DA demonstrated excellent separation of NCs, RA-Cold, and RA-Hot based on transcriptomic profiles (Fig. 2A, B). By applying the cutoffs of VIP_plsda_ > 1, fold change > 2 or < 0.5, and adjusted *P*-value < 0.05, the differentially expressed genes (DEGs) were selected. A total of 842 DEGs were identified in RA-Cold versus NC, 534 of which were upregulated and 308 were downregulated (Fig. 2C). A total of 940 DEGs were identified in RA-Hot versus NC, 569 of which were upregulated and 371 were downregulated (Fig. 2D). The representative primary and secondary symptoms of RA-Cold and RA-Hot were searched through relevant databases as keywords. We screened and sorted out 478 (for RA-Cold) and 1171 (for RA-Hot) clinical symptom-related gene sets of the two syndromes, respectively.

Following the “disease-syndrome-symptom” association network analysis, a total of 1240 (for RA-Cold) and 1978 (for RA-Hot) key network targets were identified due to their topological importance. As shown in Fig. 2E, F, the key network targets of RA-Cold and RA-Hot were significantly associated with 66 and 78 clinical phenotypes of the corresponding disease and syndromes, respectively (all *P* < 0.05). Among them, the clinical phenotypes associated with the key network targets of RA-Cold mainly include arthralgia, joint contracture of the hand, bone pain, decreased sensory nerve conduction velocity, etc., while that of RA-Hot mainly include anxiety, fever, irritability, difficulty walking, vasculitis, etc., in line with the clinical characteristics of the corresponding patients. Functionally, 179 Wiki-based, 102 Reactome-based and 130 KEGG-based pathways were associated with RA-Cold key network targets, 188 Wiki-based, 133 Reactome-based, and 148 KEGG-based pathways were associated with RA-Hot key network targets (see Additional file 2). Notably, most of the RA-Cold-related pathways, such as Th17 cell differentiation, IL-17 signaling pathway, Osteoclast differentiation, have been indicated to be involved into the imbalance of inflammation and immune, as well as bone destruction during the development and progression of RA [20, 21]. In contrast, VEAGFA–VEGFR2 signaling, sphingolipid signaling and NLRP3 inflammasome, enriched by most RA-Hot kay network targets, have been reported to play roles in synovial pannus hyperplasia, energy metabolism (especially lipid metabolism), and nervous system regulation of RA patients [22–24] (Fig. 2G, H). Data referenced in Fig. 2 and Additional file 2 are available in Gene Expression Omnibus (GEO) with the accession No. GSE205962.

### Metabolic profilings of serum and synovial fluid samples obtained from RA-Cold and RA-Hot patients

**Fig. 3:**
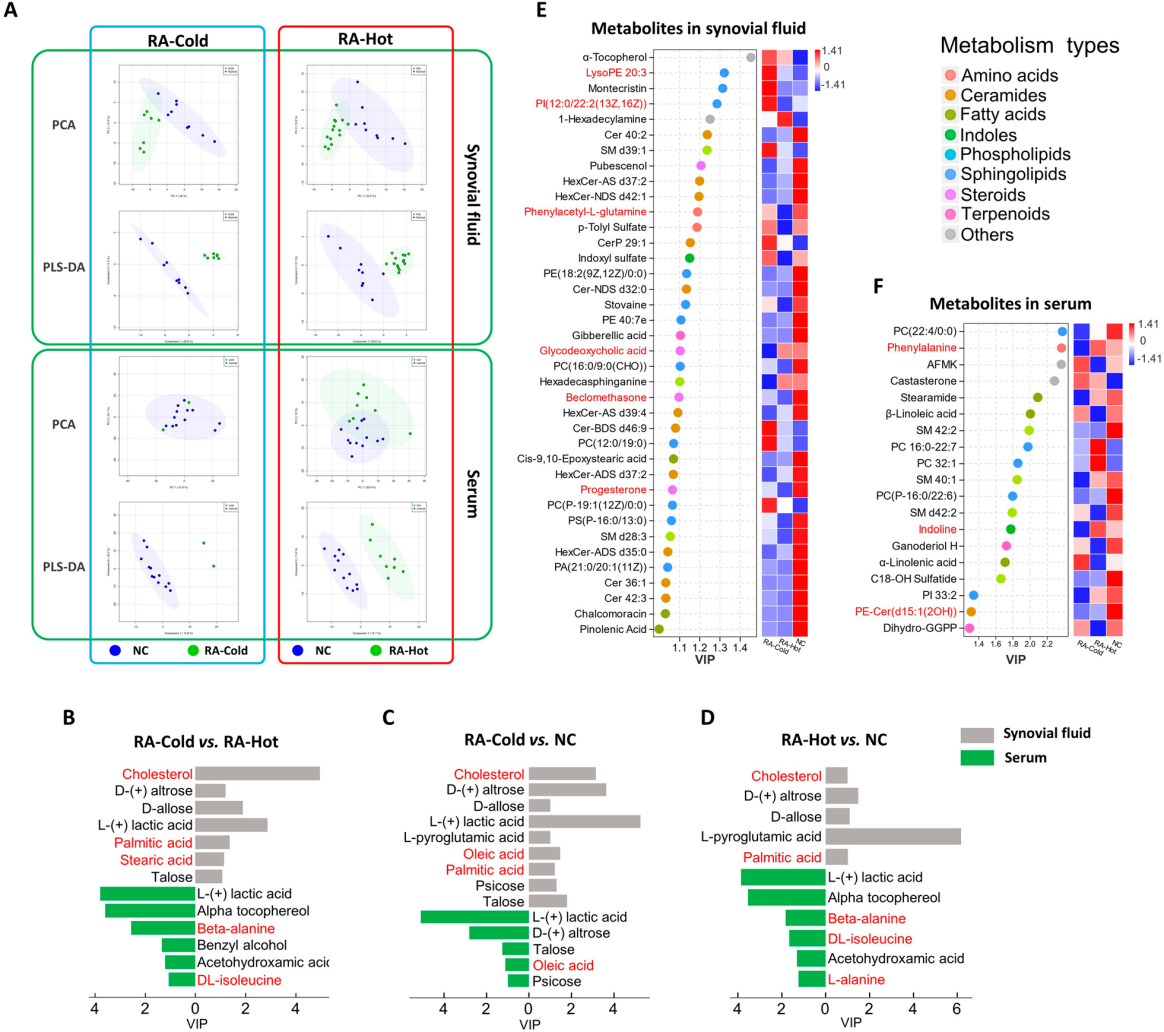
Metabolic profilings of serum and synovial fluid samples obtained from RA-Cold and RA-Hot patients. **A** PCA and PLS-DA score plots of metabolic profiling data for RA-Cold and RA-Hot groups. **B**–**D** Potential metabolic markers detected by GC–MS, sorted according to VIP values, are marked in bright blue as differential metabolites in synovial fluid, and dark blue as differential metabolites in serum. Dotrod-heatmap of potential metabolic markers in synovial fluid (**E**) and serum (**F**) detected by LC–MS. On the left is a scatter plot, which represents the VIP values and categories of metabolites, with different colors representing different metabolite categories. The right heat map represents the expression of metabolites in different groups. The red and blue markers represent the content. The redder (blue) the color is, the higher the content (lower). Both final differential metabolites were selected by VIP_plsda _> 1, fold change > 2 or < 0.5, *P*-value < 0.05

To determine the potential of system-wide identification and diagnosis of RA patients with two syndromes, we performed two metabolic profiling analyses using serum and synovial fluid samples obtained from RA-Cold and RA-Hot patients by GC–MS and LC–MS. As shown in Fig. 3A, the metabolic profiles of the normal control, RA-Cold and RA-Hot groups were well separated in the PLS-DA score plots. Compared with the NC group, there were 9 differential metabolites in the synovial fluid and 5 in the serum of the RA-Cold group, and 5 differential metabolites in the synovial fluid and 6 in the serum of the RA-Hot group (VIP_plsda _> 1, Fig. 3B–D), an additional table file shows this in more detail (see Additional file 3). In the comparison between RA-Hot and RA-Cold groups, 7 differential metabolites in the synovial fluid and 6 in the serum were identified (VIP_plsda _> 1). Especially, cholesterol was found to be a differential metabolite in synovial fluid samples of both RA-Cold and RA-Hot patients comparing with NC samples. Differences in fatty acids such as oleic acid, palmitic acid and stearic acid were observed in the comparison of RA-Cold and NC groups, while there were significant differences in fatty acids and amino acids such as β-alanine and isoleucine between RA-Hot and NC groups, implying steroids, fatty acids and amino acids might be potential disease-identifying metabolic markers.

Due to the few metabolites detected by GC–MS, the specific situation still needs to be further analyzed in combination with clinical LC–MS metabolic data. A total of 119 differential metabolites in the synovial fluid and 417 in the serum were detected using the biological samples obtained from RA patients based on LC–MS (RA-Cold vs. NC & RA-Hot vs. NC & RA-Hot vs. RA-Cold), and 535 differential metabolites were obtained after de-redundancy (see Additional file 4). By applying the cutoffs of VIP_plsda _> 1, fold change > 2 or < 0.5, and *P*-value < 0.05, a total of 53 (19 in serum and 34 in synovial fluid) differential metabolites were screened (Fig. 3E, F) and classified as lipid metabolites, including sphingolipids, phospholipids, and steroids, which have been reported to be crucial components in amino acid metabolism, sphingolipid metabolism, and steroid metabolism pathways.

### Metabolic profilings of serum and urine samples obtained from AIA-Cold and AIA-Hot rats

**Fig. 4:**
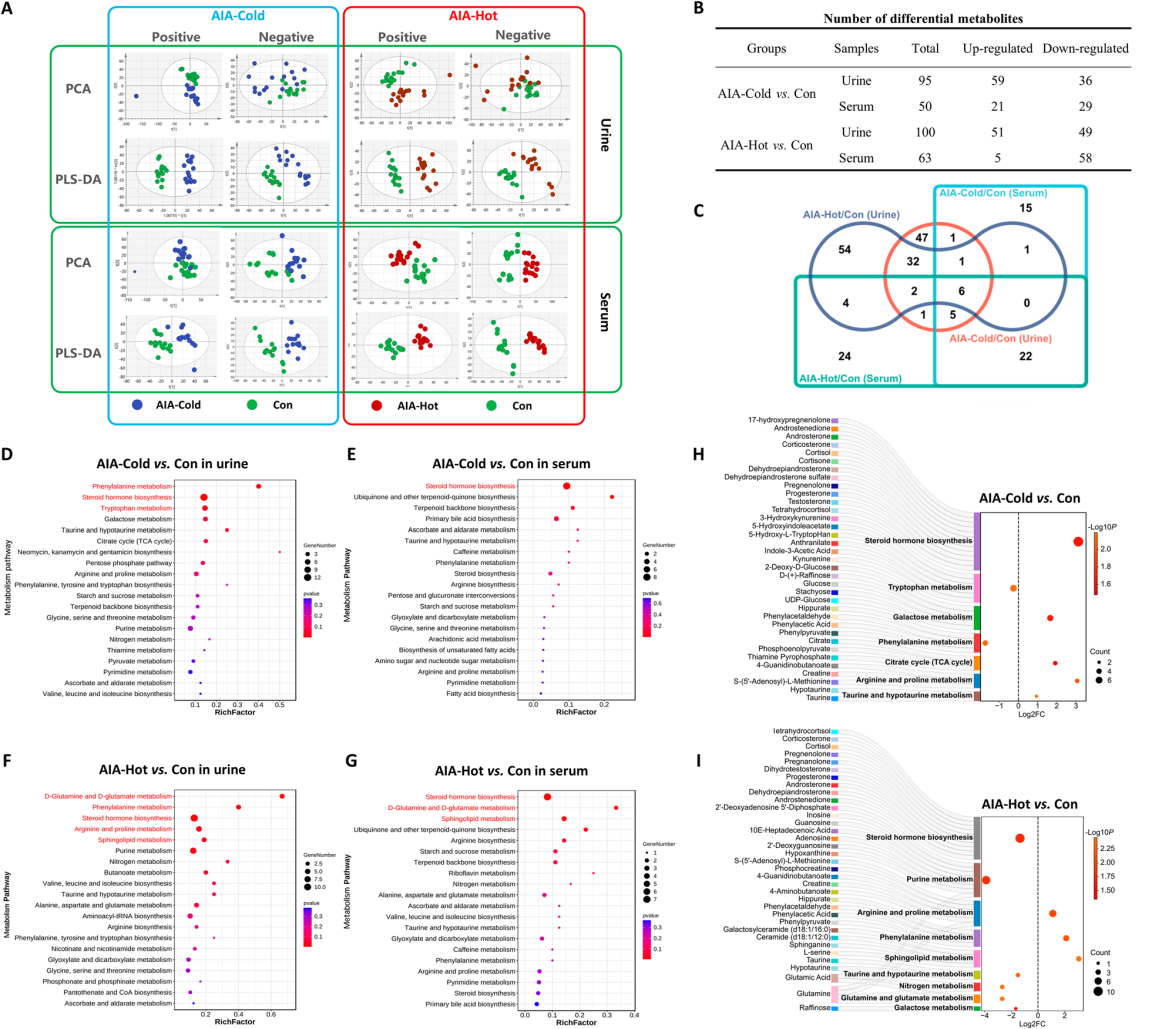
Metabolic profilings of serum and urine samples from AIA-Cold and AIA-Hot rats. **A** PCA and PLS-DA score plots of metabolic profilins of serum and urine samples from AIA-Cold and AIA-Hot rats. **B** Number of differential metabolites in rat urine and serum samples of AIA-Cold and AIA-Hot groups. **C** Differential metabolites Venn diagram, different colors represent different combinations of comparisons. **D**–**G** Enrichment bubble plots based on differential metabolites in rat urine and serum samples of AIA-Cold and AIA-Hot groups. **H**, **I** Sankey dot plots of the representative metabolic pathways of AIA-Cold and AIA-Hot, with differential metabolites involved in the pathway on the left, and fold-change (FC)-related bubble plots of the metabolic pathways on the right

Following the metabolic profiling identification of clinical samples, we further performed metabonomic detection using the serum and urine samples from AIA-Cold and AIA-Hot rats to investigate the metabolic differences between the two groups. In our previous studies [9, 11], we successfully established AIA-Hot and AIA-Cold rat models which simulated the clinical manifestations and pathological changes of wind-cold-dampness and wind-hot-dampness stimulating RA, respectively. Since the multivariate analysis based on metabolic data of experimental animals did not show a satisfying separation between AIA-Cold and AIA-Hot groups in both positive and negative ion modes, a supervised PLS-DA model was established and output the well separated results as shown in Fig. 4A. Then, the raw metabolic data in both positive and negative ion modes were integrated, removed redundancy and screened for the identifying the differential metabolites. Compared with normal rats, a total of 95 (including 59 up-regulated and 36 down-regulated) and 50 (21 up-regulated and 29 down-regulated) differential metabolites were respectively identified in urine and serum samples of AIA-Cold rats, while there were 100 (51 up-regulated and 49 down-regulated) and 63 (5 up-regulated and 58 down-regulated) differential metabolites identified in urine and serum samples of AIA-Hot rats, respectively (Fig. 4B, C), an additional table file shows this in more detail (see Additional file 5). Mechanically, the above differential metabolites were significantly associated with various metabolic pathways involved into RA progression according to the results analyzed by MetaboAnalyst (www.metaboanalyst.ca). Among them, Phenylalanine metabolism and Tyrosine metabolism may be the representative metabolic pathways of AIA-Cold group (Fig. 4D, E), while glutamine and glutamate metabolism, Sphingolipid metabolism as the representative metabolic pathways of AIA-Hot group (Fig. 4F, G). Interestingly, Steroid hormone biosynthesis was a common enriched pathway for both groups, in line with the clinical findings that several differential steroid metabolites were identified based on in serum and synovial fluid samples from both RA-Cold and RA-Hot. Moreover, a total of 35 differential metabolites were involved into the RA-Cold-related pathways, such as steroid hormone biosynthesis, tryptophan metabolism and phenylalanine metabolism Among them, Progesterone, 5-hydroxy-l-typtophan (5-HT), Phenylpyruvate and Creatine were identified as candidate metabolic markers of AIA-Cold due to the significant differences and functional importance (Fig. 4H). In contrast, there were 38 differential metabolites participated in various RA-Hot-related pathways, such as steroid hormone biosynthesis, sphingolipid metabolism and Glutamine and glutamate metabolism. Among them, Progesterone, Sphinganine, and Glutamine were identified as candidate metabolic markers of AIA-Hot due to the significant differences and functional importance (Fig. 4I). These results are consistent with trends in clinical metabolic profiling and provide a biological discovery and validation paradigm for RA biomarker identification.

### Integrative network fusion-based multi-omics data analysis for candidate biomarker identification of RA-Cold and RA-Hot

**Fig. 5:**
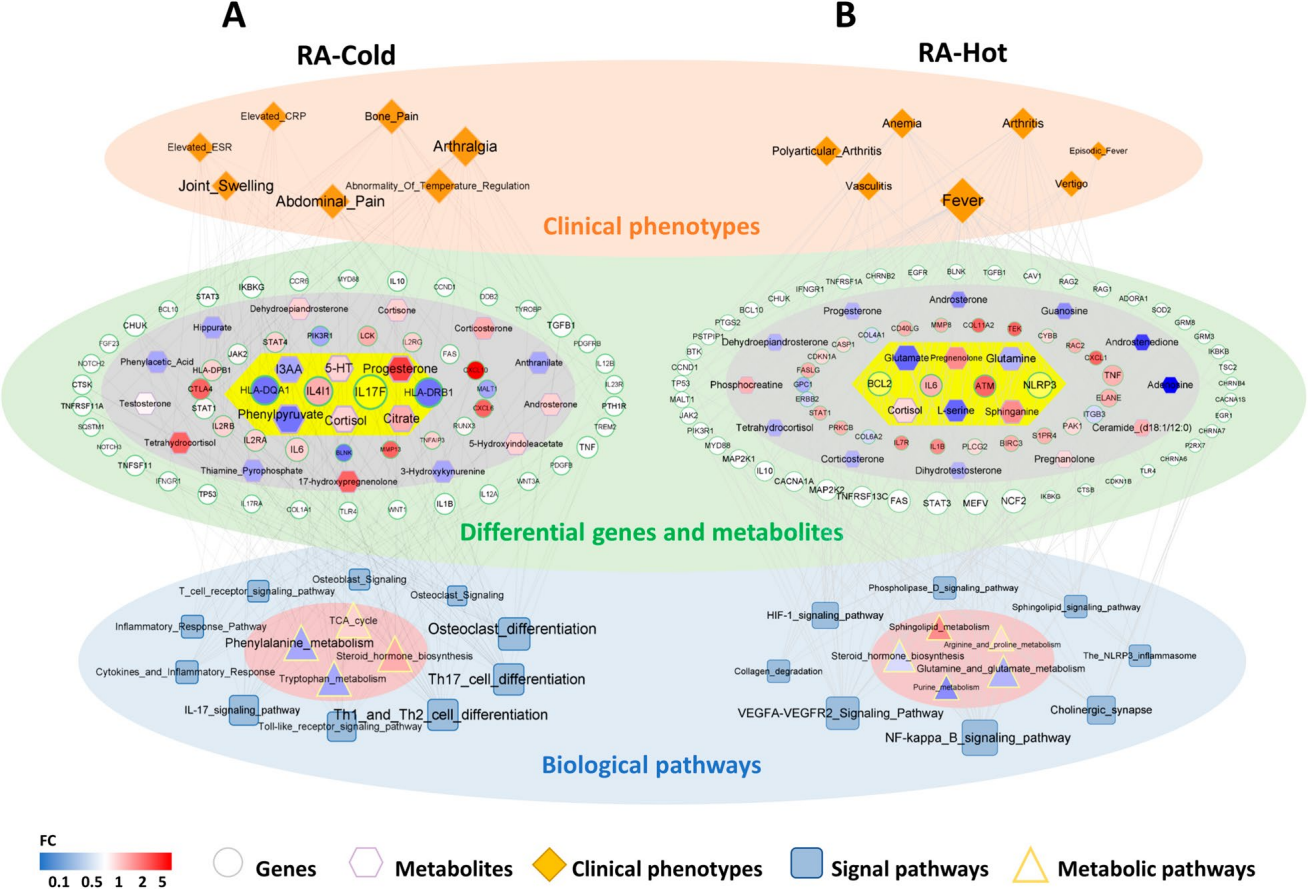
Integrative network fusion-based multi-omics data analysis for identification candidate biomarkers of RA-Cold and RA-Hot. Multi-level “Disease-Syndrome-Gene-Metabolite-Pathway” network diagram for RA-Cold biomarkers (**A**) and RA-Hot biomarkers (**B**). The orange diamond nodes refer to the representative clinical symptoms of RA-Cold and RA-Hot patients. The circle nodes refer to the key network target genes of RA-Cold and RA-Hot groups, in which red and blue nodes refer to the up-regulated and down-regulated genes according to the clinical transcriptomic profiling, and white nodes refer to the symptom-related genes (no differential changes detected in the clinical transcriptomic profiling), and the yellow part in the center represents the candidate biomarkers. Hexagons nodes refer to the differential metabolites, among which red and blue hexagon nodes respectively refer to up-regulated and down-regulated metabolites. The square and triangle nodes refer to the signal pathways enriched by the key network target genes and differential metabolites, respectively

According to the links among clinical symptoms and the related genes, differentially expressed genes and metabolites, and their enriched pathways, we constructed a multi-level network of “Disease-Syndrome-Symptom-Gene-Metabolite-Pathway” using the visualization software Cytoscape 3.10.1 (Fig. 5). The key RA-Cold network targets, including HLA-DRB1, HLA-DPB1, IL4I1, IL17F were significantly associated with the typical clinical symptoms such as arthralgia, joint swelling, bone pain, etc., and also participated into the corresponding signal pathways such as Th1 and Th2 cell differentiation, Th17 cell differentiation, IL17 signaling, osteoclast differentiation, etc., which were involved by several differential metabolites, including phenylpyruvate (down regulation), 5-HT (down regulation), cortisol (up regulation) via regulating phenylalanine metabolism, tryptophan metabolism and steroid biosynthesis. In contrast, the key RA-Hot network targets including VEGFA (up regulation), ATM (up regulation), CASP1 (down regulation), L-serine (down regulation), sphinganine (up regulation), glutamine (down regulation) were related to the representative symptoms of RA-Hot patients, such as anemia, fever, vasculitis, vertigo, etc., and also enriched into VEGFA-VEGFR2 signaling, NLRP3 inflammasome, sphingolipid signaling, steroid biosynthesis, glutamate and glutamine metabolism, sphingolipid metabolism pathway, etc. On this basis, IL17F, 5-HT and IL4I1, while the key substrates for sphinganine and glutamine, Sphingosine 1-phosphate (S1P) and glutaminase (GLNS), were identified as candidate biomarkers of RA-Cold and RA-Hot, respectively.

### Experimental validations of candidate biomarkers of RA-Cold and RA-Hot using an independent clinical cohort

**Fig. 6:**
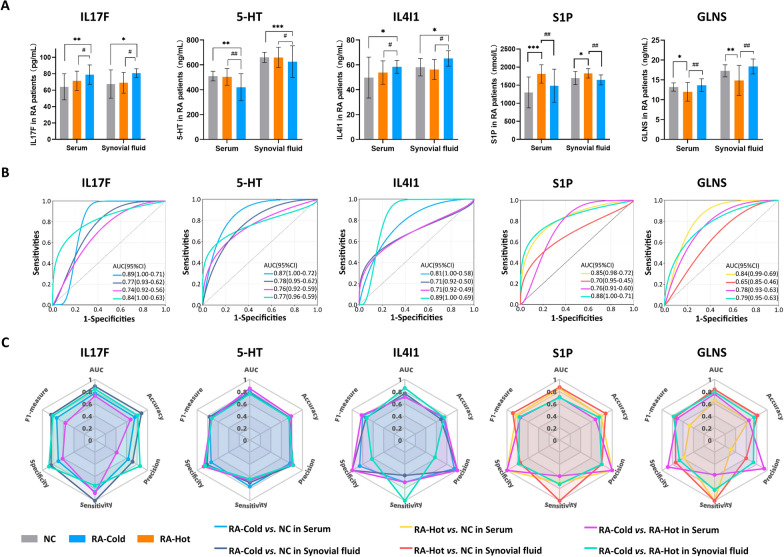
Experimental validations of candidate biomarkers of RA-Cold and RA-Hot using an independent clinical cohort. Boxplots (**A**) and ROC (**B**) analysis of five biomarkers with differential levels among NC, RA-Cold and RA-Hot groups. Radar Charts of performance metrics (AUC, Accuracy, Precision, Sensitivity, Specificity and F1-measure) for biomarkers (**C**). *P < 0.05, **P < 0.01, ***P < 0,001 versus NCs group, ^#^*P* < 0.05, ^##^*P* < 0.01, RA-Cold versus RA-Hot group. Data are presented as mean ± SD and analyzed by Wilcoxon–Mann U test with FDR control

To verify the prediction performance of the candidate biomarkers (IL17F, 5-HT and IL4I1 for RA-Cold; S1P and GLNS for RA-Hot) in clinical diagnosis of the two groups, we carried out ELISA and ROC analyses to detect the levels of the candidate biomarkers in serum and synovial fluid samples from the independent clinical cohort. As shown in Fig. 6A, B, IL17F, 5-HT and IL4I1 levels were significantly different in RA-Cold patients than that in NC and RA-Hot patients (all *P* < 0.05, AUC > 0.7), whereas the differences in the content of S1P and GLNS in RA-Hot patients were significantly different from those in NC group and RA-Cold patients (all *P* < 0.05, AUC > 0.7), indicating their potential role in the classification of the two RA patient groups. To avoid an excessively high chance of false positive predictions in clinics, we comprehensively evaluated the accuracy, precision, specificity, sensitivity and F1 measure of the above candidate markers (Fig. 6C). The results indicated that there may be differences in the discriminative strengths of different markers in serum and synovial fluid samples, and some markers such as IL17F and S1P had good scores in both samples, while some markers are more advantageous in the detection of specific samples, such as 5-HT in synovial fluid and IL4I1 in serum. Similarly, it is inferred that GLNS may require to be combined with both the serum and synovial fluid levels to obtain the best performance.

## Discussion

Accurate diagnosis of RA-Cold and RA-Hot is crucial for improving the treatment efficacy and prognosis of RA patients, but there is still a lack of efficient diagnostic markers. To the best of our knowledge, this is the first study performing an integrated network fusion-based multi-omics investigation to explore the biological basis of RA-Cold and RA-Hot, and to identify a list of candidate biomarkers for the classification of the two RA groups. In terms of macroscopic clinical symptoms, RA-Cold is characterized by joint swelling, pain and contracture, which are different from those of RA-Hot, including inflammatory response, fever, irritability and vertigo. At the microscopic molecular level, RA-Cold-related genes was mainly involved into amino acids (phenylalanine, tryptophan) metabolic pathway and IL-17 signaling pathway; while the development and progression of RA-Hot were mainly regulated by sphingolipid metabolism, glutamine metabolic pathway, NLRP3 and VEGF angiogenesis signal pathway. Notably, the steroid biosynthesis was a regulatory pathway shared by both RA-Cold and RA-Hot, but displayed in an opposite regulatory mode, that is, in RA-Cold, steroid metabolites such as cortisol, androsterone and 17-hydroxypregnenolone showed an overall upward-regulated trend. On the contrary, androstenedione, progesterone, dihydrotestosterone and other steroid metabolites showed a downward trend in RA-Hot. Moreover, we identified IL17F, 5-HT and IL4I1 as the candidate biomarkers for RA-Cold, and S1P and GLNS as the candidate biomarkers for RA-Hot, which were all verified using an independent clinical cohort and provided a reference for clinical differentiation and diagnosis.

Growing evidence indicates the imbalance of “inflammation-immune” system may play an important role in the occurrence and development of RA [25–27]. Due to the long-term exposure to cold and humidity for RA-Cold patients [28], accumulating studies have reported that the energy shortage may be caused by low temperature environmental stimulation [29, 30], subsequently leading to the dysregulation of energy metabolism in RA-Cold. Consistently, our previous studies demonstrated that a clinically prescription for the treatment of RA-Cold Wutou Decoction (WTD) may effectively relieve the disease severity by regulating the ALOX15B-PPAR-γ-PTGS2-FGF2-IL1β-cJUN-MM13-TGFβ1 signaling axis which may be associated with the balance maintenance of “inflammation-immune” system, the production of body heat and the regulation of energy metabolism [9]. Spiljar et al. [31] found that hypothermic stimulation may modulate T cell immunity and metabolic phenotype in mice, thereby altering the energy balance between metabolic adaptation and autoimmunity in mice. Joo et al. [32] reported that the production of pro-inflammatory cytokines including IL-12, IL-17 and cytokines in mouse lung tissues was increased and the inflammatory response was seriously aggravated after cold stimulation of LPS induced acute lung injury mice. These findings suggest that T cell immunity may be disturbed under the cold stimulation, in accord with our data that “IL-17 signaling pathway” and “T cell differentiation” may be associated with the main pathological changes of RA-Cold, such as RA synovial cell migration, autophagy, and chemokine production [20, 33–35]. In addition, we also revealed several metabolic pathways such as “Tryptophan metabolism” and “Phenylalanine metabolism” showing differences in RA-Cold group comparing with the normal and RA-Hot groups. As one of the main metabolic pathways to maintain the basic functions of the body, tryptophan and phenylalanine metabolism has been reported to be closely related to T cell immunity [36–38]. During this process, serotonin (also known as 5-HT) is an important neurotransmitter produced by tryptophan and phenylalanine metabolism, and plays a role in maintaining the immune system since various immune cells such as T cells, dendritic cells, and monocytes often express 5-HT receptors [39, 40]. Yasmine et al. [41] observed the involvement of 5-HT in wild-type and Tph1−/− collagen-induced arthritis mice, and found that peripheral 5-HT levels in these mice were significantly reduced, and 5-HT could play an important role in RA by regulating Th17/Treg cell balance and osteoclastogenesis. IL4I1 is a glycosylated protein with amino acid oxidase activity, with the highest catalytic activity towards phenylalanine, followed by tryptophan and tyrosine [42]. The genes encoded by IL4I1 are located in the susceptible regions of various autoimmune diseases and are mainly expressed in human immune cells. It is speculated that IL4I1 may be closely related to the occurrence of autoimmune diseases [43]. It has been reported that IL4I1 may inhibit the proliferation of T cells and promote the production of inflammatory cytokines and chemokines such as IL-2 through its metabolism of the strong oxidizing substance H_2_O_2_ produced by phenylalanine [44]. Another study reported that l-tryptophan analogs could partially alleviate the inhibitory effect of IL4I1 on T cell proliferation [45], suggesting that IL4I1 may play a pivotal role in T cell immunity, phenylalanine metabolism, and tryptophan metabolism. Consistently, the current study observed significant differences of IL17F, 5-HT and IL4I1 levels in RA-Cold samples and their diagnostic performance were also verified via the independent clinical cohort test.

Patients at the active stage of RA often display the clinical symptoms of RA-Hot, accompanied with severe immune and inflammatory responses [28]. An increasing number of studies indicated that the NLRP3 inflammasome may be closely related to the pathogenesis of RA [46, 47]. NLRP3 may be activated by TLR4 signaling [48, 49], resulting in the formation of intracellular inflammasome complexes with the adaptor protein ASC and caspase-1–dependent cleavage and secretion of IL-1β [50], which is a well-characterized outcome of TLR and inflammasome cooperation [46]. Several studies found the high expression of NLRP3 mRNA and protein in monocytes/macrophages, fibroblast-like synoviocytes (FLS), dendritic cells, and neutrophils from RA patients [51–54]. Similarly, our previous studies also demonstrated that a clinically prescription for the treatment of RA-Hot Baihu Guizhi Decoction (BHGZD) may effectively suppress the TLR4-mediated NLRP3 inflammasome activation during RA-Hot progression [11]. As the activation of the NLRP3 inflammasome and the occurrence of pyroptosis, the metabolic levels of the body may also be disturbed, especially the changes in lipid metabolites [55]. Ceramide is the main metabolite of the sphingolipid metabolic pathway and one of the important synthetic substrates for NLRP3, which can be supplemented by S1P through ceramidase and sphingosine kinase [56]. According to our metabonomics data, we found that the contents of “Sphingolipid metabolism” related differential metabolites were significantly increased. Among them, S1P had significant differences in RA-Hot comparing with normal and RA-Cold groups, which were also verified in serum and synovial fluid from the independent clinical cohort. In addition to the inflammatory response and immune function, the regulation of synovial angiogenesis in RA has always been a hot spot in the current research field. Due to the disturbance of various angiogenesis regulatory factors in RA, the infiltration of a large number of inflammatory factors may induce excessive synovial angiogenesis. Maintaining and promoting the formation of pannus is one of the initiating factors causing joint lesions and cartilage destruction [57]. Accordingly, our previous finding also indicated that BGHZD may have the potential to inhibit excessive angiogenesis of synovium in RA-Hot via regulating VEGF/VEGFR2/PI3K/AKT signaling pathway [58]. In this study, the VEGF signaling pathway was significantly enriched by the key network targets of RA-Hot. Interestingly, David et al. [59] performed a comparative analysis of indole-3-carbaldehyde (IAld) and indole-3 acetic acid (I3AA) to understand how they affect RA pathogenesis by using established cell-based models, and found that IAld and I3AA showed large functional differences. Although IAld has anti-inflammatory activity [60], it may exert pro-osteoclastogenic and pro-angiogenic effects. In contrast, I3AA only showed a significant anti-angiogenic activity. Herein, the levels of the two indole metabolic derivatives were significantly elevated in RA-Hot samples compared with that in normal and RA-Cold groups, suggesting the overactivation of VEGF angiogenesis in RA-Hot. As the key invertase of glutamine, GLNS can deaminate glutamine into glutamic acid and then transformed to α-ketoglutarate, which makes it enter other metabolic pathways, such as tricarboxylic acid (TCA) cycle and lipid synthesis [61, 62], to fully exert its biological functions. Moreover, GLNS has been reported to play a key role in the growth of RA-FLS. Takahashi et al. [63] found that the expression of GLNS was increased in RA-FLS compared to osteoarthritis (OA)-FLS, and the proliferation of RA-FLS might be suppressed under the condition of glutamine deprivation, suggesting that glutamine metabolism may be involved in the pathogenesis of RA, and GLNS may play an important role in regulating the proliferation of RA-FLS. In line with these previous findings, our data also identified GLNS as a novel biomarker of RA-Hot.

Notably, four types of tissue samples including serum, urine, synovial tissue, and synovial fluid were detected at the same time, which may not only reflect the whole-body state in the process of RA occurrence and progression, but also show the pathological and molecular changes of local inflamed joints. In addition, this integrative network fusion-based multi-omics study may be more comprehensive and systematical to identify the molecular differences between RA-Cold and RA-Hot groups, which may be conducive to the discovery of new biomarkers. However, the clinical sample size of this study is relatively small due to the epidemic prevention and control, and our strict inclusion and exclusion criteria.

In conclusion, the current study may offer evidence that the development and progression of RA-Cold may be associated with T cell immune regulation, phenylalanine and tryptophan metabolism pathway, while RA-Hot may be mainly related to the disturbance of sphingolipid metabolism, glutamine metabolism, NLRP3 and VEGF angiogenesis signal pathways. More importantly, we also identified IL17F, 5-HT and IL4I1 as candidate biomarkers of RA-Cold, and S1P and GLNS as candidate biomarkers of RA-Hot. These findings may be of great clinical significance to accurately differentiate of RA patients with different syndromes and help them receiving more accurate and effective treatment options.

## Supplementary Information

**Additional file 1: Table S1.** Clinical data and patient characteristics.

**Additional file 2. Pathways enriched by key network targets of RA-Cold and RA-Hot: Table S2.1.** WikiPathways enriched by key network targets of RA-Cold. **Table S2.2.** Reactome Pathways enriched by key network targets of RA-Cold. **Table S2.3.** KEGG-Pathways enriched by key network targets of RA-Cold. **Table S2.4.** WikiPathways enriched by key network targets of RA-Hot. **Table S2.5.** Reactome-Pathways enriched by key network targets of RA-Hot. **Table S2.6.** KEGG-Pathways enriched by key network targets of RA-Hot.

**Additional file 3: Table S3.** Differential metabolites of serum and synovial fluid samples from RA-Cold and RA-Hot patients detected by GC/MS.

**Additional file 4. Differential metabolites of serum and synovial fluid samples from RA-Cold and RA-Hot patients detected by LC/MS: Table S4.1.** Total differential metabolites of serum and synovial fluid samples from RA-Cold and RA-Hot patients detected by LC/MS. **Table S4.2.** Significantly differential metabolites of serum and synovial fluid samples from RA-Cold and RA-Hot patients detected by LC/MS.

**Additional file 5. Significantly differential metabolites of urine and serum samples from AIA-Cold and AIA-Hot rats detected by LC/MS: Table S5.1.** Significantly differential metabolites of urine samples from AIA-Cold rats detected by LC/MS. **Table S5.2.** Significantly differential metabolites of serum samples from AIA-Cold rats detected by LC/MS. **Table S5.3.** Significantly differential metabolites of urine samples from AIA-Hot rats detected by LC/MS. **Table S5.4.** Significantly differential metabolites of serum samples from AIA-Hot rats detected by LC/MS.

## Data Availability

All data generated or analyzed during this study are included in this published article and its Additional file.

## Abbreviations

^1^H-NMR: ^1^H-nuclear magnetic resonance
5-HT: 5-Hydroxy-l-typtophan
AIA: Adjuvant-induced arthritis
AUC: Area under the curve
BHGZD: Baihu Guizhi Decoction
CACMS: Chinese Academy of traditional Chinese Medicine
CRP: C-Reactive protein
DAS28-CRP: Disease activity score 28-joint count C-Reactive protein
DAS28-ESR: Disease activity score 28-joint count erythrocyte sedimentation rate
DEGs: Differentially expressed genes
ELISA: Enzyme-linked immunosorbent assay
ESR: Erythrocyte sedimentation rate
FDR: False discovery rate
FLS: Fibroblast-like synoviocytes
GC/MS: Gas chromatography–mass spectrometer
GLNS: Glutaminase
GO: Gene ontology
HMDB: Human Metabolome Database
HPO: Ontology of human phenotype
I3AA: Indole-3 acetic acid
IAld: Indole-3-carbaldehyde
ICD-11: International Classification of Diseases, 11th Revision
IL17F: Interleukin-17F
IL4I1: Interleukin-4-Induced-1
KEGG: Kyoto Encyclopedia of Genes and Genomes
LC/MS: Liquid chromatography–mass spectrometry
nAChRs: Nicotinic acetylcholine receptors
NC: Normal controls
NLRP3: NOD-like receptor protein 3
OA: Osteoarthritis
OPLS-DA: Orthogonal partial least squares discriminant analysis
PCA: Principal component analysis
PLS-DA: Partial least squares-discriminant analysis
RA: Rheumatoid arthritis
RA-Cold: Cold-dampness Syndrome of Rheumatoid arthritis
RA-Hot: Hot-dampness Syndrome of Rheumatoid arthritis
RF: Rheumatoid factor
ROC: Receiver operating characteristic curve
S1P: Sphingosine 1-phosphate
SDs: Standard deviations
TCA: Tricarboxylic acid
TCM: Traditional Chinese medicine
TCMIP: Integrative Pharmacology-based Research Platform of Traditional Chinese Medicine
VIP: Variable importance in the projection
WTD: Wutou Decoction
