# Supplementary material for: Integrative network fusion-based multi-omics study for biomarker identification and patient classification of rheumatoid arthritis
Source: Chin Med. 2023 May 4;18:48. doi: 10.1186/s13020-023-00750-8 (PMC10158004; doi:10.1186/s13020-023-00750-8)
Supplement: Supplementary file 1 — Additional file 1: Table S1. Clinical data and patient characteristics. [file 13020_2023_750_MOESM1_ESM.pdf]

Table S1. Clinical data and patient characteristics

Names	Syndromes	Hospital	Age (years)	Gender	RF (IU/mL)	ESR (mm/h)	CRP (mg/L)	DAS-28 ESR	DAS-28 CRP
Zhang Wencai	Normal Control	Wang Jing Hospital of CACMS	66	Male	/	/	50.93	/	/
Wan Yongcheng	Normal Control	Wang Jing Hospital of CACMS	73	Male	/	/	6.67	/	/
Zhang Decheng	Normal Control	Wang Jing Hospital of CACMS	75	Male	/	/	29.05	/	/
Cao Yanzhou	Normal Control	Wang Jing Hospital of CACMS	N/A	N/A	/	/	/	/	/
Liu Zhi	Normal Control	Wang Jing Hospital of CACMS	71	Male	/	/	7.73	/	/
Wang Xueli	Normal Control	Wang Jing Hospital of CACMS	56	Female	/	/	/	/	/
Changguan Shan	Normal Control	Wang Jing Hospital of CACMS	N/A	N/A	/	/	11.29	/	/
Sun Shufang	Normal Control	Wang Jing Hospital of CACMS	63	Female	/	/	/	/	/
Shan Linqin	Cold-RA	Xi'an Red Cross Hospital	53	Female	100.4	85	25.36	/	/
Zhang Xiuping	Normal Control	Wang Jing Hospital of CACMS	N/A	N/A	/	/	/	/	/
Yang Weikang	Normal Control	Sports Medicine Hospital (Beijing)	19	Male	/	/	/	/	/
Mu Xin	Normal Control	Sports Medicine Hospital (Beijing)	19	Male	/	/	/	/	/
Chen Fangjun	Normal Control	Sports Medicine Hospital (Beijing)	45	Male	/	9	/	/	/
Niu Ruijuan	Normal Control	Sports Medicine Hospital (Beijing)	35	Female	/	3	/	/	/
Hu Tianhao	Normal Control	Sports Medicine Hospital (Beijing)	17	Male	/	7	0	/	/
Liu Jingyu	Normal Control	Sports Medicine Hospital (Beijing)	17	Male	/	/	/	/	/
Li Mengya	Normal Control	Sports Medicine Hospital (Beijing)	16	Female	/	7	/	/	/
Gong Manfang	Cold-RA	Xi'an Red Cross Hospital	60	Female	135.1	98	32.48	/	/
Yu Hongxia	Cold-RA	Wang Jing Hospital of CACMS	60	Female	/	68	11.5	/	/
Li Shurong	Cold-RA	Wang Jing Hospital of CACMS	63	Female	/	12	0.26	/	/
Jin Heying	Cold-RA	Peking University Shougang Hospital	77	Female	/	97	24.67	/	/
Wei Yanping	Cold-RA	Baotou Mongolian TCM Hospital	49	Female	85.8	60	/	/	/
Jin Denglong	Normal Control	Sports Medicine Hospital (Beijing)	48	Male	/	/	1	/	/
Pu Yulan	Normal Control	Wang Jing Hospital of CACMS	73	Female	8.4	20	25.69	/	/
Li Xiaosheng	Hot-RA	Wang Jing Hospital of CACMS	72	Male	68.3	72	28.9	/	/
Lin Liping	Hot-RA	Henan Rheumatism Hospital	50	Female	5.5	104	91.3	/	/
Yu Yanyan	Hot-RA	Henan Rheumatism Hospital	27	Female	146.8	62	38.39	/	/
Zhao Jinglan	Hot-RA	Wang Jing Hospital of CACMS	39	Female	9.0	6	1.2	/	/
Yang Shiying	Hot-RA	Wang Jing Hospital of CACMS	78	Female	17.2	39	12.6	/	/

Yin Mingrui	Hot-RA	Henan Rheumatism Hospital	31	Female	123.52	41	47.22	/	/
Xing Shuili	Hot-RA	Henan Rheumatism Hospital	40	Female	20.21	110	146.82	/	/
Yang Jianju	Hot-RA	Henan Rheumatism Hospital	75	Female	111.46	100	115.74	/	/
Zhao Aiyun	Hot-RA	Henan Rheumatism Hospital	69	Female	27.15	94	53.24	/	/
Zhou Guiying	Hot-RA	Henan Rheumatism Hospital	53	Female	102.90	30	16.720	/	/
Shi Haijian	Hot-RA	Xi'an Red Cross Hospital	59	Female	89.50	70	19.45	/	/
Wang Fan	Normal Control	Sports Medicine Hospital (Beijing)	28	Male	/	14	/	/	/
Wang Guixiang	Hot-RA	Henan Rheumatism Hospital	55	Female	98.1	73	59.87	/	/
Wang Yumei	Cold-RA	Xi'an Red Cross Hospital	74	Female	133.70	119	36.11	/	/
Wu Zhengxian	Hot-RA	General Hospital of Southern Theatre Command	38	Female	/	/	/	/	/
Xu Xiaoling	Hot-RA	General Hospital of Southern Theatre Command	63	Female	193.00	76	90.2	/	/
A3	Hot-RA	Guang'anmen Hospital of CACMS	55	Female	/	/	/	4.66	/
A5	Hot-RA	Guang'anmen Hospital of CACMS	56	Female	/	/	/	4.65	/
A10	Hot-RA	Guang'anmen Hospital of CACMS	54	Female	/	/	/	/	/
Wang Wenyan	Cold-RA	First Teaching Hospital of Tianjin University of TCM	32	Female	98.1	25	6.8	3.61	3.05
Luo Shaoling	Cold-RA	First Teaching Hospital of Tianjin University of TCM	64	Female	8.38	43	<3.11	5.62	/
Liu Sheng	Cold-RA	First Teaching Hospital of Tianjin University of TCM	66	Male	46.1	27	<3.13	4.06	/
Liu Si	Normal Control	Sports Medicine Hospital (Beijing)	34	Female	/	18	/	/	/
NC2	Normal Control	Sports Medicine Hospital (Beijing)	N/A	N/A	/	/	/	/	/
He Fan	Normal Control	Sports Medicine Hospital (Beijing)	42	Male	/	5	/	/	/
Zhang Xiuping	Normal Control	Sports Medicine Hospital (Beijing)	N/A	N/A	/	/	/	/	/
Normal	Normal Control	Sports Medicine Hospital (Beijing)	N/A	N/A	/	/	/	/	/
Guo Jiawei	Normal Control	Sports Medicine Hospital (Beijing)	N/A	N/A	/	2	/	/	/
Liu Heng	Normal Control	Sports Medicine Hospital (Beijing)	23	Male	/	/	/	/	/
Li Kunyuan	Normal Control	Sports Medicine Hospital (Beijing)	18	Female	/	28	/	/	/
Cao Yanfei	Normal Control	Sports Medicine Hospital (Beijing)	18	Male	/	2	/	/	/
Liu Bei	Normal Control	Sports Medicine Hospital (Beijing)	19	Female	/	4	/	/	/
Diao Shoushi	Hot-RA	Henan Rheumatism Hospital	57	Male	134.26	35	14.35	/	/
Feng Jinzhu	Hot-RA	Henan Rheumatism Hospital	18	Female	156.87	32	24.93	/	/
Gao Hongxia	Hot-RA	Henan Rheumatism Hospital	41	Female	137.02	53	48.94	/	/
Hu Min	Hot-RA	Henan Rheumatism Hospital	28	Female	165.81	68	16.89	/	/
Li Ailian	Hot-RA	Henan Rheumatism Hospital	51	Female	156.02	106	141.14	/	/
Liu Bohan	Hot-RA	Henan Rheumatism Hospital	16	Female	126.18	36	81.42	/	/

Lu Xiaowu	Hot-RA	Henan Rheumatism Hospital	51	Female	81	108	24.7	/	/
Qu Xiaohong	Hot-RA	Henan Rheumatism Hospital	47	Female	167.14	41	14.13	/	/
Sun Fuxiang	Hot-RA	Henan Rheumatism Hospital	47	Female	114.78	79	32.92	/	/
Li Erling	Cold-RA	Henan Rheumatism Hospital	46	Female	122.48	42	56.53	/	/
Li Changsuo	Cold-RA	Henan Rheumatism Hospital	N/A	N/A	137.49	54	24.4	/	/
Qi Guiying	Cold-RA	Henan Rheumatism Hospital	N/A	N/A	126.75	93	31.21	/	/
Li Huishen	Cold-RA	Wang Jing Hospital of CACMS	N/A	N/A	/	/	/	/	/
Wei Yanling	Cold-RA	Henan Rheumatism Hospital	37	Female	141.86	84	80.88	/	/
Xu Qiumei	Cold-RA	Henan Rheumatism Hospital	52	Female	131.12	30	28.63	/	/
Zhao Weixun	Cold-RA	Wang Jing Hospital of CACMS	N/A	N/A	/	/	/	/	/
LM	Normal Control	First Teaching Hospital of Tianjin University of TCM	26	Female	/	/	/	/	/
SQH	Normal Control	First Teaching Hospital of Tianjin University of TCM	26	Male	/	/	/	/	/
ZQ	Normal Control	First Teaching Hospital of Tianjin University of TCM	27	Female	/	/	/	/	/
YP	Normal Control	First Teaching Hospital of Tianjin University of TCM	29	Male	/	/	/	/	/
king	Normal Control	Sports Medicine Hospital (Beijing)	N/A	N/A	/	/	/	/	/
20181229	Normal Control	Sports Medicine Hospital (Beijing)	N/A	N/A	/	/	/	/	/
Zhang Chunran	Normal Control	Wang Jing Hospital of CACMS	79	Female	/	/	/	/	/
Ren Na	Normal Control	Sports Medicine Hospital (Beijing)	31	Female	/	6	/	/	/
WY	Normal Control	First Teaching Hospital of Tianjin University of TCM	62	Male	/	/	/	/	/
DK	Normal Control	First Teaching Hospital of Tianjin University of TCM	56	Male	/	/	/	/	/
SO	Normal Control	First Teaching Hospital of Tianjin University of TCM	31	Male	/	/	/	/	/
AH	Normal Control	First Teaching Hospital of Tianjin University of TCM	28	Female	/	/	/	/	/
XP	Normal Control	First Teaching Hospital of Tianjin University of TCM	25	Female	/	/	/	/	/
PH	Normal Control	First Teaching Hospital of Tianjin University of TCM	27	Male	/	/	/	/	/
FCN	Normal Control	First Teaching Hospital of Tianjin University of TCM	44	Female	/	/	/	/	/
XY	Normal Control	First Teaching Hospital of Tianjin University of TCM	39	Female	/	/	/	/	/
JL	Normal Control	First Teaching Hospital of Tianjin University of TCM	23	Male	/	/	/	/	/
YX	Normal Control	First Teaching Hospital of Tianjin University of TCM	23	Female	/	/	/	/	/
Wang Guiyuan	Cold-RA	Gansu Provincial TCM Hospital	58	Female	/	38	2.31	5.98	4.82
Shi Junfen	Cold-RA	Gansu Provincial TCM Hospital	52.5	Female	/	43	13.44	6.17	5.46
Xue Lanhua	Cold-RA	Gansu Provincial TCM Hospital	50	Female	/	65	68.42	6.79	6.35
Marshmai	Cold-RA	Gansu Provincial TCM Hospital	49	Female	/	30	17.86	5.58	5.22
Yan Yuling	Cold-RA	Gansu Provincial TCM Hospital	51	Female	/	83	114.58	6.94	6.52

Wu Guoqiao	Cold-RA	Gansu Provincial TCM Hospital	45	Female	/	8	2.76	3.39	3.37
Wei Ga'e	Cold-RA	Gansu Provincial TCM Hospital	48	Female	/	67	32.15	6.91	6.19
Yong Jianmin	Cold-RA	Gansu Provincial TCM Hospital	50	Male	/	42	9.51	4.86	4.05
Wang Xianxiu	Cold-RA	Gansu Provincial TCM Hospital	48	Female	/	4	2.63	4.58	5.03
Lan Yinhua	Cold-RA	Gansu Provincial TCM Hospital	47	Female	/	20	6.23	6.27	5.84
Zou Yueqin	Cold-RA	Gansu Provincial TCM Hospital	49	Female	/	90	63.31	7.29	6.60
Zhang Yanmei	Cold-RA	Gansu Provincial TCM Hospital	60	Female	/	7	4.5	4.54	4.75
Liu Suping	Cold-RA	Gansu Provincial TCM Hospital	56	Female	/	7	3.84	4.83	4.99
Du Xishun	Cold-RA	Gansu Provincial TCM Hospital	50	Female	/	46	42.16	6.59	6.22
Wang Youju	Cold-RA	Gansu Provincial TCM Hospital	46	Female	/	20	19.03	5.88	5.82
LZW	Cold-RA	First Teaching Hospital of Tianjin University of TCM	26	Female	20	13	2.42	3.82	3.43
BFQ	Cold-RA	First Teaching Hospital of Tianjin University of TCM	67	Female	9.19	26	<3.13	3.82	/
LLD	Cold-RA	First Teaching Hospital of Tianjin University of TCM	58	Male	9.19	11	<3.13	1.82	/
WJX	Cold-RA	First Teaching Hospital of Tianjin University of TCM	38	Female	9.19	3	<3.13	2.69	/
GCJ	Cold-RA	First Teaching Hospital of Tianjin University of TCM	70	Male	567	81	45.4	5.37	4.63
Chen Yanfei	Cold-RA	Henan Rheumatism Hospital	N/A	N/A	18.82	99	141.19		/
XGR	Hot-RA	First Teaching Hospital of Tianjin University of TCM	58	Female	20.6	66	55.3	5.81	5.29
ZQP	Hot-RA	First Teaching Hospital of Tianjin University of TCM	60	Female	24.4	49	3.11	5.99	/
XYR	Hot-RA	First Teaching Hospital of Tianjin University of TCM	62	Female	58.6	43	3.11	4.93	/
HYB	Hot-RA	First Teaching Hospital of Tianjin University of TCM	48	Female	395	28	20.7	6.73	6.47
YJH	Hot-RA	First Teaching Hospital of Tianjin University of TCM	58	Female	9.19	23	3.11	5.58	/
ZRY	Hot-RA	First Teaching Hospital of Tianjin University of TCM	55	Female	112	56	7.91	6.23	5.16
FRJ	Hot-RA	First Teaching Hospital of Tianjin University of TCM	56	Female	64.4	72	25.6	6.86	6.01
WXL	Hot-RA	First Teaching Hospital of Tianjin University of TCM	59	Female	433	45	17.6	5.25	4.60
ZYL	Hot-RA	First Teaching Hospital of Tianjin University of TCM	71	Male	9.19	41	82.4	6.76	6.71
LYZ	Hot-RA	First Teaching Hospital of Tianjin University of TCM	65	Female	13.4	26	6.94	4.70	4.12
ZSJ	Hot-RA	First Teaching Hospital of Tianjin University of TCM	52	Female	171	16	3.13	3.75	/
HSH	Hot-RA	First Teaching Hospital of Tianjin University of TCM	64	Female	20	52	12.2	6.67	5.79
ZXZ	Hot-RA	First Teaching Hospital of Tianjin University of TCM	65	Female	9.19	16	4.38	3.47	3.09
LYQ	Hot-RA	First Teaching Hospital of Tianjin University of TCM	62	Female	11.9	16	3.13	3.59	/
MXY	Hot-RA	First Teaching Hospital of Tianjin University of TCM	74	Female	12.5	74	23.4	6.53	5.63
Zhang Hong	Hot-RA	Henan Rheumatism Hospital	53	Female	14.51	31	19.8	/	/
Yuan Sen	Hot-RA	Henan Rheumatism Hospital	38	Male	/	93	112.38	/	/

Wang Xinmin	Hot-RA	Henan Rheumatism Hospital	66	Male	116.30	60	19.94	/	/
Zhang Haojun	Hot-RA	Henan Rheumatism Hospital	53	Male	30.29	51	80.28	/	/
