# Supplementary material for: Integrative network fusion-based multi-omics study for biomarker identification and patient classification of rheumatoid arthritis
Source: Chin Med. 2023 May 4;18:48. doi: 10.1186/s13020-023-00750-8 (PMC10158004; doi:10.1186/s13020-023-00750-8)
Supplement: Supplementary file 2 — Additional file 2. Pathways enriched by key network targets of RA-Cold and RA-Hot: Table S2.1. WikiPathways enriched by key network targets of RA-Cold. Table S2.2. Reactome Pathways enriched by key network targets of RA-Cold. Table S2.3. KEGG-Pathways enriched by key network targets of RA-Cold. Table S2.4. WikiPathways enriched by key network targets of RA-Hot. Table S2.5. Reactome-Pathways enriched by key network targets of RA-Hot. Table S2.6. KEGG-Pathways enriched by key network targets of RA-Hot. [file 13020_2023_750_MOESM2_ESM.pdf]

Table S2.1. WikiPathways enriched by key network targets of RA-Cold

No	Type	Name	Number of hits	Expected score	Adjusted p-value
1	enriched	Allograft Rejection	25	1.19439	2.11E-020
2	enriched	Viral Acute Myocarditis	22	1.12729	1.93E-017
3	enriched	T-Cell antigen Receptor (TCR) Signaling Pathway	22	1.20781	4.62E-017
4	enriched	Regulation of toll-like receptor signaling pathway	23	1.8654	9.94E-015
5	enriched	Type II interferon signaling (IFNG)	15	0.496544	4.43E-014
6	enriched	T-Cell antigen Receptor (TCR) pathway during Staphylococcus aureus infection	16	0.832047	1.32E-012
7	enriched	Chemokine signaling pathway	22	2.2009	1.46E-012
8	enriched	Toll-like Receptor Signaling Pathway	18	1.38227	7.00E-012
9	enriched	B Cell Receptor Signaling Pathway	17	1.30175	3.13E-011
10	enriched	Interactions between immune cells and microRNAs in tumor microenvironment	11	0.375763	3.63E-010
11	enriched	Selective expression of chemokine receptors during T-cell polarization	11	0.389183	4.50E-010
12	enriched	Non-genomic actions of 1,25 dihydroxyvitamin D3	14	0.952828	8.35E-010
13	enriched	Lung fibrosis	13	0.845467	2.55E-009
14	enriched	PI3K-Akt Signaling Pathway	25	4.56284	2.55E-009
15	enriched	Spinal Cord Injury	16	1.55673	2.55E-009
16	enriched	Cytokines and Inflammatory Response	10	0.348923	2.60E-009
17	enriched	miRNAs involvement in the immune response in sepsis	11	0.496544	2.60E-009
18	enriched	Nucleotide-binding Oligomerization Domain (NOD) pathway	11	0.550225	6.24E-009
19	enriched	Hematopoietic Stem Cell Differentiation	12	0.738106	6.31E-009
20	enriched	Ebola Virus Pathway on Host	16	1.73119	7.86E-009
21	enriched	Hepatitis B infection	17	2.02644	7.86E-009
22	enriched	Cancer immunotherapy by PD-1 blockade	9	0.308663	1.72E-008
23	enriched	Thymic Stromal LymphoPoietin (TSLP) Signaling Pathway	11	0.630745	1.72E-008
24	enriched	Apoptosis	13	1.12729	3.49E-008
25	enriched	Pancreatic adenocarcinoma pathway	13	1.19439	6.34E-008
26	enriched	IL-2 Signaling Pathway	10	0.563645	8.63E-008
27	enriched	Vitamin D Receptor Pathway	17	2.44246	8.63E-008
28	enriched	Inflammatory Response Pathway	9	0.402603	9.84E-008
29	enriched	Regulatory circuits of the STAT3 signaling pathway	12	1.04677	1.44E-007
30	enriched	Osteoblast Signaling	7	0.187882	3.67E-007
31	enriched	IL-7 Signaling Pathway	8	0.335503	4.82E-007
32	enriched	Microglia Pathogen Phagocytosis Pathway	9	0.536804	7.44E-007
33	enriched	Fibrin Complement Receptor 3 Signaling Pathway	9	0.550225	8.70E-007
34	enriched	Breast cancer pathway	14	2.0667	2.74E-006
35	enriched	Adipogenesis	13	1.74461	2.86E-006
36	enriched	DNA Damage Response (only ATM dependent)	12	1.47621	3.79E-006
37	enriched	Control of immune tolerance by vasoactive intestinal peptide	6	0.174461	5.44E-006
38	enriched	PDGF Pathway	8	0.523384	7.58E-006
39	enriched	Leptin signaling pathway	10	1.01993	8.84E-006

40	enriched	Kit receptor signaling pathway	9	0.791786	1.15E-005
41	enriched	DNA IR-damage and cellular response via ATR	10	1.07361	1.30E-005
42	enriched	Interleukin-11 Signaling Pathway	8	0.590485	1.55E-005
43	enriched	Senescence and Autophagy in Cancer	11	1.40911	1.61E-005
44	enriched	Toll-like Receptor Signaling related to MyD88	7	0.416023	2.24E-005
45	enriched	LTF danger signal response pathway	6	0.254982	2.73E-005
46	enriched	DNA Damage Response	9	0.912567	2.88E-005
47	enriched	Hepatitis C and Hepatocellular Carcinoma	8	0.657585	2.88E-005
48	enriched	Resistin as a regulator of inflammation	7	0.442864	2.97E-005
49	enriched	Focal Adhesion	14	2.65718	3.31E-005
50	enriched	miRNA Regulation of DNA Damage Response	9	0.952828	3.76E-005
51	enriched	Brain-Derived Neurotrophic Factor (BDNF) signaling pathway	12	1.9325	3.88E-005
52	enriched	Photodynamic therapy-induced NF-kB survival signaling	7	0.469704	3.88E-005
53	enriched	VEGFA-VEGFR2 Signaling Pathway	15	3.16715	4.41E-005
54	enriched	Cytosolic DNA-sensing pathway	9	0.993088	4.66E-005
55	enriched	IL-4 Signaling Pathway	8	0.724686	4.66E-005
56	enriched	Interferon type I signaling pathways	8	0.724686	4.66E-005
57	enriched	DNA IR-Double Strand Breaks (DSBs) and cellular response via ATM	8	0.738106	5.09E-005
58	enriched	RANKL/RANK (Receptor activator of NFKB (ligand)) Signaling Pathway	8	0.738106	5.09E-005
59	enriched	The human immune response to tuberculosis	6	0.308663	5.35E-005
60	enriched	Integrated Breast Cancer Pathway	12	2.03986	5.60E-005
61	enriched	IL1 and megakaryocytes in obesity	6	0.322083	6.39E-005
62	enriched	IL-5 Signaling Pathway	7	0.536804	7.16E-005
63	enriched	Signaling Pathways in Glioblastoma	9	1.10045	8.88E-005
64	enriched	EPO Receptor Signaling	6	0.348923	9.03E-005
65	enriched	Fas Ligand (FasL) pathway and Stress induction of Heat Shock Proteins (HSP) regulation	7	0.563645	9.03E-005
66	enriched	Notch Signaling Pathway Netpath	8	0.818627	9.03E-005
67	enriched	Cancer immunotherapy by CTLA4 blockade	5	0.187882	9.84E-005
68	enriched	MicroRNAs in cardiomyocyte hypertrophy	9	1.12729	9.87E-005
69	enriched	IL-6 signaling pathway	7	0.577065	9.88E-005
70	enriched	TLR4 Signaling and Tolerance	6	0.375763	1.21E-004
71	enriched	ATM Signaling Network in Development and Disease	7	0.603905	1.26E-004
72	enriched	Oncostatin M Signaling Pathway	8	0.872307	1.27E-004
73	enriched	AGE/RAGE pathway	8	0.885727	1.33E-004
74	enriched	Focal Adhesion-PI3K-Akt-mTOR-signaling pathway	16	4.06629	1.33E-004
75	enriched	PDGFR-beta pathway	6	0.389183	1.33E-004
76	enriched	T-Cell Receptor and Co-stimulatory Signaling	6	0.389183	1.33E-004
77	enriched	Osteoclast Signaling	5	0.214722	1.47E-004
78	enriched	RAC1/PAK1/p38/MMP2 Pathway	8	0.912567	1.59E-004
79	enriched	Differentiation Pathway	7	0.644165	1.66E-004
80	enriched	Platelet-mediated interactions with vascular and circulating cells	5	0.228142	1.81E-004
81	enriched	IL-3 Signaling Pathway	7	0.657585	1.83E-004

82	enriched	Hippo-Merlin Signaling Dysregulation	10	1.61041	1.90E-004
83	enriched	Development and heterogeneity of the ILC family	6	0.429444	1.96E-004
84	enriched	Mammary gland development pathway - Pregnancy and lactation (Stage 3 of 4)	6	0.429444	1.96E-004
85	enriched	Photodynamic therapy-induced AP-1 survival signaling.	7	0.671005	1.96E-004
86	enriched	Small cell lung cancer	9	1.27491	1.96E-004
87	enriched	MAPK Signaling Pathway	14	3.30135	2.00E-004
88	enriched	Simplified Depiction of MYD88 Distinct Input-Output Pathway	5	0.241562	2.08E-004
89	enriched	Signal transduction through IL1R	6	0.442864	2.18E-004
90	enriched	Prolactin Signaling Pathway	8	1.01993	2.89E-004
91	enriched	TGF-beta Receptor Signaling	7	0.724686	2.89E-004
92	enriched	IL-1 signaling pathway	7	0.738106	3.19E-004
93	enriched	EGF/EGFR Signaling Pathway	11	2.16064	3.48E-004
94	enriched	Role of Altered Glycolysation of MUC1 in Tumour Microenvironment	4	0.120781	3.50E-004
95	enriched	PI3K/AKT/mTOR - VitD3 Signalling	5	0.295242	4.37E-004
96	enriched	Vitamin D in inflammatory diseases	5	0.295242	4.37E-004
97	enriched	Gastrin Signaling Pathway	9	1.52989	6.64E-004
98	enriched	Hair Follicle Development: Cytodifferentiation (Part 3 of 3)	8	1.16755	6.64E-004
99	enriched	Genes involved in male infertility	10	1.95934	7.66E-004
100	enriched	Apoptosis Modulation and Signaling	8	1.20781	8.04E-004
101	enriched	IL-10 Anti-inflammatory Signaling Pathway	4	0.161041	8.04E-004
102	enriched	Head and Neck Squamous Cell Carcinoma	7	0.885727	8.18E-004
103	enriched	Human Thyroid Stimulating Hormone (TSH) signaling pathway	7	0.885727	8.18E-004
104	enriched	Integrated Cancer Pathway	6	0.590485	8.18E-004
105	enriched	Wnt/beta-catenin Signaling Pathway in Leukemia	5	0.348923	8.18E-004
106	enriched	Ras Signaling	11	2.4693	9.66E-004
107	enriched	Serotonin Receptor 2 and STAT3 Signaling	3	0.0536804	1.21E-003
108	enriched	Human Complement System	8	1.30175	1.22E-003
109	enriched	Sudden Infant Death Syndrome (SIDS) Susceptibility Pathways	10	2.10696	1.24E-003
110	enriched	Non-small cell lung cancer	7	0.966248	1.29E-003
111	enriched	Primary Focal Segmental Glomerulosclerosis FSGS	7	0.966248	1.29E-003
112	enriched	Chromosomal and microsatellite instability in colorectal cancer	7	0.979668	1.38E-003
113	enriched	Mammary gland development pathway - Embryonic development (Stage 1 of 4)	4	0.201302	1.46E-003
114	enriched	Peptide GPCRs	7	0.993088	1.46E-003
115	enriched	miRNAs involved in DNA damage response	4	0.201302	1.46E-003
116	enriched	Wnt Signaling Pathway (Netpath)	6	0.697846	1.68E-003
117	enriched	IL17 signaling pathway	5	0.429444	1.72E-003
118	enriched	Monoamine Transport	5	0.429444	1.72E-003
119	enriched	miRNA regulation of prostate cancer signaling pathways	5	0.442864	1.95E-003
120	enriched	Cells and Molecules involved in local acute inflammatory response	4	0.228142	2.09E-003
121	enriched	IL-9 Signaling Pathway	4	0.228142	2.09E-003
122	enriched	Nuclear Receptors Meta-Pathway	14	4.28102	2.10E-003

123	enriched	Alzheimers Disease	7	1.10045	2.47E-003
124	enriched	Overview of nanoparticle effects	4	0.254982	2.94E-003
125	enriched	TP53 Network	4	0.254982	2.94E-003
126	enriched	Mesodermal Commitment Pathway	9	1.97276	3.20E-003
127	enriched	TYROBP Causal Network	6	0.818627	3.43E-003
128	enriched	ATM Signaling Pathway	5	0.536804	4.02E-003
129	enriched	Androgen receptor signaling pathway	7	1.20781	4.02E-003
130	enriched	Cell Cycle	8	1.61041	4.02E-003
131	enriched	Corticotropin-releasing hormone signaling pathway	7	1.23465	4.44E-003
132	enriched	TNF alpha Signaling Pathway	7	1.23465	4.44E-003
133	enriched	Estrogen signaling pathway	4	0.308663	5.25E-003
134	enriched	Cytosine methylation	3	0.120781	5.80E-003
135	enriched	miRNA regulation of p53 pathway in prostate cancer	4	0.322083	5.97E-003
136	enriched	Pathways Regulating Hippo Signaling	7	1.31517	6.16E-003
137	enriched	Aryl Hydrocarbon Receptor Pathway	5	0.617325	6.83E-003
138	enriched	Regulation of Microtubule Cytoskeleton	5	0.617325	6.83E-003
139	enriched	Canonical and Non-canonical Notch signaling	4	0.362343	8.64E-003
140	enriched	Cardiac Progenitor Differentiation	5	0.711266	1.21E-002
141	enriched	Dopaminergic Neurogenesis	4	0.402603	1.21E-002
142	enriched	Oligodendrocyte Specification and differentiation(including remyelination), leading to Myelin Components for CNS	4	0.402603	1.21E-002
143	enriched	Cell-type Dependent Selectivity of CCK2R Signaling	3	0.174461	1.31E-002
144	enriched	Development of pulmonary dendritic cells and macrophage subsets	3	0.174461	1.31E-002
145	enriched	ncRNAs involved in STAT3 signaling in hepatocellular carcinoma	3	0.174461	1.31E-002
146	enriched	ESC Pluripotency Pathways	7	1.55673	1.47E-002
147	enriched	Pathways in clear cell renal cell carcinoma	6	1.14071	1.52E-002
148	enriched	Retinoblastoma Gene in Cancer	6	1.16755	1.69E-002
149	enriched	Role of Osx and miRNAs in tooth development	3	0.201302	1.83E-002
150	enriched	Novel intracellular components of RIG-I-like receptor (RLR) pathway	5	0.805207	1.90E-002
151	enriched	Overview of leukocyte-intrinsic Hippo pathway functions	4	0.469704	1.91E-002
152	enriched	Epithelial to mesenchymal transition in colorectal cancer	8	2.1338	1.95E-002
153	enriched	ErbB Signaling Pathway	6	1.22123	2.03E-002
154	enriched	Overview of interferons-mediated signaling pathway	4	0.483124	2.06E-002
155	enriched	Amyotrophic lateral sclerosis (ALS)	4	0.496544	2.22E-002
156	enriched	Endometrial cancer	5	0.845467	2.22E-002
157	enriched	Neovascularisation processes	4	0.496544	2.22E-002
158	enriched	LncRNA involvement in canonical Wnt signaling and colorectal cancer	6	1.26149	2.29E-002
159	enriched	Amplification and Expansion of Oncogenic Pathways as Metastatic Traits	3	0.228142	2.30E-002
160	enriched	Endochondral Ossification	5	0.858887	2.30E-002
161	enriched	G1 to S cell cycle control	5	0.858887	2.30E-002
162	enriched	Leptin Insulin Overlap	3	0.228142	2.30E-002

163	enriched	Pathways Affected in Adenoid Cystic Carcinoma	5	0.872307	2.43E-002
164	enriched	MicroRNA network associated with chronic lymphocytic leukemia	2	0.0536804	2.58E-002
165	enriched	Oxytocin signaling	2	0.0536804	2.58E-002
166	enriched	TGF-beta Signaling Pathway	7	1.77145	2.59E-002
167	enriched	Bladder Cancer	4	0.536804	2.71E-002
168	enriched	Extracellular vesicles in the crosstalk of cardiac cells	3	0.254982	2.94E-002
169	enriched	Integrin-mediated Cell Adhesion	6	1.35543	3.01E-002
170	enriched	TNF related weak inducer of apoptosis (TWEAK) Signaling Pathway	4	0.563645	3.12E-002
171	enriched	Wnt Signaling Pathway and Pluripotency	6	1.36885	3.12E-002
172	enriched	Hematopoietic Stem Cell Gene Regulation by GABP alpha/beta Complex	3	0.268402	3.25E-002
173	enriched	Imatinib and Chronic Myeloid Leukemia	3	0.268402	3.25E-002
174	enriched	Endoderm Differentiation	7	1.89224	3.50E-002
175	enriched	NO/cGMP/PKG mediated Neuroprotection	4	0.630745	4.43E-002
176	enriched	Metastatic brain tumor	2	0.0805207	4.44E-002
177	enriched	EBV LMP1 signaling	3	0.308663	4.53E-002
178	enriched	Rett syndrome causing genes	4	0.644165	4.67E-002
179	enriched	Regulation of Actin Cytoskeleton	7	2.01302	4.70E-002

Table S2.2. Reactome Pathways enriched by key network targets of RA-Cold

No	Type	Name	Number of hits	Expected score	Adjusted p-value
1	enriched	Interferon gamma signaling	20	1.15413	1.15E-014
2	enriched	Interleukin-10 signaling	17	0.630745	1.15E-014
3	enriched	Interleukin-4 and Interleukin-13 signaling	21	1.44937	2.74E-014
4	enriched	Chemokine receptors bind chemokines	17	0.791786	8.17E-014
5	enriched	Generation of second messenger molecules	12	0.442864	2.15E-010
6	enriched	Phosphorylation of CD3 and TCR zeta chains	10	0.295242	2.83E-009
7	enriched	PD-1 signaling	10	0.308663	3.43E-009
8	enriched	Downstream TCR signaling	15	1.31517	7.84E-009
9	enriched	Translocation of ZAP-70 to Immunological synapse	9	0.254982	1.63E-008
10	enriched	Immunoregulatory interactions between a Lymphoid and a non-Lymphoid cell	15	1.77145	2.74E-007
11	enriched	Neutrophil degranulation	26	6.44165	9.09E-007
12	enriched	ER-Phagosome pathway	12	1.11387	9.46E-007
13	enriched	Interferon alpha/beta signaling	11	0.899147	1.11E-006
14	enriched	Interleukin-7 signaling	9	0.483124	1.11E-006
15	enriched	Antigen activates B Cell Receptor (BCR) leading to generation of second messengers	8	0.429444	7.33E-006
16	enriched	Cell surface interactions at the vascular wall	11	1.18097	1.22E-005
17	enriched	RHO GTPases Activate NADPH Oxidases	7	0.322083	1.89E-005
18	enriched	G alpha (i) signalling events	17	3.51607	2.79E-005
19	enriched	DAP12 signaling	7	0.402603	6.17E-005
20	enriched	Interleukin-23 signaling	5	0.120781	8.44E-005
21	enriched	MyD88:MAL(TIRAP) cascade initiated on plasma membrane	6	0.254982	8.44E-005
22	enriched	TNFR2 non-canonical NF-kB pathway	9	0.925988	9.91E-005
23	enriched	MyD88 deficiency (TLR2/4)	5	0.134201	1.11E-004
24	enriched	PIP3 activates AKT signaling	10	1.24807	1.13E-004

25	enriched	GPVI-mediated activation cascade	7	0.469704	1.16E-004
26	enriched	IRAK4 deficiency (TLR2/4)	5	0.147621	1.37E-004
27	enriched	Interleukin-6 signaling	5	0.147621	1.37E-004
28	enriched	Constitutive Signaling by Aberrant PI3K in Cancer	9	1.00651	1.47E-004
29	enriched	Interleukin-35 Signalling	5	0.161041	1.78E-004
30	enriched	PI5P, PP2A and IER3 Regulate PI3K/AKT Signaling	10	1.38227	2.12E-004
31	enriched	Dual Incision in GG-NER	7	0.550225	2.39E-004
32	enriched	Regulation of IFNG signaling	5	0.187882	2.97E-004
33	enriched	Formation of Incision Complex in GG-NER	7	0.577065	2.98E-004
34	enriched	Downstream signal transduction	6	0.389183	4.27E-004
35	enriched	TNFR1-induced NFkappaB signaling pathway	6	0.402603	4.93E-004
36	enriched	TNF receptor superfamily (TNFSF) members mediating non-canonical NF-kB pathway	5	0.241562	7.31E-004
37	enriched	Regulation of TNFR1 signaling	6	0.442864	7.55E-004
38	enriched	Cross-presentation of particulate exogenous antigens (phagosomes)	4	0.107361	8.51E-004
39	enriched	ROS and RNS production in phagocytes	6	0.456284	8.51E-004
40	enriched	NOD1/2 Signaling Pathway	6	0.483124	1.09E-003
41	enriched	RUNX3 regulates p14-ARF	4	0.120781	1.16E-003
42	enriched	Signaling by SCF-KIT	6	0.496544	1.19E-003
43	enriched	Interleukin-12 signaling	4	0.134201	1.49E-003
44	enriched	Interleukin-21 signaling	4	0.134201	1.49E-003
45	enriched	Growth hormone receptor signaling	5	0.322083	1.93E-003
46	enriched	Interleukin-1 signaling	8	1.18097	2.18E-003
47	enriched	Interleukin-20 family signaling	5	0.335503	2.20E-003
48	enriched	Interleukin-2 signaling	4	0.161041	2.39E-003
49	enriched	TP53 Regulates Transcription of Caspase Activators and Caspases	4	0.161041	2.39E-003
50	enriched	Activation of NF-kappaB in B cells	7	0.899147	2.70E-003
51	enriched	Interleukin receptor SHC signaling	5	0.362343	2.80E-003
52	enriched	MHC class II antigen presentation	9	1.65067	3.08E-003
53	enriched	Nonhomologous End-Joining (NHEJ)	7	0.925988	3.08E-003
54	enriched	TNFs bind their physiological receptors	5	0.389183	3.58E-003
55	enriched	The NLRP3 inflammasome	4	0.201302	4.39E-003
56	enriched	Recruitment and ATM-mediated phosphorylation of repair and signaling proteins at DNA double strand breaks	7	1.01993	5.03E-003
57	enriched	Signal regulatory protein family interactions	4	0.214722	5.24E-003
58	enriched	CLEC7A (Dectin-1) signaling	7	1.07361	6.54E-003
59	enriched	FCERI mediated NF-kB activation	7	1.10045	7.13E-003
60	enriched	RUNX3 Regulates Immune Response and Cell Migration	3	0.0805207	7.13E-003
61	enriched	Signaling by cytosolic FGFR1 fusion mutants	4	0.241562	7.13E-003
62	enriched	Telomere Extension By Telomerase	3	0.0805207	7.13E-003
63	enriched	G2/M DNA damage checkpoint	7	1.12729	7.97E-003
64	enriched	Regulation of TLR by endogenous ligand	4	0.254982	8.27E-003
65	enriched	Ovarian tumor domain proteases	5	0.509964	9.33E-003
66	enriched	RUNX3 regulates CDKN1A transcription	3	0.0939408	9.36E-003
67	enriched	Regulation of actin dynamics for phagocytic cup formation	6	0.818627	9.36E-003
68	enriched	VEGFA-VEGFR2 Pathway	6	0.832047	1.00E-002
69	enriched	RUNX3 regulates WNT signaling	3	0.107361	1.23E-002

70	enriched	Pre-NOTCH Transcription and Translation	7	1.24807	1.28E-002
71	enriched	DNA Damage/Telomere Stress Induced Senescence	6	0.885727	1.29E-002
72	enriched	Dual incision in TC-NER	6	0.885727	1.29E-002
73	enriched	RAF/MAP kinase cascade	8	1.67751	1.33E-002
74	enriched	Interleukin-9 signaling	3	0.120781	1.48E-002
75	enriched	MAPK1 (ERK2) activation	3	0.120781	1.48E-002
76	enriched	OAS antiviral response	3	0.120781	1.48E-002
77	enriched	RUNX1 regulates transcription of genes involved in differentiation of HSCs	8	1.74461	1.62E-002
78	enriched	Antigen Presentation: Folding, assembly and peptide loading of class I MHC	4	0.335503	1.69E-002
79	enriched	IKK complex recruitment mediated by RIP1	4	0.335503	1.69E-002
80	enriched	MAPK3 (ERK1) activation	3	0.134201	1.78E-002
81	enriched	RUNX1 and FOXP3 control the development of regulatory T lymphocytes (Tregs)	3	0.134201	1.78E-002
82	enriched	Resolution of D-loop Structures through Synthesis-Dependent Strand Annealing (SDSA)	4	0.348923	1.86E-002
83	enriched	Syndecan interactions	4	0.362343	2.09E-002
84	enriched	Interleukin-27 signaling	3	0.147621	2.11E-002
85	enriched	Signaling by Leptin	3	0.147621	2.11E-002
86	enriched	Stabilization of p53	3	0.147621	2.11E-002
87	enriched	CD28 dependent Vav1 pathway	3	0.161041	2.56E-002
88	enriched	PECAM1 interactions	3	0.161041	2.56E-002
89	enriched	Regulation of TP53 Expression	2	0.0268402	2.77E-002
90	enriched	The IPAF inflammasome	2	0.0268402	2.77E-002
91	enriched	Integrin cell surface interactions	6	1.14071	3.54E-002
92	enriched	Interleukin-15 signaling	3	0.187882	3.59E-002
93	enriched	FCERI mediated Ca+2 mobilization	4	0.442864	3.67E-002
94	enriched	IKBKB deficiency causes SCID	2	0.0402603	4.25E-002
95	enriched	IKBKG deficiency causes anhidrotic ectodermal dysplasia with immunodeficiency (EDA-ID) (via TLR)	2	0.0402603	4.25E-002
96	enriched	Signalling to STAT3	2	0.0402603	4.25E-002
97	enriched	The AIM2 inflammasome	2	0.0402603	4.25E-002
98	enriched	Transcriptional regulation of granulopoiesis	6	1.20781	4.32E-002
99	enriched	RIPK1-mediated regulated necrosis	3	0.214722	4.61E-002
100	enriched	Regulation of KIT signaling	3	0.214722	4.61E-002
101	enriched	Detoxification of Reactive Oxygen Species	4	0.496544	4.92E-002
102	enriched	HDR through Single Strand Annealing (SSA)	4	0.496544	4.92E-002

Table S2.3. KEGG-Pathways enriched by key network targets of RA-Cold

No	Type	Name	Number of hits	Expected score	Adjusted p-value
1	enriched	Cytokine-cytokine receptor interaction	42	3.95893	1.59E-025
2	enriched	Epstein-Barr virus infection	36	2.68402	6.11E-025
3	enriched	Leishmaniasis	25	0.993088	1.16E-022
4	enriched	Tuberculosis	32	2.37536	2.72E-022
5	enriched	Toxoplasmosis	27	1.50305	1.60E-021
6	enriched	Inflammatory bowel disease (IBD)	23	0.872307	2.11E-021
7	enriched	Measles	28	1.85198	9.63E-021
8	enriched	Pathways in cancer	45	7.09924	6.06E-020
9	enriched	Influenza A	29	2.268	7.68E-020

10	enriched	Hematopoietic cell lineage	24	1.28833	1.34E-019
11	enriched	Osteoclast differentiation	26	1.69093	1.57E-019
12	enriched	Viral protein interaction with cytokine and cytokine receptor	24	1.34201	2.54E-019
13	enriched	NF-kappa B signaling pathway	24	1.36885	3.50E-019
14	enriched	Primary immunodeficiency	18	0.483124	5.09E-019
15	enriched	Rheumatoid arthritis	23	1.23465	6.43E-019
16	enriched	NOD-like receptor signaling pathway	28	2.41562	2.39E-018
17	enriched	Chemokine signaling pathway	28	2.5364	7.20E-018
18	enriched	Th1 and Th2 cell differentiation	22	1.23465	8.79E-018
19	enriched	Th17 cell differentiation	23	1.43595	9.55E-018
20	enriched	Human T-cell leukemia virus 1 infection	28	2.939	2.04E-016
21	enriched	C-type lectin receptor signaling pathway	21	1.39569	1.05E-015
22	enriched	Toll-like receptor signaling pathway	21	1.40911	1.19E-015
23	enriched	Jak-STAT signaling pathway	24	2.17406	2.48E-015
24	enriched	Human cytomegalovirus infection	27	3.01952	2.98E-015
25	enriched	Allograft rejection	15	0.496544	6.73E-015
26	enriched	Chagas disease (American trypanosomiasis)	20	1.36885	8.36E-015
27	enriched	Intestinal immune network for IgA	15	0.630745	1.15E-013
28	enriched	Type I diabetes mellitus	14	0.577065	7.66E-013
29	enriched	Herpes simplex virus 1 infection	34	6.57585	8.52E-013
30	enriched	T cell receptor signaling pathway	18	1.38227	1.42E-012
31	enriched	Human immunodeficiency virus 1 infection	23	2.83164	3.33E-012
32	enriched	Phagosome	20	2.01302	4.77E-012
33	enriched	Graft-versus-host disease	13	0.550225	7.61E-012
34	enriched	Yersinia infection	18	1.61041	1.30E-011
35	enriched	Viral myocarditis	14	0.791786	2.44E-011
36	enriched	PD-L1 expression and PD-1 checkpoint pathway in cancer	16	1.20781	2.57E-011
37	enriched	B cell receptor signaling pathway	15	1.07361	6.75E-011
38	enriched	Autoimmune thyroid disease	13	0.697846	8.60E-011
39	enriched	Amoebiasis	16	1.34201	9.95E-011
40	enriched	Hepatitis B	19	2.17406	1.25E-010
41	enriched	Natural killer cell mediated cytotoxicity	17	1.74461	3.58E-010
42	enriched	African trypanosomiasis	11	0.483124	6.22E-010
43	enriched	Malaria	12	0.657585	6.61E-010
44	enriched	PI3K-Akt signaling pathway	25	4.7373	1.31E-009
45	enriched	Cell adhesion molecules (CAMs)	17	1.94592	1.56E-009
46	enriched	Asthma	10	0.402603	2.33E-009
47	enriched	Legionellosis	12	0.751526	2.33E-009
48	enriched	Proteoglycans in cancer	19	2.69744	3.21E-009
49	enriched	IL-17 signaling pathway	14	1.26149	4.36E-009
50	enriched	Apoptosis	16	1.81171	4.53E-009
51	enriched	Antigen processing and presentation	13	1.03335	4.78E-009
52	enriched	Pertussis	13	1.04677	5.41E-009
53	enriched	Kaposi sarcoma-associated herpesvirus infection	18	2.49614	6.39E-009
54	enriched	Necroptosis	17	2.18748	6.91E-009
55	enriched	AGE-RAGE signaling pathway in diabetic complications	14	1.34201	8.10E-009
56	enriched	Systemic lupus erythematosus	15	1.78487	2.84E-008
57	enriched	TNF signaling pathway	14	1.50305	2.99E-008
58	enriched	Pancreatic cancer	12	1.00651	3.68E-008
59	enriched	Chronic myeloid leukemia	12	1.01993	4.05E-008
60	enriched	Human papillomavirus infection	22	4.41522	4.05E-008

61	enriched	Staphylococcus aureus infection	13	1.27491	4.05E-008
62	enriched	Hepatitis C	15	2.08012	1.75E-007
63	enriched	MicroRNAs in cancer	15	2.16064	2.75E-007
64	enriched	Fluid shear stress and atherosclerosis	14	1.85198	3.09E-007
65	enriched	Leukocyte transendothelial migration	12	1.50305	1.91E-006
66	enriched	Prolactin signaling pathway	10	0.939408	1.98E-006
67	enriched	Pathogenic Escherichia coli infection	15	2.69744	3.92E-006
68	enriched	Breast cancer	13	1.97276	4.20E-006
69	enriched	Platelet activation	12	1.66409	4.98E-006
70	enriched	Salmonella infection	10	1.07361	5.77E-006
71	enriched	Cytosolic DNA-sensing pathway	9	0.845467	7.88E-006
72	enriched	Parathyroid hormone synthesis, secretion and action	11	1.42253	7.88E-006
73	enriched	Transcriptional misregulation in cancer	14	2.48272	7.88E-006
74	enriched	FoxO signaling pathway	12	1.75803	8.02E-006
75	enriched	Nucleotide excision repair	8	0.630745	1.03E-005
76	enriched	MAPK signaling pathway	17	3.94551	1.59E-005
77	enriched	Focal adhesion	14	2.6706	1.68E-005
78	enriched	Small cell lung cancer	10	1.23465	1.68E-005
79	enriched	Gastric cancer	12	1.97276	2.34E-005
80	enriched	Prostate cancer	10	1.30175	2.55E-005
81	enriched	Rap1 signaling pathway	14	2.81822	2.93E-005
82	enriched	Cellular senescence	12	2.14722	5.13E-005
83	enriched	HIF-1 signaling pathway	10	1.46279	6.47E-005
84	enriched	Fc gamma R-mediated phagocytosis	9	1.23465	1.20E-004
85	enriched	Melanoma	8	0.966248	1.61E-004
86	enriched	p53 signaling pathway	8	0.966248	1.61E-004
87	enriched	Platinum drug resistance	8	0.979668	1.75E-004
88	enriched	Glioma	8	1.00651	2.07E-004
89	enriched	EGFR tyrosine kinase inhibitor resistance	8	1.06019	2.90E-004
90	enriched	Ras signaling pathway	13	3.10005	3.41E-004
91	enriched	Hepatocellular carcinoma	11	2.24116	3.72E-004
92	enriched	Colorectal cancer	8	1.15413	4.93E-004
93	enriched	Non-small cell lung cancer	7	0.885727	7.02E-004
94	enriched	Growth hormone synthesis, secretion and action	9	1.59699	7.12E-004
95	enriched	Thyroid hormone signaling pathway	9	1.59699	7.12E-004
96	enriched	Acute myeloid leukemia	7	0.899147	7.43E-004
97	enriched	Adipocytokine signaling pathway	7	0.925988	8.73E-004
98	enriched	RIG-I-like receptor signaling pathway	7	0.939408	9.40E-004
99	enriched	Endocrine resistance	8	1.31517	1.08E-003
100	enriched	Antifolate resistance	5	0.416023	1.35E-003
101	enriched	Endocrine and other factor-regulated calcium reabsorption	6	0.711266	1.62E-003
102	enriched	Thyroid cancer	5	0.496544	2.80E-003
103	enriched	Sphingolipid signaling pathway	**8**	**1.59699**	3.65E-003
104	enriched	Cell cycle	8	1.66409	4.69E-003
105	enriched	Fc epsilon RI signaling pathway	6	0.899147	5.03E-003
106	enriched	Choline metabolism in cancer	7	1.31517	6.01E-003
107	enriched	Viral carcinogenesis	10	2.69744	6.34E-003
108	enriched	Inflammatory mediator regulation of TRP channels	7	1.34201	6.61E-003
109	enriched	Amyotrophic lateral sclerosis (ALS)	5	0.671005	9.33E-003
110	enriched	Regulation of actin cytoskeleton	10	2.85848	9.45E-003
111	enriched	Cholinergic synapse	7	1.51647	1.27E-002

112	enriched	Non-homologous end-joining	3	0.174461	1.29E-002
113	enriched	Endometrial cancer	5	0.778366	1.65E-002
114	enriched	Neurotrophin signaling pathway	7	1.59699	1.65E-002
115	enriched	Calcium signaling pathway	9	2.59008	1.71E-002
116	enriched	Cushing syndrome	8	2.09354	1.72E-002
117	enriched	Gap junction	6	1.18097	1.72E-002
118	enriched	Hypertrophic cardiomyopathy (HCM)	6	1.20781	1.89E-002
119	enriched	Long-term depression	5	0.818627	1.95E-002
120	enriched	Wnt signaling pathway	8	2.14722	1.95E-002
121	enriched	Basal cell carcinoma	5	0.845467	2.19E-002
122	enriched	Dilated cardiomyopathy (DCM)	6	1.27491	2.37E-002
123	enriched	Central carbon metabolism in cancer	5	0.925988	3.09E-002
124	enriched	Melanogenesis	6	1.35543	3.09E-002
125	enriched	Renal cell carcinoma	5	0.925988	3.09E-002
126	enriched	Epithelial cell signaling in Helicobacter pylori infection	5	0.939408	3.23E-002
127	enriched	Signaling pathways regulating pluripotency of stem cells	7	1.87882	3.52E-002
128	enriched	Insulin resistance	6	1.44937	4.13E-002
129	enriched	Type II diabetes mellitus	4	0.617325	4.26E-002
130	enriched	Phospholipase D signaling pathway	7	1.97276	4.43E-002

Table S2.4. WikiPathways enriched by key network targets of RA-Hot

No	Type	Name	Number of hits	Expected score	Adjusted p-value
1	enriched	PI3K-Akt Signaling Pathway	50	8.21654	1.61E-019
2	enriched	T-Cell antigen Receptor (TCR) Signaling Pathway	30	2.17497	1.61E-019
3	enriched	Allograft Rejection	28	2.1508	1.03E-017
4	enriched	Viral Acute Myocarditis	27	2.02997	2.39E-017
5	enriched	Sudden Infant Death Syndrome (SIDS) Susceptibility Pathways	32	3.79411	7.22E-016
6	enriched	T-Cell antigen Receptor (TCR) pathway during Staphylococcus aureus infection	21	1.49831	1.00E-013
7	enriched	Focal Adhesion-PI3K-Akt-mTOR-signaling pathway	37	7.32239	3.05E-012
8	enriched	Regulation of toll-like receptor signaling pathway	26	3.35911	4.61E-012
9	enriched	Spinal Cord Injury	24	2.80329	6.15E-012
10	enriched	B Cell Receptor Signaling Pathway	22	2.34413	1.33E-011
11	enriched	Toll-like Receptor Signaling Pathway	22	2.48913	3.48E-011
12	enriched	Hepatitis B infection	25	3.64911	1.06E-010
13	enriched	Type II interferon signaling (IFNG)	15	0.894153	1.06E-010
14	enriched	Chemokine signaling pathway	25	3.96327	4.87E-010
15	enriched	Interactions between immune cells and microRNAs in tumor microenvironment	13	0.676656	8.33E-010
16	enriched	Pancreatic adenocarcinoma pathway	19	2.1508	1.03E-009
17	enriched	miRNAs involvement in the immune response in sepsis	14	0.894153	1.03E-009
18	enriched	Focal Adhesion	26	4.78493	2.84E-009
19	enriched	Apoptosis	18	2.02997	3.05E-009
20	enriched	IL-7 Signaling Pathway	12	0.604157	3.05E-009
21	enriched	Cytokines and Inflammatory Response	12	0.628323	4.15E-009

22	enriched	Brain-Derived Neurotrophic Factor (BDNF) signaling pathway	22	3.47995	6.08E-009
23	enriched	Hematopoietic Stem Cell Differentiation	15	1.32915	6.50E-009
24	enriched	Regulatory circuits of the STAT3 signaling pathway	17	1.88497	6.99E-009
25	enriched	Signaling Pathways in Glioblastoma	17	1.98164	1.32E-008
26	enriched	Small cell lung cancer	18	2.2958	1.33E-008
27	enriched	Cancer immunotherapy by PD-1 blockade	11	0.555825	1.58E-008
28	enriched	IL-2 Signaling Pathway	13	1.01498	2.72E-008
29	enriched	Selective expression of chemokine receptors during T-cell polarization	11	0.700822	1.05E-007
30	enriched	MAPK Signaling Pathway	26	5.94491	1.23E-007
31	enriched	Ebola Virus Pathway on Host	19	3.11745	1.52E-007
32	enriched	Fibrin Complement Receptor 3 Signaling Pathway	12	0.990818	1.89E-007
33	enriched	Nucleotide-binding Oligomerization Domain (NOD) pathway	12	0.990818	1.89E-007
34	enriched	Leptin signaling pathway	15	1.83664	2.12E-007
35	enriched	Ras Signaling	22	4.4466	2.48E-007
36	enriched	Photodynamic therapy-induced NF-kB survival signaling	11	0.84582	4.29E-007
37	enriched	Thymic Stromal LymphoPoietin (TSLP) Signaling Pathway	12	1.13582	6.22E-007
38	enriched	Gastrin Signaling Pathway	17	2.75496	7.32E-007
39	enriched	Non-small cell lung cancer	14	1.73997	7.32E-007
40	enriched	VEGFA-VEGFR2 Signaling Pathway	24	5.70324	7.76E-007
41	enriched	Cytosolic DNA-sensing pathway	14	1.78831	9.45E-007
42	enriched	Photodynamic therapy-induced AP-1 survival signaling.	12	1.20831	9.91E-007
43	enriched	Inflammatory Response Pathway	10	0.724989	1.10E-006
44	enriched	Bladder Cancer	11	0.966652	1.13E-006
45	enriched	Lung fibrosis	13	1.52248	1.13E-006
46	enriched	Hippo-Merlin Signaling Dysregulation	17	2.89995	1.21E-006
47	enriched	Senescence and Autophagy in Cancer	16	2.53746	1.22E-006
48	enriched	ErbB Signaling Pathway	15	2.19913	1.29E-006
49	enriched	Oncostatin M Signaling Pathway	13	1.57081	1.44E-006
50	enriched	Development and heterogeneity of the ILC family	10	0.773321	1.58E-006
51	enriched	Adipogenesis	17	3.14162	3.12E-006
52	enriched	TLR4 Signaling and Tolerance	9	0.676656	6.05E-006
53	enriched	Prolactin Signaling Pathway	13	1.83664	6.71E-006
54	enriched	IL-5 Signaling Pathway	10	0.966652	8.45E-006
55	enriched	Microglia Pathogen Phagocytosis Pathway	10	0.966652	8.45E-006
56	enriched	Association Between Physico-Chemical Features and Toxicity Associated Pathways	12	1.59498	1.06E-005
57	enriched	Human Complement System	14	2.34413	1.36E-005
58	enriched	DNA Damage Response	12	1.64331	1.36E-005
59	enriched	Integrated Cancer Pathway	10	1.06332	1.59E-005
60	enriched	Interleukin-11 Signaling Pathway	10	1.06332	1.59E-005
61	enriched	MicroRNAs in cardiomyocyte hypertrophy	13	2.02997	1.59E-005
62	enriched	Signal transduction through IL1R	9	0.797488	1.59E-005
63	enriched	TGF-beta Signaling Pathway	16	3.18995	1.59E-005
64	enriched	miRNA regulation of prostate cancer signaling pathways	9	0.797488	1.59E-005

65	enriched	Non-genomic actions of 1,25 dihydroxyvitamin D3	12	1.71581	1.81E-005
66	enriched	miRNA Regulation of DNA Damage Response	12	1.71581	1.81E-005
67	enriched	Kit receptor signaling pathway	11	1.42581	2.24E-005
68	enriched	Chromosomal and microsatellite instability in colorectal cancer	12	1.76414	2.28E-005
69	enriched	Arrhythmogenic Right Ventricular Cardiomyopathy	12	1.78831	2.56E-005
70	enriched	Selenium Micronutrient Network	13	2.1508	2.56E-005
71	enriched	EPO Receptor Signaling	8	0.628323	2.84E-005
72	enriched	IL-9 Signaling Pathway	7	0.410827	2.84E-005
73	enriched	Serotonin and anxiety	7	0.410827	2.84E-005
74	enriched	Hepatitis C and Hepatocellular Carcinoma	10	1.18415	3.18E-005
75	enriched	Corticotropin-releasing hormone signaling pathway	13	2.2233	3.34E-005
76	enriched	Endometrial cancer	11	1.52248	3.51E-005
77	enriched	Vitamin D Receptor Pathway	18	4.39826	3.76E-005
78	enriched	DNA Damage Response (only ATM dependent)	14	2.65829	3.88E-005
79	enriched	Genes involved in male infertility	16	3.52828	4.18E-005
80	enriched	LTF danger signal response pathway	7	0.459159	4.64E-005
81	enriched	Overview of nanoparticle effects	7	0.459159	4.64E-005
82	enriched	AGE/RAGE pathway	11	1.59498	4.88E-005
83	enriched	IL-4 Signaling Pathway	10	1.30498	6.15E-005
84	enriched	RAC1/PAK1/p38/MMP2 Pathway	11	1.64331	6.17E-005
85	enriched	Integrated Breast Cancer Pathway	16	3.67328	6.29E-005
86	enriched	Cardiac Hypertrophic Response	10	1.32915	6.71E-005
87	enriched	Folate Metabolism	11	1.66747	6.71E-005
88	enriched	IL-1 signaling pathway	10	1.32915	6.71E-005
89	enriched	Toll-like Receptor Signaling related to MyD88	8	0.749155	7.13E-005
90	enriched	Nicotine Activity on Dopaminergic Neurons	7	0.507492	7.22E-005
91	enriched	IL-6 signaling pathway	9	1.03915	7.47E-005
92	enriched	Mammary gland development pathway - Pregnancy and lactation (Stage 3 of 4)	8	0.773321	8.44E-005
93	enriched	ATM Signaling Network in Development and Disease	9	1.08748	1.01E-004
94	enriched	Resistin as a regulator of inflammation	8	0.797488	1.01E-004
95	enriched	EGF/EGFR Signaling Pathway	16	3.89077	1.11E-004
96	enriched	The human immune response to tuberculosis	7	0.555825	1.12E-004
97	enriched	miRNAs involved in DNA damage response	6	0.362494	1.50E-004
98	enriched	IL-3 Signaling Pathway	9	1.18415	1.77E-004
99	enriched	Amyotrophic lateral sclerosis (ALS)	8	0.894153	1.98E-004
100	enriched	Nonalcoholic fatty liver disease	15	3.67328	2.25E-004
101	enriched	Sleep regulation	8	0.918319	2.30E-004
102	enriched	Head and Neck Squamous Cell Carcinoma	10	1.59498	2.42E-004
103	enriched	Human Thyroid Stimulating Hormone (TSH) signaling pathway	10	1.59498	2.42E-004
104	enriched	Breast cancer pathway	15	3.72161	2.45E-004
105	enriched	Cells and Molecules involved in local acute inflammatory response	6	0.410827	2.45E-004
106	enriched	Role of Altered Glycolysation of MUC1 in Tumour Microenvironment	5	0.217497	2.45E-004
107	enriched	PDGF Pathway	8	0.942485	2.57E-004

108	enriched	Integrin-mediated Cell Adhesion	12	2.4408	2.97E-004
109	enriched	PDGFR-beta pathway	7	0.700822	3.57E-004
110	enriched	DNA IR-Double Strand Breaks (DSBs) and cellular response via ATM	9	1.32915	3.60E-004
111	enriched	Fas Ligand (FasL) pathway and Stress induction of Heat Shock Proteins (HSP) regulation	8	1.01498	3.97E-004
112	enriched	Primary Focal Segmental Glomerulosclerosis FSGS	10	1.73997	4.41E-004
113	enriched	Apoptosis Modulation and Signaling	11	2.17497	5.07E-004
114	enriched	IL-10 Anti-inflammatory Signaling Pathway	5	0.289995	6.65E-004
115	enriched	Aryl Hydrocarbon Receptor Netpath	8	1.11165	6.84E-004
116	enriched	miRNA targets in ECM and membrane receptors	6	0.531658	7.56E-004
117	enriched	Control of immune tolerance by vasoactive intestinal peptide	5	0.314162	8.73E-004
118	enriched	Development of pulmonary dendritic cells and macrophage subsets	5	0.314162	8.73E-004
119	enriched	Estrogen signaling pathway	6	0.555825	9.11E-004
120	enriched	Overview of leukocyte-intrinsic Hippo pathway functions	7	0.84582	9.27E-004
121	enriched	Circadian rhythm related genes	16	4.85742	1.09E-003
122	enriched	Vitamin B12 Metabolism	8	1.20831	1.09E-003
123	enriched	Alzheimers Disease	10	1.98164	1.10E-003
124	enriched	Fragile X Syndrome	12	2.92412	1.32E-003
125	enriched	Mammary gland development pathway - Embryonic development (Stage 1 of 4)	5	0.362494	1.43E-003
126	enriched	Hair Follicle Development: Cytodifferentiation (Part 3 of 3)	10	2.10247	1.68E-003
127	enriched	Interferon type I signaling pathways	8	1.30498	1.71E-003
128	enriched	ATM Signaling Pathway	7	0.966652	1.83E-003
129	enriched	RANKL/RANK (Receptor activator of NFKB (ligand)) Signaling Pathway	8	1.32915	1.89E-003
130	enriched	Nanoparticle-mediated activation of receptor signaling	6	0.676656	2.14E-003
131	enriched	Platelet-mediated interactions with vascular and circulating cells	5	0.410827	2.22E-003
132	enriched	Regulation of Actin Cytoskeleton	13	3.62494	2.22E-003
133	enriched	Common Pathways Underlying Drug Addiction	7	1.01498	2.31E-003
134	enriched	TNF alpha Signaling Pathway	10	2.2233	2.41E-003
135	enriched	T-Cell Receptor and Co-stimulatory Signaling	6	0.700822	2.43E-003
136	enriched	MET in type 1 papillary renal cell carcinoma	8	1.42581	2.78E-003
137	enriched	Dopaminergic Neurogenesis	6	0.724989	2.82E-003
138	enriched	Novel intracellular components of RIG-I-like receptor (RLR) pathway	8	1.44998	3.04E-003
139	enriched	Extracellular vesicles in the crosstalk of cardiac cells	5	0.459159	3.25E-003
140	enriched	TP53 Network	5	0.459159	3.25E-003
141	enriched	Aryl Hydrocarbon Receptor Pathway	7	1.11165	3.61E-003
142	enriched	Monoamine Transport	6	0.773321	3.71E-003
143	enriched	DNA IR-damage and cellular response via ATR	9	1.9333	3.80E-003

144	enriched	Mechanoregulation and pathology of YAP/TAZ via Hippo and non-Hippo mechanisms	7	1.13582	3.95E-003
145	enriched	NO/cGMP/PKG mediated Neuroprotection	7	1.13582	3.95E-003
146	enriched	Cell Cycle	11	2.89995	4.16E-003
147	enriched	G1 to S cell cycle control	8	1.54664	4.25E-003
148	enriched	Signaling of Hepatocyte Growth Factor Receptor	6	0.821654	4.75E-003
149	enriched	Structural Pathway of Interleukin 1 (IL-1)	7	1.18415	4.83E-003
150	enriched	Synaptic signaling pathways associated with autism spectrum disorder	7	1.20831	5.36E-003
151	enriched	Vitamin D in inflammatory diseases	5	0.531658	5.39E-003
152	enriched	MicroRNA network associated with chronic lymphocytic leukemia	3	0.0966652	6.06E-003
153	enriched	EBV LMP1 signaling	5	0.555825	6.36E-003
154	enriched	Copper homeostasis	7	1.25665	6.43E-003
155	enriched	Wnt Signaling Pathway (Netpath)	7	1.25665	6.43E-003
156	enriched	Apoptosis-related network due to altered Notch3 in ovarian cancer	7	1.28081	7.09E-003
157	enriched	Androgen receptor signaling pathway	9	2.17497	7.67E-003
158	enriched	Mammary gland development pathway - Puberty (Stage 2 of 4)	4	0.314162	7.67E-003
159	enriched	Osteopontin Signaling	4	0.314162	7.67E-003
160	enriched	Serotonin and anxiety-related events	4	0.314162	7.67E-003
161	enriched	ncRNAs involved in STAT3 signaling in hepatocellular carcinoma	4	0.314162	7.67E-003
162	enriched	GPCRs, Other	9	2.19913	8.00E-003
163	enriched	Peptide GPCRs	8	1.78831	9.31E-003
164	enriched	GPCRs, Class A Rhodopsin-like	16	6.18657	1.04E-002
165	enriched	Insulin Signaling	12	3.86661	1.07E-002
166	enriched	TNF related weak inducer of apoptosis (TWEAK) Signaling Pathway	6	1.01498	1.16E-002
167	enriched	GPCRs, Class C Metabotropic glutamate, pheromone	4	0.362494	1.16E-002
168	enriched	Notch Signaling Pathway Netpath	7	1.47414	1.41E-002
169	enriched	Endochondral Ossification	7	1.54664	1.81E-002
170	enriched	Simplified Depiction of MYD88 Distinct Input-Output Pathway	4	0.434993	2.03E-002
171	enriched	Prion disease pathway	5	0.773321	2.12E-002
172	enriched	Retinoblastoma Gene in Cancer	8	2.10247	2.31E-002
173	enriched	Canonical NF-KB pathway	3	0.19333	2.34E-002
174	enriched	Hematopoietic Stem Cell Gene Regulation by GABP alpha/beta Complex	4	0.483326	2.78E-002
175	enriched	Phosphodiesterases in neuronal function	6	1.25665	2.97E-002
176	enriched	MECP2 and Associated Rett Syndrome	7	1.71581	2.98E-002
177	enriched	NLR Proteins	3	0.217497	2.98E-002
178	enriched	Supression of HMGB1 mediated inflammation by THBD	3	0.217497	2.98E-002
179	enriched	Overview of interferons-mediated signaling pathway	5	0.869986	3.22E-002
180	enriched	Nuclear Receptors Meta-Pathway	17	7.70905	3.33E-002
181	enriched	TGF-beta Receptor Signaling	6	1.30498	3.41E-002
182	enriched	PI3K/AKT/mTOR - VitD3 Signalling	4	0.531658	3.56E-002
183	enriched	Transcription factor regulation in adipogenesis	4	0.531658	3.56E-002

184	enriched	Type II diabetes mellitus	4	0.531658	3.56E-002
185	enriched	IL1 and megakaryocytes in obesity	4	0.579991	4.63E-002
186	enriched	Neural Crest Cell Migration during Development	5	0.966652	4.63E-002
187	enriched	Oxidative Damage	5	0.966652	4.63E-002
188	enriched	miRNA regulation of p53 pathway in prostate cancer	4	0.579991	4.63E-002

Table S2.5. Reactome-Pathways enriched by key network targets of RA-Hot

No	Type	Name	Number of hits	Expected score	Adjusted p-value
1	enriched	Interleukin-4 and Interleukin-13 signaling	31	2.60996	3.99E-018
2	enriched	Interleukin-10 signaling	19	1.13582	9.08E-013
3	enriched	Highly calcium permeable postsynaptic nicotinic acetylcholine receptors	11	0.289995	1.35E-009
4	enriched	Interferon gamma signaling	19	2.0783	4.12E-009
5	enriched	G alpha (i) signalling events	30	6.33157	1.05E-008
6	enriched	Generation of second messenger molecules	13	0.797488	1.71E-008
7	enriched	Highly calcium permeable nicotinic acetylcholine receptors	9	0.217497	3.71E-008
8	enriched	Immunoregulatory interactions between a Lymphoid and a non-Lymphoid cell	21	3.18995	3.71E-008
9	enriched	Phosphorylation of CD3 and TCR zeta chains	11	0.531658	5.20E-008
10	enriched	Chemokine receptors bind chemokines	15	1.42581	5.49E-008
11	enriched	Translocation of ZAP-70 to Immunological synapse	10	0.459159	2.03E-007
12	enriched	Downstream TCR signaling	17	2.3683	4.88E-007
13	enriched	Interaction between L1 and Ankyrins	11	0.749155	6.57E-007
14	enriched	PD-1 signaling	10	0.555825	6.79E-007
15	enriched	RAF/MAP kinase cascade	18	3.02079	1.81E-006
16	enriched	Highly sodium permeable acetylcholine nicotinic receptors	7	0.169164	2.48E-006
17	enriched	Integrin cell surface interactions	15	2.05413	2.56E-006
18	enriched	DNA Damage/Telomere Stress Induced Senescence	13	1.59498	7.28E-006
19	enriched	Interleukin-7 signaling	10	0.869986	1.67E-005
20	enriched	Cell surface interactions at the vascular wall	14	2.12663	2.10E-005
21	enriched	Neutrophil degranulation	32	11.5998	1.02E-004
22	enriched	Interleukin-23 signaling	6	0.217497	1.09E-004
23	enriched	Constitutive Signaling by Aberrant PI3K in Cancer	12	1.81247	1.38E-004
24	enriched	Recruitment and ATM-mediated phosphorylation of repair and signaling proteins at DNA double strand breaks	12	1.83664	1.50E-004
25	enriched	Interleukin receptor SHC signaling	8	0.65249	1.67E-004
26	enriched	PIP3 activates AKT signaling	13	2.24746	1.70E-004
27	enriched	Non-integrin membrane-ECM interactions	9	0.966652	2.37E-004
28	enriched	Interferon alpha/beta signaling	11	1.61914	2.58E-004
29	enriched	ER-Phagosome pathway	12	2.0058	2.75E-004
30	enriched	TNFR1-induced NFkappaB signaling pathway	8	0.724989	2.75E-004
31	enriched	G2/M DNA damage checkpoint	12	2.02997	2.83E-004
32	enriched	NCAM1 interactions	9	1.01498	2.83E-004
33	enriched	Nonhomologous End-Joining (NHEJ)	11	1.66747	2.83E-004

34	enriched	Antigen activates B Cell Receptor (BCR) leading to generation of second messengers	8	0.773321	3.53E-004
35	enriched	PI5P, PP2A and IER3 Regulate PI3K/AKT Signaling	13	2.48913	3.53E-004
36	enriched	Regulation of TNFR1 signaling	8	0.797488	4.16E-004
37	enriched	Phase 0 - rapid depolarisation	9	1.11165	4.73E-004
38	enriched	RHO GTPases Activate NADPH Oxidases	7	0.579991	5.66E-004
39	enriched	Senescence-Associated Secretory Phenotype (SASP)	13	2.65829	6.09E-004
40	enriched	Cross-presentation of particulate exogenous antigens (phagosomes)	5	0.19333	6.76E-004
41	enriched	Detoxification of Reactive Oxygen Species	8	0.894153	7.59E-004
42	enriched	Assembly of collagen fibrils and other multimeric structures	9	1.23248	8.72E-004
43	enriched	RUNX3 regulates p14-ARF	5	0.217497	9.59E-004
44	enriched	Interleukin-21 signaling	5	0.241663	1.38E-003
45	enriched	Interleukin-1 signaling	11	2.12663	1.67E-003
46	enriched	Platelet degranulation	13	3.09328	2.26E-003
47	enriched	ECM proteoglycans	10	1.83664	2.42E-003
48	enriched	Interleukin-2 signaling	5	0.289995	2.42E-003
49	enriched	Interleukin-35 Signalling	5	0.289995	2.42E-003
50	enriched	RUNX1 regulates transcription of genes involved in differentiation of HSCs	13	3.14162	2.42E-003
51	enriched	Paradoxical activation of RAF signaling by kinase inactive BRAF	8	1.13582	2.70E-003
52	enriched	Signaling by moderate kinase activity BRAF mutants	8	1.13582	2.70E-003
53	enriched	Signaling downstream of RAS mutants	8	1.13582	2.70E-003
54	enriched	ROS and RNS production in phagocytes	7	0.821654	2.78E-003
55	enriched	Integrin alphaIIb beta3 signaling	6	0.555825	3.12E-003
56	enriched	Collagen degradation	9	1.54664	3.28E-003
57	enriched	Estrogen-dependent nuclear events downstream of ESR-membrane signaling	6	0.579991	3.57E-003
58	enriched	Growth hormone receptor signaling	6	0.579991	3.57E-003
59	enriched	Signaling by high-kinase activity BRAF mutants	7	0.869986	3.57E-003
60	enriched	Regulation of IFNG signaling	5	0.338328	3.62E-003
61	enriched	Interleukin-20 family signaling	6	0.604157	4.19E-003
62	enriched	GRB2:SOS provides linkage to MAPK signaling for Integrins	5	0.362494	4.37E-003
63	enriched	Packaging Of Telomere Ends	8	1.25665	4.37E-003
64	enriched	The NLRP3 inflammasome	5	0.362494	4.37E-003
65	enriched	p130Cas linkage to MAPK signaling for integrins	5	0.362494	4.37E-003
66	enriched	Meiotic recombination	10	2.0783	4.59E-003
67	enriched	RUNX3 regulates CDKN1A transcription	4	0.169164	4.59E-003
68	enriched	MAP2K and MAPK activation	7	0.966652	5.38E-003
69	enriched	Syndecan interactions	6	0.65249	5.38E-003
70	enriched	Cleavage of the damaged purine	8	1.35331	6.02E-003
71	enriched	Recognition and association of DNA glycosylase with site containing an affected purine	8	1.35331	6.02E-003
72	enriched	Transcriptional regulation of granulopoiesis	10	2.17497	6.02E-003
73	enriched	TNFs bind their physiological receptors	6	0.700822	7.13E-003
74	enriched	Formation of Incision Complex in GG-NER	7	1.03915	7.40E-003

75	enriched	Pre-NOTCH Transcription and Translation	10	2.24746	7.40E-003
76	enriched	DAP12 signaling	6	0.724989	7.95E-003
77	enriched	MET activates PTK2 signaling	6	0.724989	7.95E-003
78	enriched	Cyclin D associated events in G1	7	1.06332	8.02E-003
79	enriched	Interleukin-9 signaling	4	0.217497	8.02E-003
80	enriched	Cleavage of the damaged pyrimidine	8	1.47414	9.08E-003
81	enriched	Recognition and association of DNA glycosylase with site containing an affected pyrimidine	8	1.47414	9.08E-003
82	enriched	Processing of DNA double-strand break ends	10	2.3683	1.00E-002
83	enriched	IKBKB deficiency causes SCID	3	0.0724989	1.01E-002
84	enriched	IKBKG deficiency causes anhidrotic ectodermal dysplasia with immunodeficiency (EDA-ID) (via TLR)	3	0.0724989	1.01E-002
85	enriched	Interleukin-12 signaling	4	0.241663	1.01E-002
86	enriched	RUNX1 and FOXP3 control the development of regulatory T lymphocytes (Tregs)	4	0.241663	1.01E-002
87	enriched	Signal transduction by L1	5	0.507492	1.23E-002
88	enriched	Interleukin-6 signaling	4	0.265829	1.31E-002
89	enriched	Type I hemidesmosome assembly	4	0.265829	1.31E-002
90	enriched	Activation of NF-kappaB in B cells	8	1.61914	1.41E-002
91	enriched	Common Pathway of Fibrin Clot Formation	5	0.531658	1.41E-002
92	enriched	GPVI-mediated activation cascade	6	0.84582	1.41E-002
93	enriched	Signaling by BRAF and RAF fusions	8	1.61914	1.41E-002
94	enriched	NOD1/2 Signaling Pathway	6	0.869986	1.56E-002
95	enriched	TP53 Regulates Transcription of Caspase Activators and Caspases	4	0.289995	1.60E-002
96	enriched	Factors involved in megakaryocyte development and platelet production	10	2.63413	1.87E-002
97	enriched	Gene and protein expression by JAK-STAT signaling after Interleukin-12 stimulation	6	0.918319	1.95E-002
98	enriched	Antigen Presentation: Folding, assembly and peptide loading of class I MHC	5	0.604157	2.15E-002
99	enriched	IKK complex recruitment mediated by RIP1	5	0.604157	2.15E-002
100	enriched	Role of phospholipids in phagocytosis	5	0.604157	2.15E-002
101	enriched	AKT phosphorylates targets in the cytosol	4	0.338328	2.42E-002
102	enriched	Interleukin-15 signaling	4	0.338328	2.42E-002
103	enriched	Resolution of D-loop Structures through Synthesis-Dependent Strand Annealing (SDSA)	5	0.628323	2.44E-002
104	enriched	Dual Incision in GG-NER	6	0.990818	2.61E-002
105	enriched	RUNX3 Regulates Immune Response and Cell Migration	3	0.144998	3.17E-002
106	enriched	Telomere Extension By Telomerase	3	0.144998	3.17E-002
107	enriched	Voltage gated Potassium channels	6	1.03915	3.17E-002
108	enriched	Meiotic synapsis	8	1.90914	3.29E-002
109	enriched	Collagen chain trimerization	6	1.06332	3.35E-002
110	enriched	MAP3K8 (TPL2)-dependent MAPK1/3 activation	4	0.386661	3.35E-002
111	enriched	PI3K events in ERBB2 signaling	4	0.386661	3.35E-002
112	enriched	RIPK1-mediated regulated necrosis	4	0.386661	3.35E-002
113	enriched	Regulation of actin dynamics for phagocytic cup formation	7	1.47414	3.35E-002
114	enriched	CLEC7A (Dectin-1) signaling	8	1.9333	3.36E-002
115	enriched	VEGFA-VEGFR2 Pathway	7	1.49831	3.55E-002

116	enriched	FCERI mediated NF-kB activation	8	1.98164	3.83E-002
117	enriched	GAB1 signalosome	4	0.410827	3.84E-002
118	enriched	MECP2 regulates neuronal receptors and channels	4	0.410827	3.84E-002
119	enriched	RIP-mediated NFkB activation via ZBP1	4	0.410827	3.84E-002
120	enriched	IkBA variant leads to EDA-ID	3	0.169164	3.95E-002
121	enriched	Regulation of Complement cascade	6	1.13582	4.25E-002
122	enriched	DNA methylation	7	1.57081	4.30E-002
123	enriched	Estrogen-dependent gene expression	11	3.62494	4.38E-002
124	enriched	Signaling by cytosolic FGFR1 fusion mutants	4	0.434993	4.38E-002
125	enriched	TNF receptor superfamily (TNFSF) members mediating non-canonical NF-kB pathway	4	0.434993	4.38E-002
126	enriched	Cargo recognition for clathrin-mediated endocytosis	9	2.56163	4.39E-002
127	enriched	NoRC negatively regulates rRNA expression	9	2.56163	4.39E-002
128	enriched	Activated PKN1 stimulates transcription of AR (androgen receptor) regulated genes KLK2 and KLK3	7	1.61914	4.81E-002
129	enriched	Amyloid fiber formation	9	2.60996	4.87E-002
130	enriched	Interleukin-1 processing	3	0.19333	4.89E-002
131	enriched	MyD88:MAL(TIRAP) cascade initiated on plasma membrane	4	0.459159	4.89E-002
132	enriched	Platelet homeostasis	3	0.19333	4.89E-002
133	enriched	Regulation of TLR by endogenous ligand	4	0.459159	4.89E-002

Table S2.6. KEGG-Pathways enriched by key network targets of RA-Hot

No	Type	Name	Number of hits	Expected score	Adjusted p-value
1	enriched	Pathways in cancer	66	12.784	6.24E-023
2	enriched	PI3K-Akt signaling pathway	53	8.5307	1.57E-021
3	enriched	Primary immunodeficiency	24	0.869986	3.86E-021
4	enriched	Cytokine-cytokine receptor interaction	48	7.12906	6.22E-021
5	enriched	Epstein-Barr virus infection	40	4.83326	3.89E-020
6	enriched	Hematopoietic cell lineage	30	2.31996	1.63E-019
7	enriched	Human T-cell leukemia virus 1 infection	40	5.29242	5.08E-019
8	enriched	Leishmaniasis	27	1.78831	5.17E-019
9	enriched	Th17 cell differentiation	30	2.58579	1.46E-018
10	enriched	Toxoplasmosis	30	2.70662	3.94E-018
11	enriched	Neuroactive ligand-receptor interaction	46	8.26487	1.94E-017
12	enriched	Influenza A	33	4.0841	1.74E-016
13	enriched	Th1 and Th2 cell differentiation	26	2.2233	3.43E-016
14	enriched	Jak-STAT signaling pathway	32	3.91494	3.74E-016
15	enriched	Measles	30	3.33495	3.74E-016
16	enriched	Osteoclast differentiation	29	3.04495	3.74E-016
17	enriched	Inflammatory bowel disease (IBD)	23	1.57081	3.88E-016
18	enriched	NF-kappa B signaling pathway	26	2.46496	2.12E-015
19	enriched	Human papillomavirus infection	42	7.95071	2.51E-015
20	enriched	Human cytomegalovirus infection	35	5.43741	5.17E-015
21	enriched	T cell receptor signaling pathway	25	2.48913	2.00E-014
22	enriched	NOD-like receptor signaling pathway	31	4.34993	2.39E-014
23	enriched	PD-L1 expression and PD-1 checkpoint pathway in cancer	23	2.17497	1.11E-013
24	enriched	Chagas disease (American trypanosomiasis)	24	2.46496	1.26E-013
25	enriched	Human immunodeficiency virus 1 infection	32	5.09909	1.72E-013

26	enriched	Yersinia infection	25	2.89995	3.45E-013
27	enriched	B cell receptor signaling pathway	21	1.9333	1.03E-012
28	enriched	Toll-like receptor signaling pathway	23	2.53746	1.56E-012
29	enriched	Cell adhesion molecules (CAMs)	26	3.50411	1.98E-012
30	enriched	Allograft rejection	16	0.894153	2.19E-012
31	enriched	Chemokine signaling pathway	29	4.56743	2.19E-012
32	enriched	Hepatitis B	27	3.91494	2.76E-012
33	enriched	Intestinal immune network for IgA	17	1.13582	3.48E-012
34	enriched	Viral protein interaction with cytokine and cytokine receptor	22	2.41663	4.35E-012
35	enriched	Viral myocarditis	18	1.42581	6.82E-012
36	enriched	Kaposi sarcoma-associated herpesvirus infection	28	4.49493	7.90E-012
37	enriched	Type I diabetes mellitus	16	1.03915	1.18E-011
38	enriched	Tuberculosis	27	4.27743	1.53E-011
39	enriched	MAPK signaling pathway	34	7.10489	1.82E-011
40	enriched	Focal adhesion	28	4.80909	3.12E-011
41	enriched	Proteoglycans in cancer	28	4.85742	3.78E-011
42	enriched	C-type lectin receptor signaling pathway	21	2.51329	5.43E-011
43	enriched	Rheumatoid arthritis	20	2.2233	5.55E-011
44	enriched	FoxO signaling pathway	23	3.16578	5.77E-011
45	enriched	Graft-versus-host disease	15	0.990818	6.58E-011
46	enriched	MicroRNAs in cancer	25	3.89077	6.70E-011
47	enriched	Natural killer cell mediated cytotoxicity	22	3.14162	3.13E-010
48	enriched	Small cell lung cancer	19	2.2233	3.92E-010
49	enriched	Pancreatic cancer	17	1.81247	1.30E-009
50	enriched	AGE-RAGE signaling pathway in diabetic complications	19	2.41663	1.33E-009
51	enriched	African trypanosomiasis	13	0.869986	2.00E-009
52	enriched	Pertussis	17	1.88497	2.10E-009
53	enriched	Necroptosis	23	3.9391	2.50E-009
54	enriched	Fluid shear stress and atherosclerosis	21	3.33495	4.61E-009
55	enriched	Malaria	14	1.18415	4.61E-009
56	enriched	TNF signaling pathway	19	2.70662	6.59E-009
57	enriched	Autoimmune thyroid disease	14	1.25665	8.54E-009
58	enriched	Chronic myeloid leukemia	16	1.83664	1.06E-008
59	enriched	Ras signaling pathway	26	5.58241	1.19E-008
60	enriched	Systemic lupus erythematosus	20	3.21412	1.39E-008
61	enriched	Transcriptional misregulation in cancer	23	4.47076	2.06E-008
62	enriched	Cholinergic synapse	18	2.73079	4.32E-008
63	enriched	Platinum drug resistance	15	1.76414	4.60E-008
64	enriched	ECM-receptor interaction	16	2.12663	6.23E-008
65	enriched	Cytosolic DNA-sensing pathway	14	1.52248	6.41E-008
66	enriched	Apoptosis	19	3.26245	9.07E-008
67	enriched	Non-small cell lung cancer	14	1.59498	1.05E-007
68	enriched	Hepatitis C	20	3.74577	1.32E-007
69	enriched	Cellular senescence	20	3.86661	2.12E-007
70	enriched	Amoebiasis	16	2.41663	2.88E-007
71	enriched	Phagosome	19	3.62494	3.96E-007
72	enriched	Hypertrophic cardiomyopathy (HCM)	15	2.17497	4.89E-007
73	enriched	Herpes simplex virus 1 infection	35	11.8415	7.40E-007
74	enriched	Calcium signaling pathway	21	4.66409	7.65E-007
75	enriched	HIF-1 signaling pathway	16	2.63413	7.93E-007
76	enriched	Staphylococcus aureus infection	15	2.2958	8.78E-007
77	enriched	Prostate cancer	15	2.34413	1.11E-006

78	enriched	Viral carcinogenesis	21	4.85742	1.37E-006
79	enriched	Colorectal cancer	14	2.0783	1.69E-006
80	enriched	Antigen processing and presentation	13	1.8608	3.26E-006
81	enriched	Regulation of actin cytoskeleton	21	5.14742	3.26E-006
82	enriched	cAMP signaling pathway	21	5.21992	3.95E-006
83	enriched	EGFR tyrosine kinase inhibitor resistance	13	1.90914	4.14E-006
84	enriched	IL-17 signaling pathway	14	2.27163	4.23E-006
85	enriched	Bladder cancer	10	0.990818	4.32E-006
86	enriched	Salmonella infection	13	1.9333	4.55E-006
87	enriched	Dilated cardiomyopathy (DCM)	14	2.2958	4.59E-006
88	enriched	Gastric cancer	17	3.55244	5.79E-006
89	enriched	Legionellosis	11	1.35331	6.94E-006
90	enriched	Prolactin signaling pathway	12	1.69164	7.52E-006
91	enriched	ErbB signaling pathway	13	2.05413	7.99E-006
92	enriched	Rap1 signaling pathway	20	5.07492	9.21E-006
93	enriched	Prion diseases	9	0.84582	1.08E-005
94	enriched	Thyroid cancer	9	0.894153	1.59E-005
95	enriched	Arrhythmogenic right ventricular cardiomyopathy (ARVC)	12	1.8608	1.76E-005
96	enriched	Amyotrophic lateral sclerosis (ALS)	10	1.20831	1.87E-005
97	enriched	Acute myeloid leukemia	11	1.61914	3.05E-005
98	enriched	Endocrine resistance	13	2.3683	3.14E-005
99	enriched	Asthma	8	0.724989	3.38E-005
100	enriched	Pathogenic Escherichia coli infection	18	4.85742	6.85E-005
101	enriched	Glioma	11	1.81247	7.78E-005
102	enriched	Fc gamma R-mediated phagocytosis	12	2.2233	8.66E-005
103	enriched	Non-alcoholic fatty liver disease (NAFLD)	15	3.52828	9.08E-005
104	enriched	Leukocyte transendothelial migration	13	2.70662	1.12E-004
105	enriched	Taste transduction	11	2.0058	1.78E-004
106	enriched	Neurotrophin signaling pathway	13	2.87579	1.99E-004
107	enriched	Adipocytokine signaling pathway	10	1.66747	2.13E-004
108	enriched	Central carbon metabolism in cancer	10	1.66747	2.13E-004
109	enriched	Nicotine addiction	8	0.990818	2.25E-004
110	enriched	RIG-I-like receptor signaling pathway	10	1.69164	2.34E-004
111	enriched	Alcoholism	16	4.4466	2.81E-004
112	enriched	Melanoma	10	1.73997	2.81E-004
113	enriched	Platelet activation	13	2.99662	2.81E-004
114	enriched	p53 signaling pathway	10	1.73997	2.81E-004
115	enriched	Antifolate resistance	7	0.749155	3.19E-004
116	enriched	Endometrial cancer	9	1.40164	3.24E-004
117	enriched	Hepatocellular carcinoma	15	4.03577	3.47E-004
118	enriched	Cocaine addiction	8	1.18415	6.47E-004
119	enriched	Growth hormone synthesis, secretion and action	12	2.87579	7.62E-004
120	enriched	Sphingolipid signaling pathway	12	2.87579	7.62E-004
121	enriched	Aldosterone-regulated sodium reabsorption	7	0.894153	8.15E-004
122	enriched	Fc epsilon RI signaling pathway	9	1.61914	8.50E-004
123	enriched	Epithelial cell signaling in Helicobacter pylori infection	9	1.69164	1.14E-003
124	enriched	Breast cancer	13	3.55244	1.27E-003
125	enriched	Serotonergic synapse	11	2.77912	2.24E-003
126	enriched	Estrogen signaling pathway	12	3.33495	2.62E-003
127	enriched	Type II diabetes mellitus	7	1.11165	2.62E-003
128	enriched	Complement and coagulation cascades	9	1.9333	2.76E-003
129	enriched	Thyroid hormone signaling pathway	11	2.87579	2.86E-003

130	enriched	Nucleotide excision repair	7	1.13582	2.86E-003
131	enriched	Inflammatory mediator regulation of TRP channels	10	2.41663	2.98E-003
132	enriched	GnRH secretion	8	1.54664	3.08E-003
133	enriched	Alzheimer disease	13	4.03577	3.80E-003
134	enriched	Cell cycle	11	2.99662	3.80E-003
135	enriched	Huntington disease	14	4.59159	3.80E-003
136	enriched	Phospholipase D signaling pathway	12	3.55244	4.17E-003
137	enriched	Oxytocin signaling pathway	12	3.69744	5.78E-003
138	enriched	Cushing syndrome	12	3.76994	6.74E-003
139	enriched	Glutamatergic synapse	10	2.73079	6.86E-003
140	enriched	VEGF signaling pathway	7	1.42581	9.16E-003
141	enriched	Choline metabolism in cancer	9	2.3683	9.74E-003
142	enriched	Long-term potentiation	7	1.61914	1.78E-002
143	enriched	Insulin resistance	9	2.60996	1.80E-002
144	enriched	Amphetamine addiction	7	1.64331	1.89E-002
145	enriched	Renal cell carcinoma	7	1.66747	2.03E-002
146	enriched	Signaling pathways regulating pluripotency of stem cells	10	3.38328	2.91E-002
147	enriched	Protein digestion and absorption	8	2.2958	2.91E-002
148	enriched	Long-term depression	6	1.47414	4.70E-002
