# Supplementary material for: Integrative network fusion-based multi-omics study for biomarker identification and patient classification of rheumatoid arthritis
Source: Chin Med. 2023 May 4;18:48. doi: 10.1186/s13020-023-00750-8 (PMC10158004; doi:10.1186/s13020-023-00750-8)
Supplement: Supplementary file 3 — Additional file 3: Table S3. Differential metabolites of serum and synovial fluid samples from RA-Cold and RA-Hot patients detected by GC/MS. [file 13020_2023_750_MOESM3_ESM.pdf]

Table S3. Differential metabolites of serum and synovial fluid samples from RA-Cold and RA-Hot patients detected by GC/MS

Groups	Sample Type	Metabolite Name	CAS NO.	Formula	Exact mass	Structure	Concentration (matrice)		
							Normal	Cold-RA	Hot-RA
Normal vs RA-hot	Serum	Acetohydroxamic acid	546-88-3	$C_2H_5NO_2$	75.032	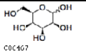	1006095.33	1005162.63	993834.75
Normal vs RA-hot	Serum	Alpha tocophereol	1959/2/9	$C_{29}H_{50}O_2$	430.72	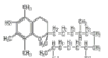	52803	83831.5	65121.25
Normal vs RA-hot	Serum	Beta-alanine	107-95-9	$C_3H_7NO_2$	89.0477	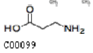	74753	78244.88	55264.17
Normal vs RA-hot	Synovial fluid	Cholesterol	57-88-5	$C_{27}H_{46}O$	386.355	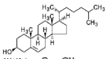	4692091.11	0	5378448.72
Normal vs RA-hot	Synovial fluid	D (+)altrose	1990-29-0	$C_6H_{12}O_6$	180.063	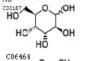	32289778.18	29992401.63	25658031.13
Normal vs RA-hot	Synovial fluid	D-allose	2595-97-3	$C_6H_{12}O_6$	180.063	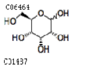	2259120.53	1840082.95	1376830.33
Normal vs RA-hot	Serum	DL-isoleucine	443-79-8	$C_6H_{13}NO_2$	131.095	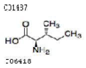	65812.33	102672.13	137339
Normal vs RA-hot	Synovial fluid/Serum	L-(+) lactic acid	79-33-4	$C_3H_6O_3$	90.0317	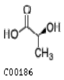	13368294.82<br>17249014	19850532.19<br>16295883	23661202.27<br>10156174.67
Normal vs RA-hot	Serum	L-alanine	56-41-7	$C_3H_7NO_2$	89.0477	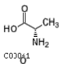	833952.33	916189.63	926338.92
Normal vs RA-hot	Synovial fluid	Palmitic acid	1957/10/3	$C_{16}H_{32}O_2$	256.24	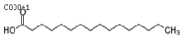	5417091.69	5216692.15	4764318.79
Normal vs RA-cold	Synovial fluid	Cholesterol	57-88-5	$C_{27}H_{46}O$	386.355	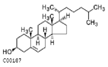	4692091.11	0	5378448.72
Normal vs RA-cold	Synovial fluid/Serum	D (+)altrose	1990-29-0	$C_6H_{12}O_6$	180.063	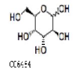	32289778.18<br>37786875.67	29992401.63<br>44074930	25658031.13<br>43446273.92
Normal vs RA-cold	Synovial fluid	D-allose	2595-97-3	$C_6H_{12}O_6$	180.063	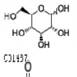	2259120.53	1840082.95	1376830.33
Normal vs RA-cold	Synovial fluid/Serum	L-(+) lactic acid	79-33-4	$C_3H_6O_3$	90.0317	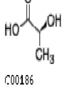	13368294.82<br>17249014	19850532.19<br>16295883	23661202.27<br>10156174.67
Normal vs RA-cold	Synovial fluid	L-pyroglutamic acid	98-79-3	$C_5H_7NO_3$	129.043	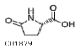	1648197.4	1666824.72	1555451.01
Normal vs RA-cold	Synovial fluid/Serum	Oleic acid	112-80-1	$C_{18}H_{34}O_2$	282.256	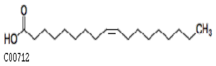	930273.73<br>1556383.33	45755.17<br>905054	22725.21<br>1007219.5
Normal vs RA-cold	Synovial fluid	Palmitic acid	1957/10/3	$C_{16}H_{32}O_2$	256.24	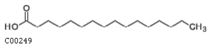	5417091.69	5216692.15	4764318.79
Normal vs RA-cold	Synovial fluid/Serum	Psicose	551-68-8	$C_6H_{12}O_6$	180.063	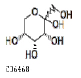	99696.56<br>3628214.333	901211.6205<br>4344456.875	230747.72<br>2882035.17
Normal vs RA-cold	Synovial fluid	Talose	2595-98-4	$C_6H_{12}O_6$	180.063	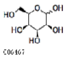	5003009.24	4658396.49	4355762.05
RA-Hot vs RA-cold	Synovial fluid/Serum	L-(+) lactic acid	79-33-4	$C_3H_6O_3$	90.0317	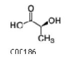	13368294.82<br>17249014	19850532.19<br>16295883	23661202.27<br>10156174.67
RA-Hot vs RA-cold	Serum	Acetohydroxamic acid	546-88-3	$C_2H_5NO_2$	75.032	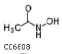	1006095.33	1005162.63	993834.75
RA-Hot vs RA-cold	Serum	Alpha tocophereol	1959/2/9	$C_{29}H_{50}O_2$	430.72	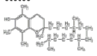	52803	83831.5	65121.25
RA-Hot vs RA-cold	Serum	Benzyl alcohol	100-51-6	C_7H_8O	108.058	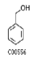	/	/	/
RA-Hot vs RA-cold	Serum	Beta-alanine	107-95-9	$C_3H_7NO_2$	89.0477	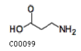	74753	78244.88	55264.17
RA-Hot vs RA-cold	Synovial fluid	Cholesterol	57-88-5	$C_{27}H_{46}O$	386.355	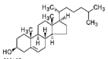	4692091.11	0	5378448.72
RA-Hot vs RA-cold	Synovial fluid	D (+)altrose	1990-29-0	$C_6H_{12}O_6$	180.063	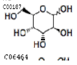	32289778.18	29992401.63	25658031.13

RA-Hot vs RA-cold	Synovial fluid	D-allose	2595-97-3	$C_6H_{12}O_6$	180.063	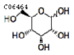	2259120.53	1840082.95	1376830.33
RA-Hot vs RA-cold	Serum	DL-isoleucine	443-79-8	$C_6H_{13}NO_2$	131.095	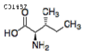	65812.33	102672.13	137339
RA-Hot vs RA-cold	Synovial fluid	Palmitic acid	1957/10/3	$C_{16}H_{32}O_2$	256.24	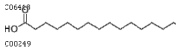	5417091.69	5216692.15	4764318.79
RA-Hot vs RA-cold	Synovial fluid	Stearic acid	1957/11/4	$C_{18}H_{36}O_2$	284.272	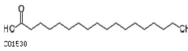	2886873.88	3002380.67	2691662.12
RA-Hot vs RA-cold	Synovial fluid	Talose	2595-98-4	$C_6H_{12}O_6$	180.063	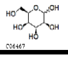	5003009.24	4658396.49	4355762.05
