# Supplementary material for: Integrative network fusion-based multi-omics study for biomarker identification and patient classification of rheumatoid arthritis
Source: Chin Med. 2023 May 4;18:48. doi: 10.1186/s13020-023-00750-8 (PMC10158004; doi:10.1186/s13020-023-00750-8)
Supplement: Supplementary file 4 — Additional file 4. Differential metabolites of serum and synovial fluid samples from RA-Cold and RA-Hot patients detected by LC/MS: Table S4.1. Total differential metabolites of serum and synovial fluid samples from RA-Cold and RA-Hot patients detected by LC/MS. Table S4.2. Significantly differential metabolites of serum and synovial fluid samples from RA-Cold and RA-Hot patients detected by LC/MS. [file 13020_2023_750_MOESM4_ESM.pdf]

Table S4.1. Total differential metabolites of serum and synovial fluid samples from RA-Cold and RA-Hot patients detected by LC/MS

Sample type	Groups	tR(s)	*m/z* (Da)	Score	English Name	Molecular Formula	CAS No.	FC	P (T test)	VIP
Serum	cold *vs.* normal	196.510	129.057	0.980	4-Methyl-2-Oxovalerate	$C_6H_{10}O_3$	4502-00-5	1.743	0.262	1.040
Serum	cold *vs.* hot; cold *vs.* normal; hot *vs.* normal; RA *vs.* normal	100.580	146.985	1.000	3-Acetyl-1,2-dithiolane	$C_5H_8OS_2$	89712-89-0	1.771	0.073	1.709
Serum	cold *vs.* hot; cold *vs.* normal; cold *vs.* hot; RA *vs.* normal	299.710	213.025	0.280	Clofibric acid|Clofibric acid (INN)|CLOF	$C_{10}H_{11}ClO_3$	882-09-7	1.855	0.168	1.318
Serum	cold *vs.* hot	346.820	227.131	0.060	Pro-Leu	$C_{11}H_{20}N_2O_3$	52899-07-7	1.652	0.063	1.635
Serum	RA *vs.* normal	391.840	239.062	0.440	Dinoterb|2-tert-butyl-4,6-dinitro-phenol|P	$C_{10}H_{12}N_2O_5$	1420-07-1	1.572	0.138	1.047
Serum	cold *vs.* hot	397.720	267.131	0.570	3-carboxy-4-methyl-5-pentyl-2-furanprop	$C_{14}H_{20}O_5$		2.477	0.076	1.594
Serum	cold *vs.* hot; RA *vs.* normal	636.580	371.223	0.650	5-Ethoxysorgoleone 358	$C_{23}H_{32}O_4$	210708-13-	0.489	0.271	1.186
Serum	cold *vs.* hot	372.000	375.197	1.000	Fluoromethalone	$C_{22}H_{29}FO_4$	426-13-1	1.753	0.322	1.007
Serum	cold *vs.* hot	569.090	415.226	0.700	Ramipril (USP/INN)|Altace (TN)|Ramipr	$C_{23}H_{32}N_2O_5$	87333-19-5	0.716	0.060	1.648
Serum	hot *vs.* normal; RA *vs.* normal	582.720	417.317	0.580	β-Citraurinene	$C_{30}H_{42}O$	58947-97-0	1.587	0.100	1.026
Serum	cold *vs.* normal	614.070	479.342	0.870	24-epi-Brassinolide|Brassinolide|2,4-Epil	$C_{28}H_{48}O_6$	78821-43-9<br>72962-43-7	2.169	0.141	1.299
Serum	cold *vs.* normal; hot *vs.* normal; RA *vs.* normal	441.980	507.277	1.000	LPG 18:2	$C_{24}H_{45}O_9P$		1.644	0.187	1.201
Serum	cold *vs.* normal	862.080	572.501	1.000	Cer-NS d37:4|Cer-NS d24:3/13:1|Cer[NS	$C_{37}H_{67}NO_3$		0.543	0.344	1.150
Serum	cold *vs.* normal; hot *vs.* normal; RA *vs.* normal	815.730	762.516	0.380	Phosphatidylethanolamine 18:2-20:4	$C_{43}H_{74}NO_8P$		1.875	0.131	1.375
Serum	cold *vs.* hot	747.090	778.595	0.920	Cer-BDS d47:9|Cer_BS d20:2/27:7|Cer[	$C_{47}H_{77}NO_4$		0.739	0.526	1.024
Serum	cold *vs.* hot; RA *vs.* normal	851.460	789.618	0.210	Sphingomyelin d18:1-C18:0	$C_{41}H_{83}N_2O_6P$	54336-69-5	1.305	0.329	1.030
Serum	cold *vs.* normal	848.710	790.593	0.670	Cer-BDS d48:10|Cer_BS d21:3/27:7|Cer|	$C_{48}H_{77}NO_4$		0.507	0.314	1.002
Serum	hot *vs.* normal	716.520	819.496	0.460	PI 33:2|PI 16:1-17:1|PI(16:1/17:1)	$C_{42}H_{77}O_{13}P$		1.230	0.030	1.344
Serum	cold *vs.* normal	887.920	819.622	0.250	Phosphatidylcholine alkyl 18:0-22:6	$C_{48}H_{86}NO_7P$	139406-71-	0.715	0.367	1.163
Serum	cold *vs.* normal	752.080	835.527	0.900	Roxithromycin	$C_{41}H_{76}N_2O_{15}$	80214-83-1	2.210	0.139	1.293
Serum	cold *vs.* hot	926.480	858.630	1.000	HexCer-AP t39:2|HexCer-AP t20:1/19:1|	$C_{45}H_{85}NO_{10}$		0.204	0.272	1.082
Serum	cold *vs.* normal	812.940	862.561	0.920	Phosphatidylcholine 16:0-22:7	$C_{47}H_{80}NO_8P$		2.598	0.017	1.978
Serum	hot *vs.* normal; RA *vs.* normal	471.580	870.540	0.870	Phosphatidylglyceride 22:6-22:4	$C_{50}H_{79}O_{10}P$		1.591	0.057	1.219
Serum	cold *vs.* normal; RA *vs.* normal	745.820	881.527	0.310	Phosphatidylinositol 18:1-20:5	$C_{47}H_{79}O_{13}P$		4.555	0.011	2.018
synovial fluid	cold *vs.* normal	350.230	329.235	0.950	9,10,13-TriHOME|9,10,13-Trihydroxyoc	$C_{18}H_{34}O_5$	29907-57-1	0.185	0.014	1.105
synovial fluid	cold *vs.* hot	351.910	448.308	1.000	Glycodeoxycholic acid	$C_{26}H_{43}NO_5$	360-65-6	0.446	0.049	1.105
synovial fluid	RA *vs.* normal	352.050	464.303	0.970	Glycocholic acid	$C_{26}H_{43}NO_6$	475-31-0	2.734	0.085	1.385
synovial fluid	cold *vs.* normal; hot *vs.* normal; RA *vs.* normal	352.390	188.953	1.000	Bromobenzene-3,4-dihydrodiol|4-Bromo	$C_6H_7BrO_2$	82683-93-0	0.071	0.033	1.252
synovial fluid	RA *vs.* normal	352.660	465.251	0.980	Androsterone glucuronide|Androsterone	$C_{25}H_{38}O_8$	1852-43-3|3	2.484	0.060	1.240
synovial fluid	cold *vs.* hot; cold *vs.* normal; cold *vs.* normal; hot *vs.* normal	353.930	395.190	0.960	3β-Hydroxypregn-5-en-20-one sulfate|Pr	$C_{21}H_{32}O_5S$	1247-64-9	0.492	0.112	1.019
synovial fluid	cold *vs.* hot; cold *vs.* normal; cold *vs.* normal; hot *vs.* normal	378.530	581.242	0.700	Biliverdin	$C_{33}H_{34}N_4O_6$	114-25-0	0.065	0.007	1.188
synovial fluid	cold *vs.* normal	419.270	476.280	0.930	LysoPE(18:2(9Z,12Z)/0:0)|PE(18:2(9Z,1	$C_{23}H_{44}NO_7P$	85046-18-0	0.462	0.017	1.137

synovial fluid	cold *vs.* normal	471.450	502.294	0.910	lysoPE 20:3	$C_{25}H_{46}NO_7P$		2.079	0.004	1.323
synovial fluid	RA *vs.* normal	472.100	522.285	0.710	PS(18:1(9Z)/0:0)	$C_{24}H_{46}NO_9P$		2.663	0.127	1.027
synovial fluid	cold *vs.* normal	497.840	297.244	0.880	cis-9,10-Epoxystearic acid	$C_{18}H_{34}O_3$	2443-39-2	0.312	0.021	1.072
synovial fluid	hot *vs.* normal; RA *vs.* normal; cold *vs.* normal; hot *vs.* norma	158.640	172.992	0.700	Aryl sulfate|Phenol sulfate|Phenylsulfate|l	$C_6H_6O_4S$	937-34-8	2.500	0.120	1.004
synovial fluid	hot *vs.* normal; RA *vs.* normal; cold *vs.* normal; hot *vs.* norma	526.490	295.229	1.000	Vernolic acid|(9Z)-(12S,13R)-12,13-Epo:	$C_{18}H_{32}O_3$	503-07-1	0.424	0.143	1.052
synovial fluid	cold *vs.* hot	165.090	212.003	0.960	Indoxyl sulfate	$C_8H_7NO_4S$	2642-37-7	2.440	0.039	1.152
synovial fluid	cold *vs.* hot	178.500	263.105	0.900	Phenylacetyl-L-glutamine	$C_{13}H_{16}N_2O_4$	28047-15-6	3.012	0.032	1.191
synovial fluid	RA *vs.* normal	563.360	490.332	0.770	CerP 25:1	$C_{25}H_{50}NO_6P$		2.064	0.075	1.074
synovial fluid	hot *vs.* normal; RA *vs.* normal; cold *vs.* normal; hot *vs.* norma	565.000	297.244	0.710	3-Oxooctadecanoic acid|3-keto stearic ac	$C_{18}H_{34}O_3$		0.361	0.026	1.123
synovial fluid	hot *vs.* normal; RA *vs.* normal; cold *vs.* normal; hot *vs.* norma	577.490	277.218	1.000	(9Z,11E,13E)-Octadecatrienoic acid|(9Z,	$C_{18}H_{30}O_2$	544-72-9|50	0.172	0.002	1.289
synovial fluid	cold *vs.* normal	589.040	473.284	1.000	Pubescenol	$C_{28}H_{42}O_6$	90685-93-1	0.389	0.012	1.208
synovial fluid	cold *vs.* normal; hot *vs.* normal; cold *vs.* normal; hot *vs.* norm	594.040	253.218	1.000	Hypogeic acid|cis-7-Hexadecenoic Acid|I	$C_{16}H_{30}O_2$	2416-19-5	0.149	0.004	1.229
synovial fluid	cold *vs.* normal; hot *vs.* normal; cold *vs.* normal; hot *vs.* norm	594.980	354.296	0.630	Pristanoylglycine	$C_{21}H_{41}NO_3$		0.298	0.003	1.331
synovial fluid	hot *vs.* normal	599.220	407.154	0.660	Beclomethasone	$C_{22}H_{29}ClO_5$	4419-39-0	0.290	0.004	1.099
synovial fluid	RA *vs.* normal	605.000	494.361	0.970	lysoPE 20:0|PE(O-20:0/0:0)	$C_{25}H_{54}NO_6P$		2.097	0.075	1.139
synovial fluid	cold *vs.* hot	216.980	187.008	0.710	p-Tolyl Sulfate	$C_7H_8O_4S$	91978-69-7	4.715	0.033	1.188
synovial fluid	cold *vs.* normal; hot *vs.* normal; RA *vs.* normal	613.290	380.161	0.710	Azelastine|Azelastine (INN)|Optivar (TN	$C_{22}H_{24}ClN_3O$	58581-89-8	0.403	0.008	1.148
synovial fluid	cold *vs.* normal; hot *vs.* normal; RA *vs.* normal	613.310	279.234	1.000	β-Linoleic acid	$C_{18}H_{32}O_2$	60-33-3	0.203	0.024	1.105
synovial fluid	cold *vs.* normal; hot *vs.* normal; RA *vs.* normal	619.710	518.363	0.900	CerP 27:1	$C_{27}H_{54}NO_6P$		2.601	0.006	1.076
synovial fluid	cold *vs.* normal; hot *vs.* normal; RA *vs.* normal	637.280	255.234	0.610	Palmitic acid	$C_{16}H_{32}O_2$	######	0.244	0.015	1.154
synovial fluid	cold *vs.* normal; hot *vs.* normal; RA *vs.* normal	637.730	355.159	0.640	Rutamarin|Chalepin acetate	$C_{21}H_{24}O_5$	14882-94-1	0.466	0.001	1.382
synovial fluid	cold *vs.* normal; hot *vs.* normal; RA *vs.* normal	638.530	329.249	0.990	All-cis-4,7,10,13,16-docosapentaenoic ac	$C_{22}H_{34}O_2$	25182-74-5	0.415	0.082	1.025
synovial fluid	cold *vs.* normal; hot *vs.* normal; RA *vs.* normal	653.880	442.091	0.810	N-debutylhalofantrine	$C_{22}H_{22}Cl_2F_3NO$		0.307	0.006	1.215
synovial fluid	cold *vs.* normal; hot *vs.* normal; RA *vs.* normal	654.580	281.250	1.000	trans-9-Octadecenoic acid	$C_{18}H_{34}O_2$	112-79-8	0.117	0.049	1.051
synovial fluid	cold *vs.* normal; hot *vs.* normal; RA *vs.* normal	654.610	381.175	0.640	Corticosterone|11beta,21-Dihydroxy-4-pı	$C_{21}H_{30}O_4$	50-22-6	0.328	0.001	1.375
synovial fluid	cold *vs.* normal; hot *vs.* normal; RA *vs.* normal	656.950	331.265	1.000	FA 22:4	$C_{22}H_{36}O_2$		0.362	0.051	1.101
synovial fluid	cold *vs.* normal; hot *vs.* normal; RA *vs.* normal	678.160	546.395	0.940	CerP 29:1	$C_{29}H_{58}NO_6P$		2.288	0.009	1.042
synovial fluid	RA *vs.* normal	761.150	833.522	1.000	PI 34:2	$C_{43}H_{79}O_{13}P$		0.451	0.047	1.056
synovial fluid	RA *vs.* normal	827.560	739.514	0.760	Phosphatidylethanolamine 16:0-20:4|Pho	$C_{41}H_{74}NO_8P$		0.443	0.007	1.212
synovial fluid	cold *vs.* normal; RA *vs.* normal	829.770	580.496	0.780	Cer-NS d34:2; Cer-NS d19:1/15:1	$C_{34}H_{65}NO_3$		0.363	0.045	1.069
synovial fluid	cold *vs.* normal	844.100	774.542	0.710	PE 40:7e|PE 18:5e/22:2|EtherPE 40:7e|Pl	$C_{45}H_{78}NO_7P$		0.403	0.017	1.109
synovial fluid	cold *vs.* hot	294.040	383.154	0.950	19-hydroxy-17-oxoandrost-5-en-3-β-yl sı	$C_{19}H_{28}O_6S$		0.346	0.130	1.037
synovial fluid	cold *vs.* normal	865.780	814.597	1.000	HexCer-ADS d37:2|HexCer-ADS d19:0/	$C_{43}H_{81}NO_9$		0.495	0.021	1.071
synovial fluid	cold *vs.* normal; RA *vs.* normal	868.740	736.531	0.940	HexCer-AS d36:4; HexCer-AS d23:3/13:	$C_{42}H_{75}NO_9$		0.441	0.060	1.052

synovial fluid	cold *vs.* normal	884.030	764.584	0.940	Cer-BDS d46:9|Cer_BS d22:3/24:6|Cer[B	$C_{46}H_{75}NO_4$		3.051	0.020	1.082
synovial fluid	cold *vs.* hot	887.290	700.531	0.910	HexCer-AS d33:1|HexCer-AS d21:1/12:(	$C_{39}H_{75}NO_9$		2.666	0.059	1.061
synovial fluid	cold *vs.* normal; hot *vs.* normal	890.380	776.562	0.980	HexCer-AS d39:5|HexCer-AS d23:3/16:2	$C_{45}H_{79}NO_9$		0.364	0.017	1.115
synovial fluid	cold *vs.* normal	898.670	790.600	0.990	HexCer-ADS d35:0; HexCer-ADS d19:0.	$C_{41}H_{81}NO_9$		0.420	0.026	1.044
synovial fluid	cold *vs.* normal	899.950	838.600	0.900	HexCer-AS d39:4|HexCer-AS d23:2/16:2	$C_{45}H_{81}NO_9$		0.342	0.028	1.094
synovial fluid	cold *vs.* normal	302.110	367.108	1.000	Gibberellic acid	$C_{19}H_{22}O_6$	1977/6/5	0.133	0.012	1.106
synovial fluid	cold *vs.* normal	900.930	610.543	0.850	Cer 36:1	$C_{36}H_{71}NO_3$		0.248	0.027	1.034
synovial fluid	cold *vs.* normal	905.760	814.600	1.000	HexCer-AS d37:2; HexCer-AS d21:2/16:	$C_{43}H_{81}NO_9$		0.289	0.010	1.201
synovial fluid	cold *vs.* hot; cold *vs.* normal	912.750	816.615	0.990	HexCer-AS d37:1|HexCer-AS d21:1/16:(	$C_{43}H_{83}NO_9$		2.439	0.030	1.200
synovial fluid	cold *vs.* normal	912.760	817.645	0.810	SM d39:1|SM d21:0/18:1|SM(d21:0/18:1	$C_{44}H_{89}N_2O_6P$		2.303	0.005	1.238
synovial fluid	cold *vs.* normal	944.780	664.591	0.820	Cer 40:2	$C_{40}H_{77}NO_3$		0.171	0.005	1.239
synovial fluid	hot *vs.* normal	946.510	690.607	0.780	Cer 42:3	$C_{42}H_{79}NO_3$		0.365	0.007	1.034
synovial fluid	cold *vs.* normal	953.470	856.692	0.820	HexCer-NDS d42:1|HexCer-NDS d18:0/2	$C_{48}H_{93}NO_8$		0.440	0.005	1.198
synovial fluid	cold *vs.* hot; cold *vs.* normal; RA *vs.* normal; cold *vs.* hot	954.440	857.679	0.780	SM d42:2|SM d20:0/22:2|SM(d20:0/22:2	$C_{47}H_{93}N_2O_6P$		2.207	0.150	1.126
synovial fluid	cold *vs.* hot; cold *vs.* normal; RA *vs.* normal; cold *vs.* hot	962.670	831.663	0.770	SM d40:1|SM d24:1/16:0|SM(d24:1/16:0	$C_{45}H_{91}N_2O_6P$		2.906	0.072	1.206
synovial fluid	cold *vs.* hot; cold *vs.* normal; RA *vs.* normal; cold *vs.* hot	306.850	332.135	0.660	Pefloxacin (USAN/INN)|PFLX|Pefloxaci	$C_{17}H_{20}FN_3O_3$	70458-92-3	0.117	0.014	1.089
synovial fluid	RA *vs.* normal	309.310	528.265	0.900	Glycochenodeoxycholate 7-sulfate|N-[(3a	$C_{26}H_{43}NO_8S$	67030-55-1	0.398	0.116	1.502
synovial fluid	cold *vs.* hot; cold *vs.* normal; cold *vs.* hot; cold *vs.* normal	310.270	413.201	0.950	Eplerenone|Eplerenone (JP17/USAN/INN	$C_{24}H_{30}O_6$	107724-20-	0.443	0.055	1.178
synovial fluid	cold *vs.* hot; cold *vs.* normal; cold *vs.* hot; cold *vs.* normal	335.710	397.207	0.960	Methyl prednisolone acetate	$C_{24}H_{32}O_6$	53-36-1	0.460	0.104	1.071
synovial fluid	cold *vs.* hot	283.690	359.184	0.660	Prednisone	$C_{21}H_{26}O_5$	1953/3/2	2.867	0.060	1.140
synovial fluid	cold *vs.* normal; hot *vs.* normal	346.540	359.159	0.530	Matairesinol|(-)-Matairesinol|(3R,4R)-3,4	$C_{20}H_{22}O_6$	580-72-3	0.135	0.015	1.083
synovial fluid	hot *vs.* normal	348.120	236.162	1.000	Stovaine|Amylocaine|Amylocaine (BAN)	$C_{14}H_{21}NO_2$	644-26-8	0.414	0.003	1.132
synovial fluid	cold *vs.* normal; hot *vs.* normal; cold *vs.* hot	351.460	633.252	0.780	CandesartanCilextil			0.100	0.020	1.078
synovial fluid	cold *vs.* normal; hot *vs.* normal; cold *vs.* hot	355.310	585.268	0.790	4Z,15E-Bilirubin IXa	$C_{33}H_{36}N_4O_6$		2.127	0.025	1.238
synovial fluid	cold *vs.* normal; hot *vs.* normal; cold *vs.* hot	355.400	586.271	0.740	PS 20:2|PS 3:0-17:2|PS(3:0/17:2)	$C_{26}H_{46}NO_{10}P$		2.112	0.026	1.232
synovial fluid	cold *vs.* normal; hot *vs.* normal; cold *vs.* hot	373.530	633.253	0.780	CandesartanCilextil			0.099	0.011	1.124
synovial fluid	cold *vs.* normal; hot *vs.* normal; cold *vs.* hot	378.850	583.253	0.780	Biliverdin	$C_{33}H_{34}N_4O_6$	114-25-0	0.027	0.011	1.124
synovial fluid	hot *vs.* normal	380.800	649.247	0.600	Chalcomoracin|CHALCOMORACIN	$C_{39}H_{36}O_9$	76472-89-4	0.126	0.006	1.032
synovial fluid	cold *vs.* hot	417.080	274.273	0.670	Hexadecasphinganine|C16 Sphinganine	$C_{16}H_{35}NO_2$		0.453	0.049	1.103
synovial fluid	cold *vs.* hot	417.880	275.277	0.800	7,11,15-Trimethyl-3-methylenehexadeca-	$C_{20}H_{34}$		0.484	0.047	1.124
synovial fluid	RA *vs.* normal	486.360	440.311	0.790	lysoPE 16:0|PE(O-16:0/0:0)	$C_{21}H_{46}NO_6P$		2.066	0.028	1.214
synovial fluid	RA *vs.* normal	118.950	205.028	0.820	Oxaloglutarate	$C_7H_8O_7$		2.475	0.007	1.295
synovial fluid	cold *vs.* hot	524.020	214.179	0.800	N-(9-Oxodecyl)acetamide	$C_{12}H_{23}NO_2$		2.480	0.019	1.289
synovial fluid	cold *vs.* hot	524.290	230.174	0.900	2-(6-hydroxy-6-methylheptyl)-2H-furan-5	$C_{12}H_{20}O_3$		2.430	0.022	1.264
synovial fluid	RA *vs.* normal	524.650	490.327	0.540	Oxysporidinone		184871-55-	2.001	0.051	1.189

synovial fluid	cold *vs.* normal; hot *vs.* normal; RA *vs.* normal	532.980	278.247	0.750	Obscuraminol A	$C_{18}H_{31}NO$		0.310	0.083	1.177
synovial fluid	cold *vs.* hot	534.410	172.132	0.550	Gabapentin (Neurontin)		60142-96-3	2.294	0.067	1.030
synovial fluid	hot *vs.* normal	540.580	293.246	1.000	5α-Androstane-11β,17β-diol	$C_{19}H_{32}O_2$		0.079	0.007	1.034
synovial fluid	RA *vs.* normal	543.370	468.342	0.690	lysoPE 18:0|PE(O-18:0/0:0)	$C_{23}H_{50}NO_6P$		2.073	0.043	1.149
synovial fluid	hot *vs.* normal	547.960	636.420	0.590	SM d28:3|SM d14:3/14:0|SM(d14:3/14:0	$C_{33}H_{63}N_2O_6P$		0.393	0.004	1.056
synovial fluid	hot *vs.* normal	566.280	651.439	0.710	Progesterone			0.388	0.005	1.067
synovial fluid	hot *vs.* normal	566.310	650.436	0.990	PC(16:0/9:0(CHO))	$C_{33}H_{64}NO_9P$		0.388	0.003	1.105
synovial fluid	hot *vs.* normal; RA *vs.* normal	569.700	424.340	0.870	α-Tocotrienoxyl radical	$C_{29}H_{43}O_2$		0.252	0.028	1.094
synovial fluid	hot *vs.* normal	570.990	242.283	1.000	1-Hexadecylamine	$C_{16}H_{35}N$	143-27-1	2.409	0.000	1.253
synovial fluid	cold *vs.* normal	580.100	279.230	0.710	Pinolenic Acid|Pinolenic acid	$C_{18}H_{30}O_2$	16833-54-8	0.431	0.032	1.001
synovial fluid	cold *vs.* hot	585.170	200.163	0.960	11-nitro-1-undecene	$C_{11}H_{21}NO_2$		2.086	0.065	1.054
synovial fluid	RA *vs.* normal	589.030	552.400	0.950	PC(O-18:0/2:0)	$C_{28}H_{58}NO_7P$		0.146	0.106	1.270
synovial fluid	RA *vs.* normal	595.670	400.340	0.680	L-Palmitoylcarnitine|Palmitoylcarnitine|P	$C_{23}H_{45}NO_4$	1935-18-8|2	0.312	0.051	1.404
synovial fluid	RA *vs.* normal	598.350	401.344	0.740	7-Ketocholesterol	$C_{27}H_{44}O_2$	566-28-9	0.270	0.052	1.470
synovial fluid	RA *vs.* normal	603.020	583.252	0.660	Biliverdin	$C_{33}H_{34}N_4O_6$		0.415	0.017	1.014
synovial fluid	cold *vs.* normal; hot *vs.* normal	612.400	263.236	1.000	Farnesyl acetone	$C_{18}H_{30}O$		0.337	0.003	1.254
synovial fluid	cold *vs.* normal; hot *vs.* normal	612.400	281.246	0.700	β-Linoleic acid	$C_{18}H_{32}O_2$	60-33-3	0.328	0.004	1.253
synovial fluid	hot *vs.* normal	619.360	520.374	0.560	PC(P-19:1(12Z)/0:0)	$C_{27}H_{54}NO_6P$		2.916	0.007	1.067
synovial fluid	hot *vs.* normal	621.650	678.467	0.770	PS(P-16:0/13:0)	$C_{35}H_{68}NO_9P$		0.439	0.004	1.062
synovial fluid	hot *vs.* normal; RA *vs.* normal; cold *vs.* normal	626.960	426.356	0.790	MAG 22:3	$C_{25}H_{44}O_4$		0.101	0.037	1.062
synovial fluid	hot *vs.* normal; RA *vs.* normal; cold *vs.* normal	635.940	585.268	0.730	Bilirubin|4E,15Z-Bilirubin IXa	$C_{33}H_{36}N_4O_6$	635-65-4|69	0.054	0.026	1.053
synovial fluid	cold *vs.* normal	636.030	583.252	0.970	Okaramine D		162413-54-	0.095	0.014	1.107
synovial fluid	RA *vs.* normal	134.390	208.132	0.580	3,4-Methylenedioxy-N-ethylamphetamin	$C_{12}H_{17}NO_2$	14089-52-2	9.421	0.030	1.515
synovial fluid	RA *vs.* normal	658.770	449.360	0.720	Typhasterol|2-Deoxycastasterone	$C_{28}H_{48}O_4$	87734-68-7	2.391	0.105	1.126
synovial fluid	hot *vs.* normal	677.300	548.405	0.610	CerP 29:1	$C_{29}H_{58}NO_6P$		2.835	0.002	1.155
synovial fluid	hot *vs.* normal	699.040	512.502	0.790	Cer-NDS d32:0|Cer-NDS d15:0/17:0|Cer	$C_{32}H_{65}NO_3$		0.047	0.011	1.136
synovial fluid	cold *vs.* hot; hot *vs.* normal	707.990	862.622	0.680	Galabiosylceramide (d18:1/16:0)	$C_{46}H_{87}NO_{13}$		2.031	0.000	1.795
synovial fluid	RA *vs.* normal	708.430	617.472	0.940	SM d28:2|SM d15:2/13:0|SM(d15:2/13:0	$C_{33}H_{65}N_2O_6P$		2.241	0.235	1.155
synovial fluid	cold *vs.* normal; hot *vs.* normal; RA *vs.* normal	732.590	325.309	0.830	4,8,12,16-Tetramethylheptadecan-4-olide	$C_{21}H_{40}O_2$		0.327	0.038	1.050
synovial fluid	cold *vs.* hot	746.450	835.530	0.900	PI(12:0/22:2(13Z,16Z))	$C_{43}H_{79}O_{13}P$		2.043	0.020	1.287
synovial fluid	hot *vs.* normal	755.960	773.614	0.680	PA(21:0/20:1(11Z))	$C_{44}H_{85}O_8P$		0.444	0.006	1.043
synovial fluid	cold *vs.* normal	834.190	720.551	0.980	PC(12:0/19:0)	$C_{39}H_{78}NO_8P$		2.013	0.019	1.073
synovial fluid	cold *vs.* hot	856.110	796.582	0.970	PC(17:1(9Z)/20:3(8Z,11Z,14Z))	$C_{45}H_{82}NO_8P$		2.439	0.076	1.094
synovial fluid	cold *vs.* normal	861.820	431.383	0.940	α-Tocopherol|Vitamin E|Tocopherol (JP1	$C_{29}H_{50}O_2$	59-02-9|101	2.888	0.002	1.454
synovial fluid	RA *vs.* normal	862.030	538.517	0.980	Ceramide (18:1/16:0)			0.460	0.211	1.259

synovial fluid	cold *vs.* hot	904.750	730.568	0.580	plasmenyl-PE 36:1	$C_{41}H_{80}NO_7P$		2.272	0.078	1.018
synovial fluid	cold *vs.* normal	911.490	748.609	0.710	DGTS 35:3|DGTS 14:1-21:2|DGTS(14:1	$C_{45}H_{81}NO_7$		2.178	0.007	1.182
synovial fluid	cold *vs.* hot	920.410	575.500	0.930	Montecristin	$C_{37}H_{66}O_4$	185336-15-	4.163	0.022	1.315
synovial fluid	cold *vs.* hot; cold *vs.* normal; hot *vs.* normal	967.810	835.663	0.880	SM 42:2	$C_{47}H_{93}N_2O_6P$		2.315	0.024	1.470
synovial fluid	cold *vs.* hot; cold *vs.* normal; hot *vs.* normal	1075.150	533.524	1.000	Stearyl linoleate	$C_{36}H_{68}O_2$		0.126	0.001	1.384
synovial fluid	RA *vs.* normal	1090.440	815.696	1.000	SM(d18:1/24:0)	$C_{47}H_{95}N_2O_6P$		0.413	0.221	1.198
synovial fluid	cold *vs.* normal; hot *vs.* normal	250.780	172.133	1.000	Gabapentin (JAN/USAN/INN)|Neurontin	$C_9H_{17}NO_2$	60142-96-3	0.427	0.012	1.131
Serum	cold *vs.* hot; cold *vs.* normal; hot *vs.* normal; RA *vs.* normal	100.580	146.985	1.000	3-Acetyl-1,2-dithiolane	$C_5H_8OS_2$	89712-89-0	1.771	0.073	1.709
Serum	cold *vs.* hot	747.090	778.595	0.920	Cer-BDS d47:9|Cer_BS d20:2/27:7|Cer[I	$C_{47}H_{77}NO_4$		0.739	0.526	1.024
Serum	cold *vs.* normal	812.940	862.561	0.920	Phosphatidylcholine 16:0-22:7	$C_{47}H_{80}NO_8P$		2.598	0.017	1.978
Serum	cold *vs.* normal	752.080	835.527	0.900	Roxithromycin	$C_{41}H_{76}N_2O_{15}$	80214-83-1	2.210	0.139	1.293
Serum	hot *vs.* normal; RA *vs.* normal	471.580	870.540	0.870	Phosphatidylglyceride 22:6-22:4	$C_{50}H_{79}O_{10}P$		1.591	0.057	1.219
Serum	cold *vs.* normal	614.070	479.342	0.870	24-epi-Brassinolide|Brassinolide|2,4-Epit	$C_{28}H_{48}O_6$	78821-43-9	2.169	0.141	1.299
Serum	cold *vs.* hot	372.000	375.197	1.000	Fluoromethalone	$C_{22}H_{29}FO_4$	426-13-1	1.753	0.322	1.007
Serum	cold *vs.* hot	569.090	415.226	0.700	Ramipril (USP/INN)|Altace (TN)|Ramipr	$C_{23}H_{32}N_2O_5$	87333-19-5	0.716	0.060	1.648
Serum	cold *vs.* normal	848.710	790.593	0.670	Cer-BDS d48:10|Cer_BS d21:3/27:7|Cer|	$C_{48}H_{77}NO_4$		0.507	0.314	1.002
Serum	cold *vs.* hot; RA *vs.* normal; hot *vs.* normal	636.580	371.223	0.650	5-Ethoxysorgoleone 358	$C_{23}H_{32}O_4$	210708-13-	0.489	0.271	1.186
Serum	cold *vs.* hot; RA *vs.* normal; hot *vs.* normal	582.720	417.317	0.580	β-Citraurinene	$C_{30}H_{42}O$	58947-97-0	1.587	0.100	1.026
Serum	cold *vs.* hot	397.720	267.131	0.570	3-carboxy-4-methyl-5-pentyl-2-furanprop	$C_{14}H_{20}O_5$		2.477	0.076	1.594
Serum	cold *vs.* normal; hot *vs.* normal; RA *vs.* normal	441.980	507.277	1.000	LPG 18:2	$C_{24}H_{45}O_9P$		1.644	0.187	1.201
Serum	cold *vs.* normal	862.080	572.501	1.000	Cer-NS d37:4|Cer-NS d24:3/13:1|Cer[NS	$C_{37}H_{67}NO_3$		0.543	0.344	1.150
Serum	cold *vs.* hot	926.480	858.630	1.000	HexCer-AP t39:2|HexCer-AP t20:1/19:1|	$C_{45}H_{85}NO_{10}$		0.204	0.272	1.082
Serum	cold *vs.* normal	196.510	129.057	0.980	4-Methyl-2-Oxovalerate	$C_6H_{10}O_3$	4502-00-5	1.743	0.262	1.040
Serum	cold *vs.* hot; hot *vs.* normal; RA *vs.* normal; cold *vs.* normal	945.690	772.618	1.000	PC(P-16:0/20:1(11Z))	$C_{44}H_{86}NO_7P$		0.570	0.276	1.085
Serum	cold *vs.* hot; hot *vs.* normal; RA *vs.* normal; cold *vs.* normal	523.340	666.432	1.000	PC(16:0/9:0(COOH))	$C_{33}H_{64}NO_{10}P$		1.658	0.115	1.435
Serum	RA *vs.* normal	409.450	398.240	1.000	S-4-benzyl-3-((S)-3-hydroxy-2,2-dimethyloctanoyl)-5,5-dimethyloxazolidin-			1.227	0.166	1.054
Serum	cold *vs.* hot	658.670	360.317	1.000	5-Decanoyl-2-nonylpyridine	$C_{24}H_{41}NO$	149682-94-	0.261	0.313	1.123
Serum	cold *vs.* normal	859.310	788.555	1.000	plasmenyl-PC 36:4	$C_{44}H_{80}NO_7P$		0.528	0.227	1.213
Serum	cold *vs.* hot	130.310	185.128	1.000	N-(3-acetamidopropyl)pyrrolidin-2-one|N	$C_9H_{16}N_2O_2$	106692-36-	1.515	0.284	1.031
Serum	cold *vs.* hot; cold *vs.* normal; hot *vs.* normal; RA *vs.* normal	86.740	175.118	0.990	L-Arginine	$C_6H_{14}N_4O_2$	74-79-3	0.708	0.261	1.121
Serum	cold *vs.* hot; cold *vs.* normal; hot *vs.* normal; RA *vs.* normal	566.510	650.437	0.990	PC(16:0/9:0(CHO))	$C_{33}H_{64}NO_9P$		1.428	0.122	1.426
Serum	cold *vs.* hot; cold *vs.* normal; hot *vs.* normal; RA *vs.* normal	516.620	496.374	0.990	PC(O-16:0/O-1:0)	$C_{25}H_{54}NO_6P$		1.393	0.457	1.024
Serum	RA *vs.* normal	924.590	794.604	0.990	PC(O-18:1(9Z)/20:4(5Z,8Z,11Z,14Z))	$C_{46}H_{84}NO_7P$		0.699	0.032	1.431
Serum	cold *vs.* normal	896.470	718.573	0.990	PC(P-16:0/16:0)	$C_{40}H_{80}NO_7P$		0.292	0.081	1.543

Serum	hot *vs.* normal; RA *vs.* normal	684.690	324.325	0.990	N-(14-Methylhexadecanoyl)pyrrolidine	$C_{21}H_{41}NO$	260058-83-	1.342	0.117	1.071
Serum	cold *vs.* hot	599.310	228.231	1.000	N-Tetradecanamide	$C_{14}H_{29}NO$	638-58-4	1.519	0.555	1.042
Serum	cold *vs.* normal	577.720	279.231	0.990	α-Linolenic acid	$C_{18}H_{30}O_2$	463-40-1	2.219	0.043	1.711
Serum	cold *vs.* normal	903.790	720.588	0.990	PC(O-18:0/14:0)	$C_{40}H_{82}NO_7P$		0.591	0.248	1.114
Serum	cold *vs.* hot; hot *vs.* normal; RA *vs.* normal	606.810	538.421	0.990	PC(O-16:0/O-4:0)[U]	$C_{28}H_{60}NO_6P$		1.411	0.348	1.111
Serum	cold *vs.* normal	122.590	132.102	0.990	D-Norleucine	$C_6H_{13}NO_2$	327-56-0	1.242	0.228	1.112
Serum	cold *vs.* normal; hot *vs.* normal; RA *vs.* normal; cold *vs.* hot	841.510	692.557	0.990	PC(o-14:0/16:0)|PC(O-14:0/16:0)	$C_{38}H_{78}NO_7P$		0.392	0.128	1.378
Serum	cold *vs.* normal; hot *vs.* normal; RA *vs.* normal; cold *vs.* hot	428.520	230.247	1.000	3-epi-xestoaminol C			0.607	0.184	1.266
Serum	RA *vs.* normal	952.800	848.650	0.990	PC(O-20:0/22:6(4Z,7Z,10Z,13Z,16Z,19Z	$C_{50}H_{90}NO_7P$		1.681	0.020	1.480
Serum	cold *vs.* hot	1227.490	879.738	0.990	SM 45:1	$C_{50}H_{101}N_2O_6P$		0.208	0.240	1.173
Serum	cold *vs.* normal	505.180	572.370	0.990	LysoPC(22:4(7Z,10Z,13Z,16Z))|PC(22:4	$C_{30}H_{54}NO_7P$		1.506	0.001	2.380
Serum	cold *vs.* hot	884.770	836.613	0.990	PC(22:1(11Z)/18:4(6Z,9Z,12Z,15Z))	$C_{48}H_{86}NO_8P$		1.406	0.250	1.431
Serum	cold *vs.* hot	348.160	236.162	1.000	Stovaine|Amylocaine|Amylocaine (BAN)	$C_{14}H_{21}NO_2$	644-26-8	0.441	0.198	1.214
Serum	cold *vs.* normal; hot *vs.* normal; RA *vs.* normal	860.920	431.385	0.990	α-Tocopherol|Vitamin E|Tocopherol (JP1	$C_{29}H_{50}O_2$	59-02-9|101	0.482	0.070	1.593
Serum	cold *vs.* normal	482.620	568.336	0.990	PC(22:6(4E,7E,10E,13E,16E,19E)/0:0)[U	$C_{30}H_{50}NO_7P$		1.204	0.268	1.122
Serum	cold *vs.* hot; hot *vs.* normal; cold *vs.* hot; RA *vs.* normal	284.710	414.204	0.980	(2S)-1-[6-[(10aS)-1,3-dioxo-10,10a-dihydro-5H-imidazo[1,5-b]isoquinolin-2			2.158	0.343	1.030
Serum	cold *vs.* hot; hot *vs.* normal; cold *vs.* hot; RA *vs.* normal	744.190	391.283	0.980	Diisooctyl phthalate	$C_{24}H_{38}O_4$	27554-26-3	0.687	0.331	1.088
Serum	cold *vs.* hot	954.190	814.631	0.980	PC(19:1(10Z)/19:1(10Z))[U]	$C_{46}H_{88}NO_8P$		0.267	0.262	1.087
Serum	cold *vs.* hot; hot *vs.* normal; RA *vs.* normal	906.790	774.598	0.980	PC(13:0/22:1(11Z))	$C_{43}H_{84}NO_8P$		0.504	0.266	1.069
Serum	cold *vs.* hot; hot *vs.* normal; RA *vs.* normal	943.990	796.620	0.980	PC(20:2(11Z,14Z)/P-18:1(11Z))|PC(20:2	$C_{46}H_{86}NO_7P$		0.738	0.378	1.071
Serum	cold *vs.* hot	1246.210	905.755	0.980	SM 47:2	$C_{52}H_{103}N_2O_6P$		0.333	0.175	1.253
Serum	cold *vs.* hot; cold *vs.* normal; RA *vs.* normal; hot *vs.* normal	588.500	242.284	1.000	1-Hexadecylamine	$C_{16}H_{35}N$	143-27-1	0.511	0.078	1.594
Serum	cold *vs.* hot; cold *vs.* normal; RA *vs.* normal; hot *vs.* normal	585.780	507.272	0.980	(22E)-26,26,26,27,27,27-hexafluoro-25-I	$C_{27}H_{36}F_6O_2$		0.216	0.030	1.840
Serum	cold *vs.* hot; cold *vs.* normal; RA *vs.* normal; hot *vs.* normal	860.440	818.604	0.980	PC(P-18:1(9Z)/22:5(4Z,7Z,10Z,13Z,16Z	$C_{48}H_{84}NO_7P$		1.281	0.151	1.507
Serum	cold *vs.* hot; cold *vs.* normal; RA *vs.* normal; hot *vs.* normal	910.360	796.618	0.980	PC(O-16:0/22:4(7Z,10Z,13Z,16Z))	$C_{46}H_{86}NO_7P$		0.620	0.315	1.030
Serum	cold *vs.* hot; cold *vs.* normal; RA *vs.* normal; hot *vs.* normal	424.440	415.210	0.980	6-[3-[(3,4-dimethoxyphenyl)methyl]-4-m	$C_{24}H_{32}O_7$		0.843	0.071	1.595
Serum	cold *vs.* hot; cold *vs.* normal; RA *vs.* normal; hot *vs.* normal	798.460	756.553	0.970	PC 32:0|PC(10:0/22:0)	$C_{40}H_{80}NO_8P$		1.531	0.240	1.056
Serum	cold *vs.* hot; cold *vs.* normal; RA *vs.* normal; hot *vs.* normal	679.410	590.452	0.970	CerP(d18:1/14:0)	$C_{32}H_{64}NO_6P$		0.613	0.039	1.316
Serum	cold *vs.* hot; cold *vs.* normal; RA *vs.* normal; hot *vs.* normal	958.140	788.622	0.970	PC(14:0/22:1(11Z))	$C_{44}H_{86}NO_8P$		1.875	0.033	1.295
Serum	cold *vs.* hot	724.240	604.566	0.970	Cer-NS d39:3|Cer-NS d21:3/18:0|Cer[NS	$C_{39}H_{73}NO_3$		0.664	0.376	1.043
Serum	cold *vs.* hot; cold *vs.* normal	622.780	331.262	0.970	All-cis-4,7,10,13,16-docosapentaenoic ac	$C_{22}H_{34}O_2$	25182-74-5	2.136	0.080	1.581
Serum	cold *vs.* hot	406.980	415.210	0.970	2H-Oxireno[1,10a]phenanthro[3,2-b]fura	$C_{24}H_{32}O_7$		0.511	0.285	1.037
Serum	cold *vs.* hot; cold *vs.* normal	357.240	246.152	1.000	Tropacocaine|Benzoyltropein|Benzoyltro	$C_{15}H_{19}NO_2$	537-26-8|19	0.458	0.024	2.046
Serum	cold *vs.* normal	788.770	780.552	0.970	PC 34:2	$C_{42}H_{80}NO_8P$		2.464	0.154	1.253
Serum	cold *vs.* normal	786.290	754.536	0.970	PC 32:1	$C_{40}H_{78}NO_8P$		1.961	0.023	1.861

Serum	cold *vs.* hot	812.100	744.553	0.970	PC(13:0/20:2(11Z,14Z))	$C_{41}H_{78}NO_8P$		1.540	0.255	1.210
Serum	cold *vs.* hot; cold *vs.* normal	842.080	742.537	0.960	PE 36:3	$C_{41}H_{76}NO_8P$		0.575	0.218	1.167
Serum	hot *vs.* normal	520.450	494.359	0.960	PC(P-17:0/0:0)	$C_{25}H_{52}NO_6P$		0.717	0.150	1.016
Serum	cold *vs.* hot	370.250	250.177	1.000	Alprenolol	$C_{15}H_{23}NO_2$	13655-52-2	0.789	0.169	1.276
Serum	cold *vs.* normal	629.790	383.315	0.960	MG(0:0/20:2(11Z,14Z)/0:0)	$C_{23}H_{42}O_4$		0.649	0.263	1.009
Serum	cold *vs.* normal; hot *vs.* normal; RA *vs.* normal	936.430	838.630	0.960	PC(22:1(11Z)/18:3(6Z,9Z,12Z))	$C_{48}H_{88}NO_8P$		1.970	0.229	1.118
Serum	cold *vs.* normal; hot *vs.* normal; RA *vs.* normal	792.570	854.567	0.960	PC(20:4(5Z,8Z,11Z,14Z)/22:6(4Z,7Z,10	$C_{50}H_{80}NO_8P$		1.689	0.055	1.305
Serum	cold *vs.* normal	852.960	806.565	0.960	PC(20:4(5Z,8Z,11Z,14Z)/18:2(9Z,12Z))[	$C_{46}H_{80}NO_8P$		0.365	0.244	1.165
Serum	cold *vs.* hot; hot *vs.* normal; RA *vs.* normal	637.010	588.437	0.960	CerP 32:2	$C_{32}H_{62}NO_6P$		1.485	0.357	1.063
Serum	cold *vs.* hot; hot *vs.* normal; RA *vs.* normal	546.530	532.372	0.960	lysoPC 18:0	$C_{26}H_{56}NO_6P$		0.745	0.122	1.161
Serum	cold *vs.* normal	540.950	550.385	0.960	PC(O-18:1(10E)/2:0)	$C_{28}H_{56}NO_7P$		1.216	0.102	1.479
Serum	cold *vs.* normal; hot *vs.* normal; RA *vs.* normal	898.900	818.604	0.960	PC(P-18:1(11Z)/22:5(4Z,7Z,10Z,13Z,16	$C_{48}H_{84}NO_7P$		0.593	0.193	1.167
Serum	cold *vs.* normal; hot *vs.* normal; RA *vs.* normal	393.460	409.160	0.960	Amlodipine	$C_{20}H_{25}ClN_2O_5$	88150-42-9	1.274	0.100	1.145
Serum	cold *vs.* normal	790.680	279.231	0.960	γ-Linolenic acid	$C_{18}H_{30}O_2$	506-26-3	1.793	0.117	1.537
Serum	hot *vs.* normal	836.440	790.571	0.950	PC(P-16:0/22:6(4Z,7Z,10Z,13Z,16Z,19Z	$C_{46}H_{80}NO_7P$		0.512	0.002	1.800
Serum	cold *vs.* hot	1187.900	903.739	0.950	TAG 54:5|TAG 15:0-19:3-20:2|TG 54:5|	$C_{57}H_{100}O_6$		0.500	0.410	1.051
Serum	hot *vs.* normal; RA *vs.* normal; cold *vs.* hot; cold *vs.* normal	386.040	343.295	0.950	Cocamidopropylβine|Cocamidoprpylβine	$C_{19}H_{38}N_2O_3$		1.224	0.055	1.242
Serum	hot *vs.* normal; RA *vs.* normal; cold *vs.* hot; cold *vs.* normal	1082.290	837.677	0.950	SM 42:1	$C_{47}H_{95}N_2O_6P$		0.442	0.283	1.271
Serum	hot *vs.* normal; RA *vs.* normal; cold *vs.* hot; cold *vs.* normal	591.430	331.284	0.950	Glycerol 1-hexadecanoate	$C_{19}H_{38}O_4$	542-44-9	0.317	0.333	1.083
Serum	hot *vs.* normal; RA *vs.* normal; cold *vs.* hot; cold *vs.* normal	926.780	824.617	0.950	PC(22:2(13Z,16Z)/17:2(9Z,12Z))	$C_{47}H_{86}NO_8P$		0.588	0.007	1.713
Serum	cold *vs.* hot	176.830	265.118	1.000	N-γ-Acetyl-N-2-Formyl-5-methoxykynur	$C_{13}H_{16}N_2O_4$	52450-38-1	3.910	0.002	2.367
Serum	cold *vs.* hot; cold *vs.* normal	586.530	523.244	0.950	Trilobolide	$C_{27}H_{38}O_{10}$	50657-07-3	0.291	0.068	1.634
Serum	cold *vs.* normal	832.220	804.550	0.940	PC 36:4	$C_{44}H_{80}NO_8P$		0.677	0.312	1.159
Serum	cold *vs.* normal; hot *vs.* normal; RA *vs.* normal	863.100	742.573	0.940	PC(16:1(9Z)/P-18:1(11Z))|PC(16:1(9Z)/	$C_{42}H_{80}NO_7P$		0.569	0.040	1.728
Serum	cold *vs.* normal; hot *vs.* normal; RA *vs.* normal	930.510	746.603	0.940	1-O-Hexadecyl-2-(9Z-octadecenoyl)-sn-	$C_{42}H_{85}NO_7P$		0.392	0.037	1.731
Serum	cold *vs.* normal	1125.020	837.677	0.940	SM 42:1	$C_{47}H_{95}N_2O_6P$		2.414	0.093	1.466
Serum	cold *vs.* normal	899.100	794.603	0.940	PC(P-18:1(9Z)/20:3(8Z,11Z,14Z))	$C_{46}H_{84}NO_7P$		0.537	0.295	1.013
Serum	cold *vs.* hot; cold *vs.* normal	820.020	806.568	0.940	PC 36:3	$C_{44}H_{82}NO_8P$		1.269	0.324	1.238
Serum	cold *vs.* hot	645.660	561.410	0.940	SM 24:2	$C_{29}H_{57}N_2O_6P$		1.466	0.312	1.119
Serum	cold *vs.* normal	504.350	548.369	0.930	PC(20:2(11Z,14Z)/0:0)	$C_{28}H_{54}NO_7P$		1.224	0.281	1.006
Serum	cold *vs.* hot	725.580	321.278	0.930	Cis-8,11,14-Eicosatrienoic acid Methyl e	$C_{21}H_{36}O_2$	21061-10-9	1.370	0.445	1.022
Serum	hot *vs.* normal; RA *vs.* normal	492.680	502.325	0.930	lysoPC 16:0	$C_{24}H_{50}NO_6P$		0.616	0.037	1.277
Serum	cold *vs.* hot	737.110	282.278	1.000	Oleamide	$C_{18}H_{35}NO$	301-02-0	0.370	0.215	1.210
Serum	cold *vs.* normal	501.750	510.354	0.930	PC(16:0/O-1:0)	$C_{25}H_{52}NO_7P$		1.362	0.075	1.530

Serum	hot *vs.* normal	864.370	772.584	0.930	PC(13:0/22:2(13Z,16Z))	$C_{43}H_{82}NO_8P$		0.773	0.168	1.007
Serum	cold *vs.* normal; RA *vs.* normal	819.130	828.549	0.930	PC 38:6	$C_{46}H_{80}NO_8P$		0.662	0.227	1.190
Serum	cold *vs.* hot	847.260	808.583	0.930	PC 36:2	$C_{44}H_{84}NO_8P$		1.199	0.533	1.159
Serum	cold *vs.* hot	673.840	284.294	1.000	Stearamide	$C_{18}H_{37}NO$	124-26-5	0.120	0.008	2.093
Serum	cold *vs.* normal; hot *vs.* normal; RA *vs.* normal	863.990	764.556	0.920	plasmenyl-PC 34:2	$C_{42}H_{80}NO_7P$		0.328	0.029	1.815
Serum	cold *vs.* normal; hot *vs.* normal; RA *vs.* normal	931.680	770.603	0.920	PC(O-16:0/20:3(8Z,11Z,14Z))	$C_{44}H_{84}NO_7P$		0.468	0.017	1.430
Serum	cold *vs.* normal; hot *vs.* normal; RA *vs.* normal	392.050	223.063	0.920	Fraxidin|8-Hydroxy-6,7-dimethoxy-2H-1	$C_{11}H_{10}O_5$	525-21-3	1.872	0.036	1.293
Serum	cold *vs.* normal	462.030	526.291	0.920	lysoPE 20:3	$C_{25}H_{46}NO_7P$		1.295	0.293	1.004
Serum	hot *vs.* normal; RA *vs.* normal	553.890	348.285	0.920	O-Arachidonoyl Ethanolamine|Virodham	$C_{22}H_{37}NO_2$	443129-35-	1.687	0.044	1.243
Serum	cold *vs.* normal	761.460	678.505	0.920	PC(13:0/15:0)	$C_{36}H_{72}NO_8P$		1.903	0.154	1.249
Serum	cold *vs.* hot; RA *vs.* normal; cold *vs.* normal	1246.930	900.799	0.920	Triacylglycerol 18:1-18:1-18:2	$C_{57}H_{102}O_6$		0.273	0.261	1.072
Serum	cold *vs.* hot; RA *vs.* normal; cold *vs.* normal	540.130	349.273	0.910	17β-Hydroxy-2α-(methoxymethyl)-17-m	$C_{22}H_{36}O_3$		1.440	0.222	1.193
Serum	cold *vs.* hot	827.440	770.566	0.910	PC(16:0/19:3(9Z,12Z,15Z))[U]	$C_{43}H_{80}NO_8P$		1.227	0.460	1.058
Serum	cold *vs.* normal	575.030	358.294	0.910	N-palmitoyl threonine	$C_{20}H_{39}NO_4$		1.626	0.130	1.403
Serum	cold *vs.* hot; RA *vs.* normal	580.430	560.405	0.910	lysoDGTS 23:6	$C_{33}H_{53}NO_6$		1.576	0.357	1.010
Serum	cold *vs.* hot	938.450	823.639	0.910	18:3-Glc-Campesterol	$C_{52}H_{86}O_7$		0.688	0.329	1.023
Serum	RA *vs.* normal	944.490	577.517	0.910	Cohibin C	$C_{37}H_{68}O_4$		1.687	0.163	1.117
Serum	cold *vs.* hot	900.840	838.621	0.910	PC 38:1	$C_{46}H_{90}NO_8P$		1.562	0.515	1.046
Serum	hot *vs.* normal; RA *vs.* normal; cold *vs.* hot	746.760	297.278	0.910	Methyl oleate	$C_{19}H_{36}O_2$	112-62-9	0.764	0.073	1.147
Serum	hot *vs.* normal; RA *vs.* normal; cold *vs.* hot	669.860	298.310	0.910	Tridemorph|2,6-Dimethyl-4-tridecylmorp	$C_{19}H_{39}NO$	81412-43-3	0.491	0.009	2.132
Serum	hot *vs.* normal	697.640	615.457	0.900	SM d28:3|SM d14:3/14:0|SM(d14:3/14:0	$C_{33}H_{63}N_2O_6P$		1.495	0.122	1.096
Serum	cold *vs.* hot	686.980	293.225	1.000	3β-Fluoroandrost-5-en-17β-ol	$C_{19}H_{29}FO$		1.413	0.378	1.121
Serum	cold *vs.* hot; cold *vs.* normal	319.340	363.216	0.900	Hydrocortisone	$C_{21}H_{30}O_5$	50-23-7	2.461	0.039	1.767
Serum	hot *vs.* normal	302.800	391.229	0.900	Hexa(methoxymethyl)melamine|2-N,2-N,	$C_{15}H_{30}N_6O_6$	3089-11-0	0.464	0.103	1.148
Serum	cold *vs.* normal; RA *vs.* normal	863.410	718.571	0.900	PC(P-20:0/12:0)	$C_{40}H_{80}NO_7P$		0.574	0.078	1.521
Serum	cold *vs.* hot	423.660	432.236	0.900	7b,9-Dihydroxy-3-(hydroxymethyl)-1,1,6	$C_{22}H_{30}O_6$		0.781	0.364	1.001
Serum	cold *vs.* hot; cold *vs.* normal; hot *vs.* normal; RA *vs.* normal	630.680	270.279	0.900	Capsi-amide|N-(13-Methyltetradecyl)ace	$C_{17}H_{35}NO$	64317-66-4	0.462	0.164	1.343
Serum	cold *vs.* hot; cold *vs.* normal; hot *vs.* normal; RA *vs.* normal	830.200	740.521	0.900	PE 36:4	$C_{41}H_{74}NO_8P$		0.496	0.083	1.191
Serum	cold *vs.* hot; cold *vs.* normal; hot *vs.* normal; RA *vs.* normal	807.180	740.522	0.900	PE 36:4	$C_{41}H_{74}NO_8P$		0.406	0.141	1.362
Serum	cold *vs.* hot; cold *vs.* normal; hot *vs.* normal; RA *vs.* normal	584.900	424.341	0.900	α-Tocotrienoxyl radical	$C_{29}H_{43}O_2$		1.734	0.096	1.437
Serum	cold *vs.* hot; cold *vs.* normal; hot *vs.* normal; RA *vs.* normal	517.070	482.323	0.900	lysoPE 18:0	$C_{23}H_{48}NO_7P$		1.444	0.229	1.154
Serum	cold *vs.* hot; cold *vs.* normal; hot *vs.* normal; RA *vs.* normal	757.750	373.309	0.900	(+)-Dysideapalaunic acid	$C_{25}H_{40}O_2$		1.595	0.365	1.009
Serum	cold *vs.* hot; cold *vs.* normal; hot *vs.* normal; RA *vs.* normal	788.990	299.294	1.000	Nonadecanoic acid|Nonadecylic acid|FA	$C_{19}H_{38}O_2$	646-30-0	0.648	0.019	1.444
Serum	cold *vs.* normal	570.370	355.283	0.900	1-Linoleoyl-Rac-Glycerol	$C_{21}H_{38}O_4$	2277-28-3	0.693	0.283	1.151

Serum	hot *vs.* normal	576.970	562.422	0.890	CerP(d18:1/12:0)	$C_{30}H_{60}NO_6P$		0.704	0.141	1.112
Serum	cold *vs.* normal; hot *vs.* normal; RA *vs.* normal; cold *vs.* norm	589.220	552.402	0.890	PC(O-18:0/2:0)	$C_{28}H_{58}NO_7P$		1.279	0.264	1.018
Serum	cold *vs.* normal; hot *vs.* normal; RA *vs.* normal	890.180	786.599	0.890	1,2-Dioleoyl PC	$C_{44}H_{84}NO_8P$	56648-95-4	0.627	0.153	1.302
Serum	cold *vs.* normal	600.060	578.415	0.890	PC(22:1(11Z)/0:0)	$C_{30}H_{60}NO_7P$		1.952	0.211	1.234
Serum	cold *vs.* hot; cold *vs.* normal; hot *vs.* normal; RA *vs.* normal	568.670	302.244	1.000	Trihexyphenidyl|Trihexyphenidyl (INN)|	$C_{20}H_{31}NO$	144-11-6	0.314	0.013	2.066
Serum	cold *vs.* hot; cold *vs.* normal; hot *vs.* normal; RA *vs.* normal	622.890	562.416	0.890	lysoDGTS 23:5	$C_{33}H_{55}NO_6$		1.353	0.414	1.058
Serum	cold *vs.* hot	763.120	381.350	0.890	Brassicasterol|24-epibrassicasterol	$C_{28}H_{46}O$	474-67-9	1.958	0.099	1.533
Serum	hot *vs.* normal	489.000	482.359	0.880	1-O-Hexadecyl-lyso-sn-glycero-3-phosph	$C_{24}H_{52}NO_6P$		0.804	0.192	1.038
Serum	cold *vs.* hot; cold *vs.* normal	991.410	835.665	0.880	SM 42:2	$C_{47}H_{93}N_2O_6P$		0.390	0.334	1.258
Serum	cold *vs.* hot	599.100	304.260	1.000	Fenpropimorph	$C_{20}H_{33}NO$	67306-03-0	0.459	0.278	1.057
Serum	cold *vs.* normal; hot *vs.* normal; RA *vs.* normal	952.520	835.664	0.880	SM 42:2	$C_{47}H_{93}N_2O_6P$		2.965	0.060	1.662
Serum	cold *vs.* hot	1084.090	835.665	0.880	SM 42:2	$C_{47}H_{93}N_2O_6P$		1.554	0.241	1.140
Serum	cold *vs.* hot; hot *vs.* normal; cold *vs.* normal; RA *vs.* normal	523.040	535.394	0.880	6-(diaminomethylideneamino)-2-[[(4E,6E	$C_{29}H_{50}N_4O_5$		1.392	0.408	1.037
Serum	cold *vs.* hot; hot *vs.* normal; cold *vs.* normal; RA *vs.* normal	891.310	808.581	0.880	PC(18:0/20:5(5Z,8Z,11Z,14Z,17Z))[U]	$C_{46}H_{82}NO_8P$		0.158	0.039	1.727
Serum	cold *vs.* hot; hot *vs.* normal; cold *vs.* normal; RA *vs.* normal	948.760	849.653	0.880	MGDG 39:1|MGDG 15:0-24:1	$C_{48}H_{90}O_{10}$		2.414	0.187	1.164
Serum	cold *vs.* hot	967.260	809.650	0.880	SM 40:1	$C_{45}H_{91}N_2O_6P$		0.553	0.230	1.230
Serum	cold *vs.* normal	773.620	725.554	0.880	SM 34:1	$C_{39}H_{79}N_2O_6P$		0.679	0.268	1.135
Serum	cold *vs.* hot	916.710	820.619	0.870	PC(O-18:0/22:6(4Z,7Z,10Z,13Z,16Z,19Z	$C_{48}H_{86}NO_7P$		1.601	0.249	1.198
Serum	cold *vs.* hot	440.590	270.242	0.870	SPI_270.2429_14.9|N-deethylspiroxamin	$C_{16}H_{31}NO_2$		0.381	0.197	1.208
Serum	cold *vs.* normal; hot *vs.* normal; RA *vs.* normal	493.630	544.336	0.870	plasmenyl-PC 18:0	$C_{26}H_{52}NO_7P$		0.607	0.024	1.861
Serum	cold *vs.* normal	440.780	482.322	0.870	LysoPC(15:0)|lysoPC 15:0|1-pentadecano	$C_{23}H_{48}NO_7P$		1.491	0.071	1.578
Serum	cold *vs.* hot	1129.090	835.666	0.870	SM 42:2	$C_{47}H_{93}N_2O_6P$		3.114	0.014	1.992
Serum	cold *vs.* hot; cold *vs.* normal; hot *vs.* normal; RA *vs.* normal	553.100	523.264	0.870	(22E)-26,26,26,27,27,27-hexafluoro-1α;,	$C_{27}H_{36}F_6O_3$		0.072	0.003	2.220
Serum	cold *vs.* hot; cold *vs.* normal; hot *vs.* normal; RA *vs.* normal	859.250	766.573	0.870	PC(18:3(6Z,9Z,12Z)/P-18:1(11Z))|PC(18	$C_{44}H_{80}NO_7P$		0.649	0.056	1.644
Serum	cold *vs.* hot; cold *vs.* normal; hot *vs.* normal; RA *vs.* normal	898.900	819.606	0.870	PG(17:0/22:1(11Z))	$C_{45}H_{87}O_{10}P$		0.529	0.032	1.330
Serum	cold *vs.* hot	916.960	833.649	0.860	SM 42:3	$C_{47}H_{91}N_2O_6P$		1.904	0.090	1.694
Serum	cold *vs.* normal	695.600	251.200	0.860	7z,10z,13z-Hexadecatrienoic Acid	$C_{16}H_{26}O_2$		1.560	0.198	1.306
Serum	cold *vs.* normal	271.760	313.155	1.000	Phe-Phe	$C_{18}H_{20}N_2O_3$	2577-40-4	2.041	0.154	1.343
Serum	cold *vs.* hot	435.030	542.323	0.860	lysoPC 18:2	$C_{26}H_{50}NO_7P$		1.901	0.354	1.029
Serum	hot *vs.* normal; RA *vs.* normal; cold *vs.* hot	527.150	532.375	0.860	LysoPC(O-18:0)|lysoPC 18:0|PC(O-18:0/	$C_{26}H_{56}NO_6P$		0.624	0.111	1.157
Serum	hot *vs.* normal; RA *vs.* normal; cold *vs.* hot	655.650	275.273	0.860	Isodigeranyl	$C_{20}H_{34}$	5981-30-6	0.591	0.115	1.583
Serum	hot *vs.* normal; RA *vs.* normal; cold *vs.* hot	861.140	819.607	0.860	PG(17:1(9Z)/22:0)	$C_{45}H_{87}O_{10}P$		1.309	0.135	1.557
Serum	cold *vs.* normal	127.560	166.086	0.850	D-Phenylalanine	$C_9H_{11}NO_2$	673-06-3	1.445	0.001	2.370
Serum	cold *vs.* hot; hot *vs.* normal; RA *vs.* normal	655.730	293.283	0.850	9R,10S-Epoxy-3Z,6Z-eicosadiene	$C_{20}H_{36}O$		0.562	0.160	1.474

Serum	cold *vs.* hot; hot *vs.* normal; RA *vs.* normal	501.090	280.263	0.850	Linoleamide	$C_{18}H_{33}NO$	3072-13-7	0.397	0.086	1.559
Serum	cold *vs.* normal	593.880	313.273	1.000	2,4-dihydroxyheptadecyl acetate	$C_{19}H_{38}O_4$		0.509	0.269	1.218
Serum	cold *vs.* hot	984.610	836.669	0.850	SM d42:1|SM d16:0/26:1|SM(d16:0/26:1	$C_{47}H_{95}N_2O_6P$		0.455	0.146	1.609
Serum	cold *vs.* hot	801.090	802.535	0.850	PC 36:5	$C_{44}H_{78}NO_8P$		0.680	0.233	1.126
Serum	cold *vs.* normal; hot *vs.* normal; RA *vs.* normal; cold *vs.* hot	1328.500	800.669	0.850	SM(d17:1/24:1(15Z))	$C_{46}H_{92}N_2O_6P$		0.690	0.180	1.227
Serum	cold *vs.* normal; hot *vs.* normal; RA *vs.* normal; cold *vs.* hot	523.030	534.390	0.850	CerP 28:1	$C_{28}H_{56}NO_6P$		1.386	0.433	1.008
Serum	cold *vs.* normal	787.740	751.570	0.850	SM 36:2	$C_{41}H_{81}N_2O_6P$		0.395	0.061	1.604
Serum	cold *vs.* normal; hot *vs.* normal; RA *vs.* normal	729.740	723.540	0.840	SM 34:2	$C_{39}H_{77}N_2O_6P$		0.830	0.213	1.154
Serum	cold *vs.* hot	940.370	797.623	0.840	16:2-Glc-Campesterol	$C_{50}H_{84}O_7$		0.698	0.327	1.135
Serum	cold *vs.* hot; hot *vs.* normal; RA *vs.* normal	512.930	228.232	0.840	Halaminol A	$C_{14}H_{29}NO$		0.436	0.024	1.965
Serum	cold *vs.* hot; hot *vs.* normal; RA *vs.* normal	924.110	795.607	0.840	16:3-Glc-Campesterol	$C_{50}H_{82}O_7$		0.569	0.006	1.688
Serum	cold *vs.* hot; hot *vs.* normal; RA *vs.* normal	1329.030	788.616	0.830	PC(14:0/22:1(13Z))|PC(14:1(9Z)/22:0)|P	$C_{44}H_{86}NO_8P$		0.653	0.023	1.517
Serum	cold *vs.* hot	250.520	172.133	1.000	Gabapentin (JAN/USAN/INN)|Neurontin	$C_9H_{17}NO_2$	60142-96-3	0.261	0.277	1.062
Serum	cold *vs.* hot; cold *vs.* normal; hot *vs.* normal; RA *vs.* normal	621.630	679.472	0.830	PA(P-16:0/20:5(5Z,8Z,11Z,14Z,17Z))	$C_{39}H_{67}O_7P$		1.709	0.035	1.905
Serum	cold *vs.* hot; cold *vs.* normal; hot *vs.* normal; RA *vs.* normal	572.770	706.463	0.830	PG 30:3|PG 13:1-17:2|PG(13:1/17:2)	$C_{36}H_{65}O_{10}P$		1.579	0.056	1.697
Serum	cold *vs.* hot; cold *vs.* normal; hot *vs.* normal; RA *vs.* normal	890.430	789.608	0.830	PG(O-16:0/22:2(13Z,16Z))	$C_{44}H_{85}O_9P$		0.484	0.032	1.773
Serum	cold *vs.* hot; cold *vs.* normal; hot *vs.* normal; RA *vs.* normal	717.080	697.523	0.830	SM 32:1	$C_{37}H_{75}N_2O_6P$		0.742	0.089	1.583
Serum	cold *vs.* normal	502.380	532.335	0.830	lysoPC 17:0	$C_{25}H_{52}NO_7P$		1.226	0.131	1.342
Serum	RA *vs.* normal	432.240	318.239	1.000	Trihexyphenidyl N-oxide	$C_{20}H_{31}NO_2$	161564-79-	1.793	0.099	1.184
Serum	cold *vs.* hot; RA *vs.* normal	534.630	188.127	0.820	(E)-2-Butenyl-4-methyl-threonine|2-Bute	$C_9H_{17}NO_3$	81135-57-1	0.419	0.169	1.288
Serum	cold *vs.* hot	95.840	216.922	0.810	Methyl 2-propenyl pentasulfide	$C_4H_8S_5$	118023-99-	1.392	0.287	1.018
Serum	hot *vs.* normal; RA *vs.* normal	618.050	564.437	0.810	CerP 30:0	$C_{30}H_{62}NO_6P$		0.695	0.085	1.217
Serum	cold *vs.* hot	913.190	812.615	0.810	PC 36:0|PC(12:0/24:0)	$C_{44}H_{88}NO_8P$		1.699	0.454	1.122
Serum	cold *vs.* hot	524.200	214.179	0.810	N-(9-Oxodecyl)acetamide	$C_{12}H_{23}NO_2$		0.376	0.186	1.248
Serum	hot *vs.* normal; RA *vs.* normal	473.780	518.320	0.810	lysoPC 16:0	$C_{24}H_{50}NO_7P$		0.712	0.114	1.016
Serum	hot *vs.* normal	501.330	320.255	1.000	N-hydroxy arachidonoyl amine	$C_{20}H_{33}NO_2$		1.434	0.101	1.058
Serum	cold *vs.* hot; cold *vs.* normal; hot *vs.* normal; RA *vs.* normal	577.990	694.463	0.800	BMP 29:2|BMP 13:1-16:1|BMP(13:1/16:	$C_{35}H_{65}O_{10}P$		1.186	0.492	1.173
Serum	cold *vs.* normal	530.440	546.351	0.800	lysoPC 18:0	$C_{26}H_{54}NO_7P$		1.156	0.303	1.011
Serum	RA *vs.* normal	544.050	399.324	0.800	Calcitriol|1α,25-Dihydroxyvitamin D3|(1	$C_{27}H_{44}O_3$	32222-06-3	0.774	0.147	1.083
Serum	cold *vs.* hot; hot *vs.* normal; RA *vs.* normal	1153.660	898.784	0.800	TG 54:5	$C_{57}H_{100}O_6$		0.260	0.145	1.383
Serum	RA *vs.* normal	679.430	591.457	0.800	DG 34:6	$C_{37}H_{60}O_5$		0.701	0.075	1.234
Serum	cold *vs.* hot	788.760	603.532	0.790	1-(14-methyl-pentadecanoyl)-2-(8-[3]-lad	$C_{39}H_{70}O_4$		0.749	0.304	1.116
Serum	cold *vs.* normal	530.420	524.370	0.790	1-Stearoyl-Sn-Glycerol-3-Phosphocholin	$C_{26}H_{54}NO_7P$	19420-57-6	1.116	0.067	1.560
Serum	cold *vs.* normal; hot *vs.* normal	876.490	816.587	0.790	PC(P-18:1(11Z)/22:6(4Z,7Z,10Z,13Z,16	$C_{48}H_{82}NO_7P$		0.492	0.233	1.211

Serum	cold *vs.* normal	603.780	329.246	1.000	Docosahexaenoic acid	$C_{22}H_{32}O_2$	6217-54-5	1.758	0.249	1.058
Serum	cold *vs.* hot; cold *vs.* normal; hot *vs.* normal; RA *vs.* normal	676.710	673.526	0.790	SM d32:2|SM d14:1/18:1|SM(d14:1/18:1	$C_{37}H_{73}N_2O_6P$		1.395	0.303	1.038
Serum	cold *vs.* hot; cold *vs.* normal; hot *vs.* normal; RA *vs.* normal	928.420	748.610	0.790	DGTS 35:3|DGTS 14:1-21:2|DGTS(14:1	$C_{45}H_{81}NO_7$		0.454	0.021	1.882
Serum	cold *vs.* hot; cold *vs.* normal; hot *vs.* normal; RA *vs.* normal	723.470	428.372	0.780	Sorbitane Monooleate - Polysorbate 80 in-source fragment			2.082	0.051	1.257
Serum	cold *vs.* hot; cold *vs.* normal; hot *vs.* normal; RA *vs.* normal	612.430	331.224	1.000	Deoxycorticosterone	$C_{21}H_{30}O_3$	64-85-7	0.568	0.430	1.074
Serum	cold *vs.* normal	860.100	792.587	0.780	PC(P-16:0/22:5(4Z,7Z,10Z,13Z,16Z))	$C_{46}H_{82}NO_7P$		0.488	0.173	1.277
Serum	cold *vs.* normal; RA *vs.* normal	827.840	766.536	0.780	PE 38:5	$C_{43}H_{76}NO_8P$		0.589	0.234	1.095
Serum	cold *vs.* normal	305.310	169.049	0.780	3,4-Dihydroxyphenylacetate|3,4-Dihydro:	$C_8H_8O_4$	102-32-9	2.431	0.186	1.247
Serum	hot *vs.* normal	281.640	381.198	0.780	Beraprost	$C_{24}H_{30}O_5$	88475-69-8	0.531	0.168	1.022
Serum	cold *vs.* normal	578.590	695.466	0.780	methyl 13-sophorosyloxydocosanoate	$C_{35}H_{66}O_{13}$		1.864	0.022	1.884
Serum	cold *vs.* hot; hot *vs.* normal; RA *vs.* normal; cold *vs.* normal	609.930	536.405	0.780	dl-α-Tocopherol nicotinate|Tocopheryl ni	$C_{35}H_{53}NO_3$	51898-34-1	1.795	0.301	1.131
Serum	cold *vs.* hot; hot *vs.* normal; RA *vs.* normal; cold *vs.* normal	383.780	359.287	0.780	15(R)-Pinane Thromboxane A2	$C_{24}H_{40}O_3$		1.222	0.087	1.096
Serum	cold *vs.* hot; hot *vs.* normal; RA *vs.* normal; cold *vs.* normal	744.190	413.264	0.780	Bis(2-ethylhexyl)-phthalate	$C_{24}H_{38}O_4$	117-81-7	0.729	0.473	1.020
Serum	cold *vs.* normal	464.160	544.338	0.780	lysoPC 20:4	$C_{28}H_{50}NO_7P$		1.224	0.149	1.285
Serum	cold *vs.* hot; cold *vs.* normal	703.410	687.541	0.780	SM d33:2|SM d17:1/16:1|SM(d17:1/16:1	$C_{38}H_{75}N_2O_6P$		1.447	0.293	1.103
Serum	cold *vs.* hot; cold *vs.* normal	622.250	678.469	0.770	PS(P-16:0/13:0)	$C_{35}H_{68}NO_9P$		1.753	0.025	1.974
Serum	cold *vs.* hot; cold *vs.* normal	414.530	643.283	0.770	(2alpha,5alpha,9alpha,10beta,14beta)-2,5	$C_{33}H_{48}O_{10}$		2.050	0.156	1.365
Serum	cold *vs.* hot	696.830	477.392	0.770	Sarcoaldesterol A|(3β,5α,6β,11α)-Gorgos	$C_{30}H_{52}O_4$		1.378	0.321	1.087
Serum	hot *vs.* normal; RA *vs.* normal	635.200	590.453	0.770	CerP 32:1	$C_{32}H_{64}NO_6P$		0.640	0.083	1.213
Serum	hot *vs.* normal; RA *vs.* normal	854.570	816.588	0.770	PC(P-18:1(9Z)/22:6(4Z,7Z,10Z,13Z,16Z	$C_{48}H_{82}NO_7P$		0.645	0.127	1.022
Serum	cold *vs.* normal	836.710	762.579	0.770	GlcADG 32:0|GlcADG 15:0-17:0|GlcAD	$C_{41}H_{76}O_{11}$		0.650	0.356	1.021
Serum	cold *vs.* normal; hot *vs.* normal; RA *vs.* normal; cold *vs.* hot	872.920	745.588	0.770	PE-Cer(d15:2(4E,6E)/24:0(2OH))	$C_{41}H_{81}N_2O_7P$		0.521	0.204	1.171
Serum	cold *vs.* normal; hot *vs.* normal; RA *vs.* normal; cold *vs.* hot	657.200	686.356	0.760	Avadharidine|Aconitane-7,8-diol, 4-(((2-(	$C_{36}H_{51}N_3O_{10}$	509-16-0	2.149	0.036	1.805
Serum	hot *vs.* normal	91.830	338.050	0.760	Famotidine	$C_8H_{15}N_7O_2S_3$	76824-35-6	1.174	0.628	1.106
Serum	cold *vs.* hot; hot *vs.* normal; cold *vs.* normal; RA *vs.* normal	372.760	314.231	0.760	9-Decenoylcarnitine	$C_{17}H_{31}NO_4$		2.294	0.071	1.737
Serum	cold *vs.* hot; hot *vs.* normal; cold *vs.* normal; RA *vs.* normal	635.380	816.701	0.760	HexCer-ADS d41:0|HexCer-ADS d27:0/	$C_{47}H_{93}NO_9$		0.554	0.201	1.136
Serum	cold *vs.* hot; hot *vs.* normal; cold *vs.* normal; RA *vs.* normal	782.550	706.584	0.760	PC(O-16:0/15:0)	$C_{39}H_{80}NO_7P$		1.399	0.292	1.041
Serum	cold *vs.* hot; hot *vs.* normal; cold *vs.* normal; RA *vs.* normal	393.130	387.179	0.760	Burseran|(+)-Burseran	$C_{22}H_{26}O_6$	23284-23-3	1.318	0.078	1.154
Serum	cold *vs.* hot	296.840	186.148	1.000	N-methyl-Gabapentin	$C_{10}H_{19}NO_2$	102937-74-	0.286	0.236	1.126
Serum	hot *vs.* normal	896.030	719.576	0.750	PE-Cer(d15:1(4E)/22:0(2OH))	$C_{39}H_{79}N_2O_7P$		0.419	0.035	1.316
Serum	hot *vs.* normal	472.780	506.360	0.750	LysoPC(P-18:1(9Z))|PC(P-18:1(9Z)/0:0)	$C_{26}H_{52}NO_6P$		0.756	0.195	1.049
Serum	RA *vs.* normal	696.330	816.702	0.750	HexCer-ADS d41:0|HexCer-ADS d21:0/:	$C_{47}H_{93}NO_9$		0.525	0.091	1.017
Serum	hot *vs.* normal; RA *vs.* normal; cold *vs.* hot	819.850	844.524	0.740	Scytophycin D	$C_{45}H_{75}NO_{12}$		0.661	0.054	1.213
Serum	hot *vs.* normal; RA *vs.* normal; cold *vs.* hot	855.700	746.595	0.740	DGTS 35:4|DGTS 19:1-16:3	$C_{45}H_{79}NO_7$		1.729	0.175	1.654

Serum	cold *vs.* hot	567.960	264.240	0.740	1,7-Dimethyl-7-(4-methyl-3-penten-1-yl)	$C_{15}H_{26}O$		0.334	0.196	1.463
Serum	cold *vs.* normal; hot *vs.* normal; RA *vs.* normal	894.240	825.559	0.740	MGDG 38:6	$C_{47}H_{78}O_{10}$		0.112	0.066	1.589
Serum	hot *vs.* normal	153.410	349.183	1.000	Enalaprilat|Enalaprilate	$C_{18}H_{24}N_2O_5$	76420-72-9	0.475	0.160	1.158
Serum	cold *vs.* normal; hot *vs.* normal; RA *vs.* normal; cold *vs.* hot	855.080	745.619	0.740	SM d37:1|SM d18:1/19:0|SM(d18:1/19:0	$C_{42}H_{85}N_2O_6P$		0.726	0.337	1.022
Serum	cold *vs.* normal; hot *vs.* normal; RA *vs.* normal; cold *vs.* hot	379.960	583.254	0.740	Biliverdin	$C_{33}H_{34}N_4O_6$	114-25-0	0.424	0.046	1.222
Serum	cold *vs.* normal; hot *vs.* normal; RA *vs.* normal; cold *vs.* hot	566.520	652.443	0.740	PE 31:6e|PE 22:6e/9:0|EtherPE 31:6e|PE	$C_{36}H_{62}NO_7P$		1.412	0.133	1.406
Serum	cold *vs.* normal; hot *vs.* normal; RA *vs.* normal; cold *vs.* hot	810.810	820.524	0.740	SQDG 33:3|SQDG 14:1-19:2|SQDG(14:	$C_{42}H_{74}O_{12}S$		0.588	0.079	1.571
Serum	cold *vs.* normal; hot *vs.* normal; RA *vs.* normal; cold *vs.* hot	866.340	790.570	0.740	plasmenyl-PC 36:3	$C_{44}H_{82}NO_7P$		0.437	0.130	1.400
Serum	cold *vs.* normal; hot *vs.* normal; RA *vs.* normal; cold *vs.* hot	1072.260	814.687	0.730	TAG 48:5|TAG 13:0-15:0-20:5|TG 48:5|	$C_{51}H_{88}O_6$		1.485	0.319	1.083
Serum	cold *vs.* normal; hot *vs.* normal; RA *vs.* normal; cold *vs.* hot	277.610	352.164	1.000	O-β-D-Xylosylzeatin|O-β-D-Xyloxylzeati	$C_{15}H_{21}N_5O_5$		0.525	0.250	1.359
Serum	cold *vs.* hot	687.060	343.263	0.730	4,7,10,13,16,19-Docosahexaenoic acid m	$C_{23}H_{34}O_2$	2566-90-7	1.397	0.431	1.067
Serum	hot *vs.* normal	893.650	824.555	0.730	C18-OH Sulfatide	$C_{42}H_{81}NO_{12}S$		0.288	0.004	1.663
Serum	cold *vs.* normal	835.360	796.524	0.730	C16-OH Sulfatide	$C_{40}H_{77}NO_{12}S$		0.180	0.062	1.586
Serum	cold *vs.* hot	577.380	489.354	0.730	Euscaphic acid	$C_{30}H_{48}O_5$	53155-25-2	1.537	0.173	1.302
Serum	cold *vs.* hot; hot *vs.* normal; RA *vs.* normal	943.770	773.648	0.730	PA(O-20:0/22:1(11Z))	$C_{45}H_{89}O_7P$		0.251	0.031	1.939
Serum	cold *vs.* hot	1149.000	872.768	0.730	TG 52:4	$C_{55}H_{98}O_6$		0.393	0.265	1.183
Serum	cold *vs.* hot	807.580	383.366	0.730	Campesterol|CAMPESTEROL|(24R)-ER	$C_{28}H_{48}O$	474-62-4	1.348	0.158	1.402
Serum	cold *vs.* hot; cold *vs.* normal; hot *vs.* normal; RA *vs.* normal	725.810	816.701	0.720	HexCer-NP t41:0|HexCer-NP t18:0/23:0	$C_{47}H_{93}NO_9$		0.341	0.181	1.259
Serum	cold *vs.* hot	453.720	265.142	0.720	Coronopilin	$C_{15}H_{20}O_4$	2571-81-5	1.959	0.125	1.397
Serum	cold *vs.* normal; hot *vs.* normal; RA *vs.* normal; cold *vs.* hot	127.570	167.089	0.720	2-methyl-3-Pyrimidin-2-yl-Propionic Aci	$C_8H_{10}N_2O_2$		1.578	0.010	2.047
Serum	cold *vs.* normal; hot *vs.* normal; RA *vs.* normal; cold *vs.* hot	984.270	814.686	0.720	HexCer-NP t41:1|HexCer-NP t21:1/20:0	$C_{47}H_{91}NO_9$		0.378	0.030	1.357
Serum	cold *vs.* normal; hot *vs.* normal; RA *vs.* normal; cold *vs.* hot	846.740	783.636	0.720	SM 38:0|SM(d16:0/22:0)	$C_{43}H_{89}N_2O_6P$		1.308	0.217	1.236
Serum	cold *vs.* normal	478.000	592.335	0.720	lysoPC 22:5	$C_{30}H_{52}NO_7P$		0.666	0.159	1.257
Serum	cold *vs.* hot	652.210	491.369	0.720	Ganoderiol H	$C_{30}H_{50}O_5$	114612-72-	2.029	0.049	1.727
Serum	cold *vs.* normal	452.890	368.278	1.000	3, 5-Tetradecadiencarnitine	$C_{21}H_{37}NO_4$		1.763	0.262	1.007
Serum	cold *vs.* normal	618.420	421.330	0.710	6-Ethylchenodeoxycholic acid|6-ECDCA	$C_{26}H_{44}O_4$	459789-99-	2.201	0.066	1.709
Serum	cold *vs.* hot; cold *vs.* normal; hot *vs.* normal; RA *vs.* normal	635.590	244.190	0.710	2-(7-hydroxy-6-methyloctyl)-2H-furan-5-	$C_{13}H_{22}O_3$		1.577	0.006	2.214
Serum	cold *vs.* hot; cold *vs.* normal; hot *vs.* normal; RA *vs.* normal	706.340	295.263	0.710	9,12-Octadecadienoic acid (Z,Z)-, methyl	$C_{19}H_{34}O_2$	112-63-0	0.771	0.094	1.088
Serum	cold *vs.* normal	1328.020	801.683	0.710	SM(d17:1/24:0)	$C_{46}H_{93}N_2O_6P$		1.168	0.302	1.047
Serum	cold *vs.* hot	914.930	744.588	0.710	1-Hexadecanoyl-2-(9Z-octadecenoyl)-sn-	$C_{42}H_{83}NO_7P$		1.480	0.162	1.296
Serum	cold *vs.* normal	1020.540	369.351	1.000	Lathosterol|5α-Cholest-7-en-3β-ol	$C_{27}H_{46}O$	80-99-9	3.493	0.159	1.292
Serum	hot *vs.* normal	723.770	284.295	0.710	Stearamide			1.220	0.089	1.112
Serum	cold *vs.* hot; hot *vs.* normal; RA *vs.* normal; cold *vs.* normal	941.720	774.653	0.700	HexCer-ADS d38:0|HexCer-ADS d25:0/	$C_{44}H_{87}NO_9$		0.485	0.154	1.559
Serum	cold *vs.* hot; hot *vs.* normal; RA *vs.* normal; cold *vs.* normal	566.520	651.440	0.700	Progesterone			1.385	0.167	1.294
Serum	cold *vs.* hot; hot *vs.* normal; RA *vs.* normal; cold *vs.* normal	1000.540	650.643	0.700	N-Lignoceroylsphingosine	$C_{42}H_{83}NO_3$	34435-05-7	0.510	0.123	1.544

Serum	cold *vs.* hot	582.120	419.314	0.700	(24R)-1α;,24-dihydroxy-22-oxavitamin D	$C_{26}H_{42}O_4$		1.526	0.300	1.031
Serum	hot *vs.* normal; RA *vs.* normal	1107.190	870.753	0.700	TG 52:5	$C_{55}H_{96}O_6$		2.027	0.059	1.244
Serum	cold *vs.* normal	127.570	120.080	0.700	Indoline	C_8H_9N	496-15-1	1.382	0.031	1.778
Serum	cold *vs.* hot	612.660	281.247	0.690	β-Linoleic acid	$C_{18}H_{32}O_2$	60-33-3	2.312	0.013	2.007
Serum	cold *vs.* hot	686.280	463.377	0.690	2α;-(3-Hydroxypropyl)-1α;,25-dihydroxy	$C_{29}H_{50}O_4$		1.487	0.295	1.153
Serum	cold *vs.* normal	757.610	773.615	0.690	PA(21:0/20:1(11Z))	$C_{44}H_{85}O_8P$		1.747	0.192	1.157
Serum	cold *vs.* normal; hot *vs.* normal; RA *vs.* normal	891.600	759.635	0.690	SM(d18:1/20:0)	$C_{43}H_{87}N_2O_6P$		0.384	0.023	1.878
Serum	cold *vs.* hot	760.260	323.293	0.690	Heneicosapentaenoic Acid-d6	$C_{21}H_{26}D_6O_2$		1.337	0.419	1.014
Serum	cold *vs.* hot; hot *vs.* normal; RA *vs.* normal; cold *vs.* normal	903.400	785.652	0.680	SM(d16:1/24:1(15Z))	$C_{45}H_{89}N_2O_6P$		0.522	0.028	1.950
Serum	cold *vs.* hot; hot *vs.* normal; RA *vs.* normal; cold *vs.* normal	708.120	771.599	0.680	PA(19:0/22:2(13Z,16Z))	$C_{44}H_{83}O_8P$		2.273	0.001	2.346
Serum	cold *vs.* hot	571.850	374.301	1.000	Arachidonoylmorpholine	$C_{24}H_{39}NO_2$		1.554	0.272	1.431
Serum	cold *vs.* hot; RA *vs.* normal; hot *vs.* normal; cold *vs.* normal	576.040	256.264	0.670	Altretamine			0.605	0.009	2.073
Serum	cold *vs.* hot; RA *vs.* normal; hot *vs.* normal; cold *vs.* normal	490.790	318.299	0.670	Phytosphingosine|4-D-Hydroxysphingani	$C_{18}H_{39}NO_3$	554-62-1	0.344	0.263	1.094
Serum	cold *vs.* hot; RA *vs.* normal; hot *vs.* normal; cold *vs.* normal	569.110	814.687	0.670	HexCer-NP t41:1|HexCer-NP t24:1/17:0	$C_{47}H_{91}NO_9$		0.461	0.232	1.227
Serum	cold *vs.* hot; RA *vs.* normal; hot *vs.* normal; cold *vs.* normal	452.220	200.200	0.660	Dodecanamide|dodecanamide	$C_{12}H_{25}NO$		0.390	0.021	2.015
Serum	cold *vs.* hot; RA *vs.* normal; hot *vs.* normal; cold *vs.* normal	656.630	341.304	0.660	Glycidyl stearate	$C_{21}H_{40}O_3$	7460-84-6	0.291	0.311	1.122
Serum	cold *vs.* hot; RA *vs.* normal; hot *vs.* normal; cold *vs.* normal	830.080	397.382	0.660	β-Sitosterol|Sitosterol|Harzol (TN)|γ-Sito:	$C_{29}H_{50}O$	83-46-5|83-	1.545	0.394	1.084
Serum	cold *vs.* hot; RA *vs.* normal; hot *vs.* normal; cold *vs.* normal	701.040	745.583	0.660	PA(21:0/18:1(9Z))	$C_{42}H_{81}O_8P$		2.097	0.012	2.026
Serum	cold *vs.* hot	941.310	780.587	0.660	PE(22:4(7Z,10Z,13Z,16Z)/P-18:0)	$C_{45}H_{82}NO_7P$		0.151	0.218	1.186
Serum	cold *vs.* hot; hot *vs.* normal; RA *vs.* normal; cold *vs.* normal	846.950	861.667	0.660	PG(20:1(11Z)/22:0)	$C_{48}H_{93}O_{10}P$		1.489	0.192	1.245
Serum	cold *vs.* hot; hot *vs.* normal; RA *vs.* normal; cold *vs.* normal	607.870	305.247	0.660	Mesterolone	$C_{20}H_{32}O_2$	1424-00-6	1.872	0.096	1.499
Serum	cold *vs.* hot	556.730	376.317	1.000	N-(1,1-dimethyl-2-hydroxy-ethyl) arachic	$C_{24}H_{41}NO_2$		1.264	0.373	1.099
Serum	cold *vs.* hot; cold *vs.* normal	603.700	585.269	0.650	Bilirubin|4E,15Z-Bilirubin IXa	$C_{33}H_{36}N_4O_6$	635-65-4|69	0.706	0.240	1.554
Serum	cold *vs.* normal	789.680	815.698	0.650	SM(d18:0/24:1(15Z))	$C_{47}H_{95}N_2O_6P$		0.527	0.282	1.045
Serum	cold *vs.* hot; RA *vs.* normal	142.200	193.101	0.650	Cotinine N-oxide	$C_{10}H_{12}N_2O_2$	36508-80-2	0.676	0.351	1.079
Serum	cold *vs.* hot	574.200	378.268	1.000	Karakoline|Aconitane-1,8,14-triol, 20-eth	$C_{22}H_{35}NO_4$	39089-30-0	0.682	0.264	1.177
Serum	cold *vs.* hot	858.250	809.651	0.650	SM 40:1	$C_{45}H_{91}N_2O_6P$		1.547	0.040	1.853
Serum	hot *vs.* normal	343.240	226.180	0.650	(8S,Z)-6-((S)-3-hydroxy-2-methylpropyli	$C_{13}H_{23}NO_2$		1.699	0.003	1.713
Serum	cold *vs.* normal; RA *vs.* normal; cold *vs.* hot; hot *vs.* normal	786.200	767.544	0.640	Finasteride			0.643	0.259	1.029
Serum	cold *vs.* normal; RA *vs.* normal; cold *vs.* hot; hot *vs.* normal	700.970	746.587	0.640	MGDG 32:1	$C_{41}H_{76}O_{10}$		2.492	0.007	2.109
Serum	cold *vs.* normal; RA *vs.* normal; cold *vs.* hot; hot *vs.* normal	657.460	359.314	0.640	Hexadecyl Acetyl Glycerol	$C_{21}H_{42}O_4$	77133-35-8	0.257	0.318	1.118
Serum	cold *vs.* normal; RA *vs.* normal; cold *vs.* hot; hot *vs.* normal	840.230	757.619	0.640	SM d38:2|SM d15:2/23:0|SM(d15:2/23:0	$C_{43}H_{85}N_2O_6P$		0.512	0.056	1.620
Serum	cold *vs.* hot	585.980	405.299	0.640	MG(22:5(7Z,10Z,13Z,16Z,19Z)/0:0/0:0)	$C_{25}H_{40}O_4$		0.586	0.337	1.260
Serum	cold *vs.* hot	351.790	466.315	0.640	Glycocholic acid	$C_{26}H_{43}NO_6$	475-31-0	0.243	0.299	1.064
Serum	cold *vs.* normal	271.570	315.159	0.640	Clomipramine|Clomipramine (INN)|Anaf	$C_{19}H_{23}ClN_2$	303-49-1	1.558	0.258	1.072

Serum	cold *vs.* hot; hot *vs.* normal; RA *vs.* normal; cold *vs.* normal	358.040	586.273	0.640	PS 20:2|PS 3:0-17:2|PS(3:0/17:2)	$C_{26}H_{46}NO_{10}P$		1.980	0.281	1.042
Serum	cold *vs.* hot; hot *vs.* normal; RA *vs.* normal; cold *vs.* normal	826.180	754.587	0.640	HexCer-AS d37:3|HexCer-AS d21:1/16:2	$C_{43}H_{79}NO_9$		1.670	0.218	1.610
Serum	cold *vs.* hot	167.010	393.209	1.000	Dexamethasone	$C_{22}H_{29}FO_5$	1950/2/2	3.947	0.183	1.266
Serum	cold *vs.* normal; hot *vs.* normal; RA *vs.* normal	610.970	814.686	0.640	HexCer-AS d41:1|HexCer-AS d21:1/20:(	$C_{47}H_{91}NO_9$		0.426	0.131	1.315
Serum	cold *vs.* normal	626.150	445.330	0.630	(3β,5α,6β,9α,22E,24R)-5,9-Epidioxyergo	$C_{28}H_{44}O_4$	348611-57-	1.417	0.263	1.163
Serum	cold *vs.* hot; cold *vs.* normal	1328.190	813.683	0.630	SM(d18:1/24:1(15Z))	$C_{47}H_{93}N_2O_6P$		1.212	0.155	1.402
Serum	RA *vs.* normal	713.640	717.552	0.630	PA(15:0/22:1(11Z))	$C_{40}H_{77}O_8P$		0.835	0.155	1.019
Serum	cold *vs.* normal; hot *vs.* normal; RA *vs.* normal; cold *vs.* hot	943.990	646.612	0.630	Cer-NS d42:3; Cer-NS d19:3/23:0	$C_{42}H_{79}NO_3$		0.584	0.020	1.898
Serum	cold *vs.* normal; hot *vs.* normal; RA *vs.* normal; cold *vs.* hot	614.500	401.344	1.000	7-Ketocholesterol	$C_{27}H_{44}O_2$	566-28-9	2.255	0.053	1.711
Serum	cold *vs.* normal	832.870	805.554	0.630	NBD-Stearoyl-2-Arachidonoyl-sn-glycer	$C_{47}H_{74}N_4O_8$		0.712	0.517	1.058
Serum	cold *vs.* hot; cold *vs.* normal; hot *vs.* normal; RA *vs.* normal	753.040	719.568	0.630	PA(15:0/22:0)	$C_{40}H_{79}O_8P$		0.842	0.580	1.066
Serum	cold *vs.* hot; cold *vs.* normal; hot *vs.* normal; RA *vs.* normal	1116.860	896.768	0.630	TG 54:6	$C_{57}H_{98}O_6$		2.612	0.032	1.351
Serum	cold *vs.* hot; cold *vs.* normal; hot *vs.* normal; RA *vs.* normal	796.870	717.589	0.630	PA(O-18:0/20:1(11Z))	$C_{41}H_{81}O_7P$		0.722	0.040	1.814
Serum	cold *vs.* hot	427.840	291.271	0.620	Aphidicolan-16β-ol|aphidicolan-16β-ol	$C_{20}H_{34}O$	101143-85-	0.489	0.298	1.012
Serum	cold *vs.* hot; RA *vs.* normal; cold *vs.* hot; cold *vs.* normal	758.750	463.377	0.620	(3β,22R,23R,24S)-3,22,23-Trihydroxysti	$C_{29}H_{50}O_4$	90524-90-6	1.560	0.300	1.169
Serum	cold *vs.* hot; RA *vs.* normal; cold *vs.* hot; cold *vs.* normal	753.110	720.571	0.610	MGDG 30:0	$C_{39}H_{74}O_{10}$		0.854	0.614	1.054
Serum	cold *vs.* hot	1130.860	922.783	0.610	TG 56:7	$C_{59}H_{100}O_6$		0.385	0.300	1.110
Serum	hot *vs.* normal; RA *vs.* normal	786.680	729.589	0.610	N-Oleoyl-D-sphingomyelin	$C_{41}H_{81}N_2O_6P$	108392-10-	0.764	0.068	1.146
Serum	cold *vs.* hot	567.790	263.236	0.610	2-(5,8-Tetradecadienyl)cyclobutanone	$C_{18}H_{30}O$	152245-81-	0.376	0.156	1.392
Serum	cold *vs.* hot; hot *vs.* normal; RA *vs.* normal; cold *vs.* normal	496.310	298.273	0.610	3-ketosphingosine	$C_{18}H_{35}NO_2$		0.393	0.107	1.484
Serum	cold *vs.* hot; hot *vs.* normal; RA *vs.* normal; cold *vs.* normal	974.570	789.675	0.610	SM(d18:0/22:0)	$C_{45}H_{93}N_2O_6P$		0.396	0.278	1.166
Serum	cold *vs.* hot; hot *vs.* normal; RA *vs.* normal; cold *vs.* normal	629.840	447.345	0.600	3-Dehydroteasterone	$C_{28}H_{46}O_4$		1.595	0.218	1.160
Serum	cold *vs.* hot; hot *vs.* normal; RA *vs.* normal; cold *vs.* normal	548.430	636.421	0.600	SM d28:3|SM d16:3/12:0|SM(d16:3/12:0	$C_{33}H_{63}N_2O_6P$		1.683	0.100	1.597
Serum	cold *vs.* hot; hot *vs.* normal; RA *vs.* normal; cold *vs.* normal	888.330	760.638	0.600	HexCer-ADS d37:0|HexCer-ADS d19:0/	$C_{43}H_{85}NO_9$		0.337	0.076	1.516
Serum	cold *vs.* hot	907.970	811.667	0.600	SM 40:0	$C_{45}H_{93}N_2O_6P$		1.618	0.280	1.193
Serum	hot *vs.* normal; RA *vs.* normal; cold *vs.* hot; cold *vs.* normal	1219.750	848.769	0.600	TG 50:2	$C_{53}H_{98}O_6$		1.889	0.128	1.240
Serum	hot *vs.* normal; RA *vs.* normal; cold *vs.* hot; cold *vs.* normal	1328.310	787.667	0.590	SM d40:1|SM d24:1/16:0|SM(d24:1/16:0	$C_{45}H_{91}N_2O_6P$		0.730	0.234	1.304
Serum	cold *vs.* hot	324.550	200.164	1.000	11-nitro-1-undecene	$C_{11}H_{21}NO_2$		0.311	0.250	1.094
Serum	hot *vs.* normal; RA *vs.* normal; cold *vs.* normal; RA *vs.* norma	797.540	719.596	0.590	PA(O-18:0/20:0)	$C_{41}H_{83}O_7P$		0.683	0.006	1.603
Serum	hot *vs.* normal; RA *vs.* normal; cold *vs.* normal; RA *vs.* norma	486.430	440.312	0.590	PE(O-16:0/0:0)	$C_{21}H_{46}NO_6P$		1.491	0.150	1.262
Serum	cold *vs.* hot	524.020	230.174	0.590	2-(6-hydroxy-6-methylheptyl)-2H-furan-5	$C_{12}H_{20}O_3$		0.387	0.163	1.296
Serum	hot *vs.* normal	435.630	500.275	0.590	lysoPE 18:2	$C_{23}H_{44}NO_7P$		1.611	0.141	1.001
Serum	RA *vs.* normal	692.410	449.361	0.580	Typhasterol|2-Deoxycastasterone	$C_{28}H_{48}O_4$	87734-68-7	1.516	0.122	1.034
Serum	cold *vs.* hot	704.960	862.624	0.580	Lactosylceramide (d18:1/16:0)|C16 Lacto	$C_{46}H_{87}NO_{13}$		0.906	0.664	1.001

Serum	cold *vs.* normal	707.220	405.371	0.580	5α-Cholestan-5α,6β-diol	$C_{27}H_{48}O_2$		1.813	0.157	1.275
Serum	cold *vs.* hot	183.000	438.238	1.000	Lunarine	$C_{25}H_{31}N_3O_4$	24185-51-1	3.346	0.223	1.198
Serum	cold *vs.* hot; hot *vs.* normal; RA *vs.* normal	357.870	585.270	0.580	4Z,15E-Bilirubin IXa	$C_{33}H_{36}N_4O_6$		1.984	0.285	1.036
Serum	hot *vs.* normal	983.500	813.684	0.570	SM d42:2|SM d20:0/22:2|SM(d20:0/22:2	$C_{47}H_{93}N_2O_6P$		0.272	0.002	1.795
Serum	cold *vs.* hot; hot *vs.* normal; RA *vs.* normal; cold *vs.* normal	525.410	352.245	0.570	Dutasteride			0.459	0.173	1.330
Serum	cold *vs.* hot; hot *vs.* normal; RA *vs.* normal; cold *vs.* normal	472.850	548.358	0.570	DGTS 21:5|DGTS 3:0-18:5|DGTS(3:0/1	$C_{31}H_{49}NO_7$		1.372	0.303	1.066
Serum	cold *vs.* hot; hot *vs.* normal; RA *vs.* normal; cold *vs.* normal	942.720	799.667	0.570	Levothyroxine sodium anhydrous|Levoth	$C_{15}H_{10}I_4NO_4$. Na	1955/3/8	3.257	0.128	1.326
Serum	cold *vs.* hot; hot *vs.* normal; RA *vs.* normal; cold *vs.* normal	695.730	815.698	0.560	SM d42:1|SM d27:0/15:1|SM(d27:0/15:1	$C_{47}H_{95}N_2O_6P$		0.607	0.118	1.026
Serum	hot *vs.* normal	183.880	453.209	1.000	Dihydrogeranylgeranyl diphosphate	$C_{20}H_{38}O_7P_2$		0.302	0.046	1.296
Serum	cold *vs.* normal; hot *vs.* normal; RA *vs.* normal; cold *vs.* hot	776.760	705.581	0.560	SM(d18:0/16:0)	$C_{39}H_{81}N_2O_6P$		0.545	0.164	1.314
Serum	cold *vs.* normal; hot *vs.* normal; RA *vs.* normal; cold *vs.* hot	797.100	718.592	0.550	HexCer-NP t34:0|HexCer-NP t22:0/12:0	$C_{40}H_{79}NO_9$		0.726	0.028	1.898
Serum	cold *vs.* normal; hot *vs.* normal; RA *vs.* normal; cold *vs.* hot	545.830	468.344	1.000	lysoPE 18:0|PE(O-18:0/0:0)	$C_{23}H_{50}NO_6P$		1.816	0.047	1.665
Serum	cold *vs.* normal; hot *vs.* normal; RA *vs.* normal; cold *vs.* hot	600.820	703.537	0.550	PA(14:0/22:1(11Z))	$C_{39}H_{75}O_8P$		1.858	0.007	2.261
Serum	cold *vs.* normal; hot *vs.* normal; RA *vs.* normal; cold *vs.* hot	824.470	732.604	0.550	HexCer-ADS d35:0|HexCer-ADS d18:0/	$C_{41}H_{81}NO_9$		0.677	0.094	1.510
Serum	cold *vs.* normal; hot *vs.* normal; RA *vs.* normal; cold *vs.* hot	744.070	689.558	0.550	PA(P-20:0/16:0)	$C_{39}H_{77}O_7P$		0.870	0.343	1.075
Serum	cold *vs.* normal; hot *vs.* normal; RA *vs.* normal; cold *vs.* hot	637.510	282.278	0.540	Elaidamide	$C_{18}H_{35}NO$	4303-70-2	0.399	0.039	1.858
Serum	cold *vs.* normal	682.100	519.403	0.540	Ganoderiol C	$C_{32}H_{54}O_5$	114567-44-	2.107	0.178	1.193
Serum	cold *vs.* normal; hot *vs.* normal; cold *vs.* hot; RA *vs.* normal	604.790	678.469	0.540	SM d31:3|SM d18:2/13:1|SM(d18:2/13:1	$C_{36}H_{69}N_2O_6P$		1.536	0.090	1.484
Serum	cold *vs.* normal; hot *vs.* normal; cold *vs.* hot; RA *vs.* normal	534.580	172.132	0.540	Gabapentin (Neurontin)		60142-96-3	0.416	0.178	1.283
Serum	cold *vs.* normal; hot *vs.* normal; cold *vs.* hot; RA *vs.* normal	1100.850	844.737	0.540	TG 50:4	$C_{53}H_{94}O_6$		0.468	0.289	1.101
Serum	hot *vs.* normal	504.900	466.327	0.540	PE(O-18:1(9Z)/0:0)	$C_{23}H_{48}NO_6P$		1.372	0.137	1.202
Serum	hot *vs.* normal; RA *vs.* normal	612.100	339.288	0.540	Glycidyl oleate	$C_{21}H_{38}O_3$	5431-33-4	0.594	0.063	1.344
Serum	hot *vs.* normal	203.030	481.261	1.000	Alangicine	$C_{28}H_{36}N_2O_5$	16531-04-7	0.379	0.080	1.277
Serum	cold *vs.* normal; hot *vs.* normal; RA *vs.* normal	734.490	815.698	0.530	SM d42:1|SM d14:1/28:0|SM(d14:1/28:0	$C_{47}H_{95}N_2O_6P$		0.350	0.020	1.900
Serum	cold *vs.* normal; hot *vs.* normal; RA *vs.* normal	770.940	703.574	0.530	SM(d18:0/16:1(9Z))	$C_{39}H_{79}N_2O_6P$		0.790	0.096	1.068
Serum	cold *vs.* normal; hot *vs.* normal; RA *vs.* normal	142.200	211.131	0.530	Cymather aldehyde methyl ester			1.337	0.063	1.171
Serum	cold *vs.* normal	621.790	465.355	0.530	Castasterone	$C_{28}H_{48}O_5$	80736-41-0	1.989	0.004	2.288
Serum	cold *vs.* normal; hot *vs.* normal; RA *vs.* normal	695.860	801.683	0.530	SM d41:1|SM d21:0/20:1|SM(d21:0/20:1	$C_{46}H_{93}N_2O_6P$		0.317	0.192	1.243
Serum	cold *vs.* normal; hot *vs.* normal; RA *vs.* normal	433.200	379.313	0.530	5β-Cholane-3α;,6α;,24-triol	$C_{24}H_{42}O_3$		1.279	0.077	1.138
Serum	cold *vs.* normal; hot *vs.* normal; RA *vs.* normal	846.720	774.541	0.530	PE(22:6(4Z,7Z,10Z,13Z,16Z,19Z)/P-18:	$C_{45}H_{76}NO_7P$		0.505	0.069	1.583
Serum	hot *vs.* normal	90.360	348.079	0.530	2-Hydroxyzotepine	$C_{18}H_{18}ClNO_2S$	78105-26-7	1.355	0.303	1.106
Serum	cold *vs.* normal	818.700	722.510	0.520	plasmenyl-PE 36:5	$C_{41}H_{72}NO_7P$		0.431	0.254	1.056
Serum	cold *vs.* normal	824.190	731.604	0.520	Ubiquinol|QH2|CoQH2	$C_{14}H_{20}O_4(C_5H_8)n$	56275-39-9	0.707	0.268	1.085
Serum	cold *vs.* hot	652.770	563.426	0.520	SM 24:1	$C_{29}H_{59}N_2O_6P$		1.613	0.165	1.344
Serum	cold *vs.* normal; RA *vs.* normal	601.000	704.540	0.520	MGDG 29:1	$C_{38}H_{70}O_{10}$		1.530	0.036	1.738

Serum	hot *vs.* normal	668.750	522.391	0.520	CerP 27:0	$C_{27}H_{56}NO_6P$		0.755	0.149	1.029
Serum	cold *vs.* hot; cold *vs.* normal	836.030	748.526	0.520	plasmenyl-PE 38:6	$C_{43}H_{74}NO_7P$		0.708	0.227	1.169
Serum	cold *vs.* normal	730.580	702.562	0.510	TAG 40:5|TAG 12:3-13:1-15:1|TG 40:5|	$C_{43}H_{72}O_6$		0.904	0.208	1.145
Serum	cold *vs.* hot	748.040	702.561	0.510	HexCer-AS d33:1|HexCer-AS d14:1/19:(	$C_{39}H_{75}NO_9$		1.852	0.175	1.270
Serum	cold *vs.* normal; hot *vs.* normal; RA *vs.* normal	861.160	429.371	0.510	Mangiferdesmethylursanone	$C_{29}H_{48}O_2$	259144-62-	0.651	0.153	1.274
Serum	cold *vs.* normal; hot *vs.* normal; RA *vs.* normal	744.300	345.283	0.510	23-hydroxy-24,25,26,27-tetranorvitamin	$C_{23}H_{36}O_2$		0.763	0.209	1.442
Serum	cold *vs.* hot	874.510	797.651	0.510	SM 39:0	$C_{44}H_{91}N_2O_6P$		1.369	0.236	1.161
Serum	cold *vs.* hot; cold *vs.* normal	708.000	503.408	0.510	(20S)-1α;,25-dihydroxy-20-methoxy-26,2	$C_{32}H_{54}O_4$		1.502	0.239	1.135
Serum	RA *vs.* normal	612.820	801.683	0.500	SM d41:1|SM d14:0/27:1|SM(d14:0/27:1	$C_{46}H_{93}N_2O_6P$		0.569	0.063	1.164
Serum	cold *vs.* normal; RA *vs.* normal	568.820	813.683	0.500	SM d42:2|SM d27:1/15:1|SM(d27:1/15:1	$C_{47}H_{93}N_2O_6P$		0.408	0.025	1.859
Serum	cold *vs.* hot	397.300	209.117	0.500	α-Asaron|trans-Isoasarone|a-Asarone	$C_{12}H_{16}O_3$	2883-98-9	2.328	0.071	1.612
Serum	hot *vs.* normal; RA *vs.* normal; cold *vs.* hot; cold *vs.* normal	1190.710	898.783	0.500	TAG 54:5|TAG 18:1-18:2-18:2|TG 54:5|	$C_{57}H_{100}O_6$		2.280	0.105	1.076
Serum	hot *vs.* normal; RA *vs.* normal; cold *vs.* hot; cold *vs.* normal	254.650	569.313	1.000	5-Oxoavermectin "1b" aglycone	$C_{33}H_{44}O_8$		2.770	0.305	1.063
Serum	hot *vs.* normal; RA *vs.* normal; cold *vs.* hot; cold *vs.* normal	419.890	573.302	1.000	PI(16:0/0:0)	$C_{25}H_{49}O_{12}P$		1.889	0.162	1.304
Serum	hot *vs.* normal; RA *vs.* normal; cold *vs.* hot; cold *vs.* normal	265.330	613.340	1.000	PI(19:1(9Z)/0:0)	$C_{28}H_{53}O_{12}P$		3.275	0.213	1.226
Serum	hot *vs.* normal; RA *vs.* normal; cold *vs.* hot; cold *vs.* normal	286.550	639.226	1.000	4'-Hydroxy-5,7,2'-trimethoxyflavanone 4'	$C_{30}H_{38}O_{15}$		2.243	0.081	1.531
Serum	hot *vs.* normal; RA *vs.* normal; cold *vs.* hot; cold *vs.* normal	770.390	701.339	1.000	Posaconazole (JAN/USAN/INN)|Noxafil	$C_{37}H_{42}F_2N_8O_4$	171228-49-	1.238	0.232	1.316
Serum	hot *vs.* normal; RA *vs.* normal; cold *vs.* hot; cold *vs.* normal	1327.940	827.698	1.000	SM d43:2|SM d16:1/27:1|SM(d16:1/27:1	$C_{48}H_{95}N_2O_6P$		1.795	0.025	1.922
Serum	cold *vs.* hot	1220.360	853.723	1.000	TG 50:2	$C_{53}H_{98}O_6$		0.505	0.241	1.140
Serum	cold *vs.* hot; cold *vs.* normal; hot *vs.* normal; RA *vs.* normal	809.960	768.552	1.000	PC(22:4(7Z,10Z,13Z,16Z)/13:0)	$C_{43}H_{78}NO_8P$		1.304	0.211	1.188
Serum	cold *vs.* hot; cold *vs.* normal; hot *vs.* normal; RA *vs.* normal	486.880	610.368	1.000	PC(16:0/5:0(COOH))	$C_{29}H_{56}NO_{10}P$		1.979	0.023	1.862

Table S4.2. Significantly differential metabolites of serum and synovial fluid samples from RA-Cold and RA-Hot patients detected by LC/MS

Sample type	Groups	Types	English Name	Molecular Formula	CAS No.	FC	P (T test)	VIP
Synovial fluid	RA-Cold vs. RA-Hot	Steroids	Glycodeoxycholic acid	$C_{26}H_{43}NO_5$	360-65-6	0.446	0.049	1.105
Synovial fluid	RA-Cold vs. RA-Hot	Indoles	Indoxyl sulfate	$C_8H_7NO_4S$	2642-37-7	2.44	0.039	1.152
Synovial fluid	RA-Cold vs. RA-Hot	Amino acids	Phenylacetyl-L-glutamine	$C_{13}H_{16}N_2O_4$	28047-15-6	3.012	0.032	1.191
Synovial fluid	RA-Cold vs. RA-Hot	Others	p-Tolyl Sulfate	$C_7H_8O_4S$	91978-69-7	4.715	0.033	1.188
Synovial fluid	RA-Cold vs. RA-Hot	Sphingolipids	Hexadecasphinganine	$C_{16}H_{35}NO_2$		0.453	0.049	1.103
Synovial fluid	RA-Cold vs. RA-Hot	Phospholipids	PI(12:0/22:2(13Z,16Z))	$C_{43}H_{79}O_{13}P$		2.043	0.02	1.287
Synovial fluid	RA-Cold vs. RA-Hot	Fatty acids	Montecristin	$C_{37}H_{66}O_4$	185336-15-6	4.163	0.022	1.315
Serum	RA-Cold vs. RA-Hot	Others	AFMK	$C_{13}H_{16}N_2O_4$	52450-38-1	3.91	0.002	2.367
Serum	RA-Cold vs. RA-Hot	Fatty acids	Stearamide	$C_{18}H_{37}NO$	124-26-5	0.12	0.008	2.093
Serum	RA-Cold vs. RA-Hot	Sphingolipids	SM 42:2	$C_{47}H_{93}N_2O_6P$		3.114	0.014	1.992
Serum	RA-Cold vs. RA-Hot	Terpenoids	Ganoderiol H	$C_{30}H_{50}O_5$	114612-72-5	2.029	0.049	1.727
Serum	RA-Cold vs. RA-Hot	Fatty acids	β-Linoleic acid	$C_{18}H_{32}O_2$	60-33-3	2.312	0.013	2.007
Serum	RA-Cold vs. RA-Hot	Sphingolipids	SM 40:1	$C_{45}H_{91}N_2O_6P$		1.547	0.04	1.853
Synovial fluid	RA-Cold vs. NC	Phospholipids	PE(18:2(9Z,12Z)/0:0)	$C_{23}H_{44}NO_7P$	85046-18-0	0.462	0.017	1.137
Synovial fluid	RA-Cold vs. NC	Phospholipids	LysoPE 20:3	$C_{25}H_{46}NO_7P$		2.079	0.004	1.323
Synovial fluid	RA-Cold vs. NC	Fatty acids	Cis-9,10-Epoxystearic acid	$C_{18}H_{34}O_3$	2443-39-2	0.312	0.021	1.072
Synovial fluid	RA-Cold vs. NC	Steroids	Pubescenol	$C_{28}H_{42}O_6$	90685-93-1	0.389	0.012	1.208
Synovial fluid	RA-Cold vs. NC	Phospholipids	PE 40:7e	$C_{45}H_{78}NO_7P$		0.403	0.017	1.109
Synovial fluid	RA-Cold vs. NC	Ceramides	HexCer-ADS d37:2	$C_{43}H_{81}NO_9$		0.495	0.021	1.071
Synovial fluid	RA-Cold vs. NC	Ceramides	Cer-BDS d46:9	$C_{46}H_{75}NO_4$		3.051	0.02	1.082
Synovial fluid	RA-Cold vs. NC	Ceramides	HexCer-ADS d35:0; HexCer-ADS	$C_{41}H_{81}NO_9$		0.42	0.026	1.044
Synovial fluid	RA-Cold vs. NC	Ceramides	HexCer-AS d39:4	$C_{45}H_{81}NO_9$		0.342	0.028	1.094
Synovial fluid	RA-Cold vs. NC	Terpenoids	Gibberellic acid	$C_{19}H_{22}O_6$	28281	0.133	0.012	1.106
Synovial fluid	RA-Cold vs. NC	Ceramides	Cer 36:1	$C_{36}H_{71}NO_3$		0.248	0.027	1.034
Synovial fluid	RA-Cold vs. NC	Ceramides	HexCer-AS d37:2; HexCer-AS d21	$C_{43}H_{81}NO_9$		0.289	0.01	1.201
Synovial fluid	RA-Cold vs. NC	Sphingolipids	SM d39:1	$C_{44}H_{89}N_2O_6P$		2.303	0.005	1.238

Synovial fluid	RA-Cold vs. NC	Ceramides	Cer 40:2	$C_{40}H_{77}NO_3$		0.171	0.005	1.239
Synovial fluid	RA-Cold vs. NC	Ceramides	HexCer-NDS d42:1	$C_{48}H_{93}NO_8$		0.44	0.005	1.198
Synovial fluid	RA-Cold vs. NC	Fatty acids	Pinolenic Acid	$C_{18}H_{30}O_2$	16833-54-8	0.431	0.032	1.001
Synovial fluid	RA-Cold vs. NC	Phospholipids	PC(12:0/19:0)	$C_{39}H_{78}NO_8P$		2.013	0.019	1.073
Synovial fluid	RA-Cold vs. NC	Others	α-Tocopherol	$C_{29}H_{50}O_2$	59-02-9|10191-41-0	2.888	0.002	1.454
Serum	RA-Cold vs. NC	Phospholipids	PC 16:0-22:7	$C_{47}H_{80}NO_8P$		2.598	0.017	1.978
Serum	RA-Cold vs. NC	Fatty acids	α-Linolenic acid	$C_{18}H_{30}O_2$	463-40-1	2.219	0.043	1.711
Serum	RA-Cold vs. NC	Phospholipids	PC(22:4(7Z,10Z,13Z,16Z)/0:0)	$C_{30}H_{54}NO_7P$		1.506	0.001	2.38
Serum	RA-Cold vs. NC	Phospholipids	PC 32:1	$C_{40}H_{78}NO_8P$		1.961	0.023	1.861
Serum	RA-Cold vs. NC	Amino acids	D-Phenylalanine	$C_9H_{11}NO_2$	673-06-3	1.445	0.001	2.37
Serum	RA-Cold vs. NC	Indoles	Indoline	C_8H_9N	496-15-1	1.382	0.031	1.778
Serum	RA-Cold vs. NC	Others	Castasterone	$C_{28}H_{48}O_5$	80736-41-0	1.989	0.004	2.288
Synovial fluid	RA-Hot vs. NC	Steroids	Beclomethasone	$C_{22}H_{29}ClO_5$	4419-39-0	0.29	0.004	1.099
Synovial fluid	RA-Hot vs. NC	Ceramides	Cer 42:3	$C_{42}H_{79}NO_3$		0.365	0.007	1.034
Synovial fluid	RA-Hot vs. NC	Indoles	Stovaine	$C_{14}H_{21}NO_2$	644-26-8	0.414	0.003	1.132
Synovial fluid	RA-Hot vs. NC	Others	Chalcomoracin	$C_{39}H_{36}O_9$	76472-89-4	0.126	0.006	1.032
Synovial fluid	RA-Hot vs. NC	Steroids	5α-Androstane-11β,17β-diol	$C_{19}H_{32}O_2$		0.079	0.007	1.034
Synovial fluid	RA-Hot vs. NC	Sphingolipids	SM d28:3	$C_{33}H_{63}N_2O_6P$		0.393	0.004	1.056
Synovial fluid	RA-Hot vs. NC	Steroids	Progesterone			0.388	0.005	1.067
Synovial fluid	RA-Hot vs. NC	Phospholipids	PC(16:0/9:0(CHO))	$C_{33}H_{64}NO_9P$		0.388	0.003	1.105
Synovial fluid	RA-Hot vs. NC	Others	1-Hexadecylamine	$C_{16}H_{35}N$	143-27-1	2.409	0	1.253
Synovial fluid	RA-Hot vs. NC	Phospholipids	PC(P-19:1(12Z)/0:0)	$C_{27}H_{54}NO_6P$		2.916	0.007	1.067
Synovial fluid	RA-Hot vs. NC	Phospholipids	PS(P-16:0/13:0)	$C_{35}H_{68}NO_9P$		0.439	0.004	1.062
Synovial fluid	RA-Hot vs. NC	Ceramides	CerP 29:1	$C_{29}H_{58}NO_6P$		2.835	0.002	1.155
Synovial fluid	RA-Hot vs. NC	Ceramides	Cer-NDS d32:0	$C_{32}H_{65}NO_3$		0.047	0.011	1.136
Synovial fluid	RA-Hot vs. NC	Phospholipids	PA(21:0/20:1(11Z))	$C_{44}H_{85}O_8P$		0.444	0.006	1.043
Serum	RA-Hot vs. NC	Phospholipids	PC(P-16:0/22:6(4Z,7Z,10Z,13Z,16	$C_{46}H_{80}NO_7P$		0.512	0.002	1.8
Serum	RA-Hot vs. NC	Ceramides	PE-Cer(d15:1(4E)/22:0(2OH))	$C_{39}H_{79}N_2O_7P$		0.419	0.035	1.316

Serum	RA-Hot vs. NC	Sphingolipids	C18-OH Sulfatide	$C_{42}H_{81}NO_{12}S$	0.288	0.004	1.663
Serum	RA-Hot vs. NC	Sphingolipids	SM d42:2	$C_{47}H_{93}N_2O_6P$	0.272	0.002	1.795
Serum	RA-Hot vs. NC	Terpenoids	Dihydrogeranylgeranyl diphosphat[illegible]	$C_{20}H_{38}O_7P_2$	0.302	0.046	1.296
Serum	RA-Hot vs. NC	Phospholipids	PI 33:2	$C_{42}H_{77}O_{13}P$	1.23	0.03	1.344
