# Supplementary material for: Integrative network fusion-based multi-omics study for biomarker identification and patient classification of rheumatoid arthritis
Source: Chin Med. 2023 May 4;18:48. doi: 10.1186/s13020-023-00750-8 (PMC10158004; doi:10.1186/s13020-023-00750-8)
Supplement: Supplementary file 5 — Additional file 5. Significantly differential metabolites of urine and serum samples from AIA-Cold and AIA-Hot rats detected by LC/MS: Table S5.1. Significantly differential metabolites of urine samples from AIA-Cold rats detected by LC/MS. Table S5.2. Significantly differential metabolites of serum samples from AIA-Cold rats detected by LC/MS. Table S5.3. Significantly differential metabolites of urine samples from AIA-Hot rats detected by LC/MS. Table S5.4. Significantly differential metabolites of serum samples from AIA-Hot rats detected by LC/MS. [file 13020_2023_750_MOESM5_ESM.pdf]

Table S5.1. Significantly differential metabolites of urine samples from AIA-Cold rats detected by LC/MS

Groups	Samples	Description	AIA-Cold/Con	Log2FC	Pathways
AIA-Cold	Urine	S-(5'-Adenosyl)-L-Methionine	0.493307557	-1.01944071	Arginine and proline metabolism
AIA-Cold	Urine	Creatine	8.366479749	3.064620727	Arginine and proline metabolism
AIA-Cold	Urine	4-Guanidinobutanoate	0.456869248	-1.13014676	Arginine and proline metabolism
AIA-Cold	Urine	Thiamine Pyrophosphate	0.435838836	-1.19813334	Citrate cycle (TCA cycle)
AIA-Cold	Urine	Phosphoenolpyruvate	9.225972078	3.205700926	Citrate cycle (TCA cycle)
AIA-Cold	Urine	Citrate	13.51756784	3.756763692	Citrate cycle (TCA cycle)
AIA-Cold	Urine	UDP-Glucose	0.162301075	-2.62325554	Galactose metabolism
AIA-Cold	Urine	Stachyose	21.13657715	4.401669861	Galactose metabolism
AIA-Cold	Urine	Glucose	2.240857499	1.164050907	Galactose metabolism
AIA-Cold	Urine	D-(+)-Raffinose	18.96958542	4.245616244	Galactose metabolism
AIA-Cold	Urine	2-Deoxy-D-Glucose	2.164046845	1.11373173	Galactose metabolism
AIA-Cold	Urine	Phenylpyruvate	0.46797327	-1.09550197	Phenylalanine metabolism
AIA-Cold	Urine	Phenylacetic Acid	0.389913184	-1.35877516	Phenylalanine metabolism
AIA-Cold	Urine	Phenylacetaldehyde	0.096844459	-3.36818668	Phenylalanine metabolism
AIA-Cold	Urine	Hippurate	0.464735508	-1.10551822	Phenylalanine metabolism
AIA-Cold	Urine	Tetrahydrocortisol	34.98765671	5.128774138	Steroid hormone biosynthesis
AIA-Cold	Urine	Testosterone	2.338566587	1.225624508	Steroid hormone biosynthesis
AIA-Cold	Urine	Progesterone	75.8709099	6.245474935	Steroid hormone biosynthesis
AIA-Cold	Urine	Pregnenolone	0.181938097	-2.45848043	Steroid hormone biosynthesis
AIA-Cold	Urine	Dehydroepiandrosterone sulfate	18.11655182	4.179236484	Steroid hormone biosynthesis
AIA-Cold	Urine	Dehydroepiandrosterone	6.341607405	2.664848566	Steroid hormone biosynthesis
AIA-Cold	Urine	Cortisone	4.262827019	2.091810513	Steroid hormone biosynthesis
AIA-Cold	Urine	Cortisol	5.431122366	2.441250368	Steroid hormone biosynthesis
AIA-Cold	Urine	Corticosterone	10.82678542	3.436533051	Steroid hormone biosynthesis
AIA-Cold	Urine	Androsterone	6.847270516	2.77552901	Steroid hormone biosynthesis
AIA-Cold	Urine	Androstenedione	22.48667876	4.490998688	Steroid hormone biosynthesis
AIA-Cold	Urine	17-hydroxypregnenolone	38.19807245	5.255427934	Steroid hormone biosynthesis
AIA-Cold	Urine	Taurine	10.38314551	3.376171661	Taurine and hypotaurine metabolism
AIA-Cold	Urine	Hypotaurine	0.356084792	-1.48970727	Taurine and hypotaurine metabolism
AIA-Cold	Urine	Kynurenine	0.435052474	-1.20073867	Tryptophan metabolism
AIA-Cold	Urine	Indole-3-Acetic Acid	0.404480557	-1.30585774	Tryptophan metabolism
AIA-Cold	Urine	Anthranilate	0.476646397	-1.0690087	Tryptophan metabolism
AIA-Cold	Urine	5-Hydroxy-L-TryptopHan	2.529955097	1.33911178	Tryptophan metabolism
AIA-Cold	Urine	5-Hydroxyindoleacetate	4.320686708	2.111260625	Tryptophan metabolism
AIA-Cold	Urine	3-Hydroxykynurenine	0.375290127	-1.41392176	Tryptophan metabolism
AIA-Cold	Urine	Xanthine	6.911980056	2.789099055	
AIA-Cold	Urine	Trans-Cinnamate	0.299095254	-1.74132307	
AIA-Cold	Urine	Trans-Cinnamaldehyde	0.356723743	-1.48712085	
AIA-Cold	Urine	Threonine	9.556989015	3.25655616	
AIA-Cold	Urine	Sorbate	15.04482367	3.911195293	
AIA-Cold	Urine	Sn-Glycero-3-Phosphocholine	30.37905837	4.925005247	
AIA-Cold	Urine	Salicylic Acid	0.449927047	-1.152237	
AIA-Cold	Urine	Retinol	0.307094825	-1.70324389	
AIA-Cold	Urine	Quinoline	0.389852028	-1.35900146	
AIA-Cold	Urine	Pantolactone	21.79111555	4.445668149	
AIA-Cold	Urine	Oxalomalic Acid	0.361024983	-1.46982942	
AIA-Cold	Urine	Nonanoate	0.391239189	-1.35387721	
AIA-Cold	Urine	N-Methyl-L-Glutamate	10.11304379	3.338145374	
AIA-Cold	Urine	Nalpha-Acetyl-L-Lysine	2.276706577	1.186948368	
AIA-Cold	Urine	N-Acetylneuraminate	2.227813456	1.155628435	
AIA-Cold	Urine	N-Acetyl-L-Cysteine	0.378552122	-1.40143614	
AIA-Cold	Urine	N,N-Dimethyl-1,4-Phenylenediamine	0.408605053	-1.29122105	
AIA-Cold	Urine	Methyl Jasmonate	5.420272604	2.438365412	
AIA-Cold	Urine	L-Homocysteine Thiolactone	0.442256571	-1.17704452	
AIA-Cold	Urine	Leukotriene B4	5.381696155	2.42806094	
AIA-Cold	Urine	Inosine	6.825231904	2.770878066	Purine metabolism
AIA-Cold	Urine	Hydroxypyruvate	217.9797829	7.768050525	
AIA-Cold	Urine	Hydroxyproline	0.437067861	-1.1940708	

AIA-Cold	Urine	Hexadecanoic Acid	2.601195915	1.379175063	
AIA-Cold	Urine	Glyceraldehyde	2.452813066	1.294437287	
AIA-Cold	Urine	Glutarate	3.54718143	1.826673123	
AIA-Cold	Urine	Gluconic Acid	3.202269783	1.679094856	
AIA-Cold	Urine	Geranylgeranyl Pyrophosphate	3.035373634	1.601874113	
AIA-Cold	Urine	Flumethasone	0.042990782	-4.53982885	
AIA-Cold	Urine	Farnesyl Diphosphate	2.090732878	1.064008748	
AIA-Cold	Urine	Ethyl 3-Ureidopropionate	22.86375791	4.514990641	
AIA-Cold	Urine	Epinephrine	0.46719575	-1.09790094	
AIA-Cold	Urine	D-Ribulose 1,5-Bisphosphate	0.307929047	-1.69933013	
AIA-Cold	Urine	D-Lactose	4.555747916	2.18768792	
AIA-Cold	Urine	Dethiobiotin	7.212633035	2.850526024	
AIA-Cold	Urine	Deoxyribose	2.47460175	1.307196363	
AIA-Cold	Urine	D-(+)-Galacturonic Acid	2.024439751	1.017522708	
AIA-Cold	Urine	Cytidine 5'-Diphosphate	0.388450448	-1.36419752	
AIA-Cold	Urine	Citramalate	2.764924578	1.467240127	
AIA-Cold	Urine	Cholic Acid	5.414686778	2.436877885	
AIA-Cold	Urine	cGMP	4.540612856	2.182887034	
AIA-Cold	Urine	Carnitine	21.79275615	4.445776762	
AIA-Cold	Urine	Carbamoylphosphate	9.977406658	3.318664877	
AIA-Cold	Urine	C22-OH Sulfatide	33.83084174	5.080267168	
AIA-Cold	Urine	Benzyl Alcohol	0.481707231	-1.05377151	
AIA-Cold	Urine	Beclomethasone	25.37518169	4.665346247	
AIA-Cold	Urine	Azelaic Acid	3.027059042	1.597916815	
AIA-Cold	Urine	Aniline-2-Sulfonic Acid	0.496447511	-1.0102869	
AIA-Cold	Urine	Aniline	0.271501748	-1.88096661	
AIA-Cold	Urine	AMP	2.920232314	1.546083145	
AIA-Cold	Urine	6-Phosphogluconate	3.761257247	1.911214981	
AIA-Cold	Urine	5,6-Dihydrouracil	4.195313606	2.068778658	
AIA-Cold	Urine	4-Hydroxybenzaldehyde	0.324205044	-1.62502156	
AIA-Cold	Urine	3-Methoxy-L-Tyrosine	4.511241417	2.173524493	
AIA-Cold	Urine	3-Hydroxybutanoic Acid	2.083795397	1.05921363	
AIA-Cold	Urine	2'-Deoxyguanosine	0.430655361	-1.21539431	Purine metabolism
AIA-Cold	Urine	2'-Deoxyadenosine	2.729267466	1.448513784	Purine metabolism
AIA-Cold	Urine	2,6-Dihydroxypyridine	0.491053548	-1.02604774	
AIA-Cold	Urine	1-Methylhistidine	2.835295542	1.503499125	
AIA-Cold	Urine	(S)-1-PhenyletHanol	0.420250544	-1.25067841	

Table S5.2. Significantly differential metabolites of serum samples from AIA-Cold rats detected by LC/MS

Groups	Samples	Description	AIA-Cold/Con	Log2FC	Pathways
AIA-Cold	Serum	Phenylacetaldehyde	0.085664951	-3.54515113	Phenylalanine metabolism
AIA-Cold	Serum	Dehydroepiandrosterone sulfate	1.501265023	0.586178683	Steroid hormone biosynthesis
AIA-Cold	Serum	Androsterone	0.059615889	-4.0681593	Steroid hormone biosynthesis
AIA-Cold	Serum	Pregnenolone	0.037170839	-4.74968494	Steroid hormone biosynthesis
AIA-Cold	Serum	Dehydroepiandrosterone	0.243032474	-2.04077899	Steroid hormone biosynthesis
AIA-Cold	Serum	17-hydroxypregnenolone	0.087797258	-3.5096803	Steroid hormone biosynthesis
AIA-Cold	Serum	Tetrahydrodeoxycorticosterone	0.008080726	-6.95129933	Steroid hormone biosynthesis
AIA-Cold	Serum	Pregnanolone	0.136743994	-2.87045063	Steroid hormone biosynthesis
AIA-Cold	Serum	Menaquinone	0.2327841	-2.10293558	Ubiquinone biosynthesis
AIA-Cold	Serum	Phylloquinone	0.008918786	-6.80893686	Ubiquinone biosynthesis
AIA-Cold	Serum	2-Arachidonoylglycerol	0.034884515	-4.84126941	
AIA-Cold	Serum	Farnesyl Diphosphate	2.456275287	1.29647226	
AIA-Cold	Serum	Amylose	0.191652544	-2.38343494	
AIA-Cold	Serum	Caffeine	0.397726454	-1.33015157	
AIA-Cold	Serum	Uridine 5'-Diphosphoglucuronic Acid	0.041484101	-4.59129768	
AIA-Cold	Serum	Myristic Acid	0.528758247	-0.91931984	
AIA-Cold	Serum	N,N-Dimethyl-1,4-Phenylenediamine	0.107479449	-3.21786726	
AIA-Cold	Serum	Retinol	0.101298017	-3.30332216	
AIA-Cold	Serum	9E-Octadecenoic Acid	0.163647288	-2.6113384	
AIA-Cold	Serum	Glyoxylic Acid	0.127665057	-2.96956439	
AIA-Cold	Serum	PCe 36:5	4.213406309	2.074987045	
AIA-Cold	Serum	N-Acetyl-Dl-Glutamic Acid	0.084692242	-3.56162637	
AIA-Cold	Serum	LPC 20:1	2.569771867	1.361640289	
AIA-Cold	Serum	LPC 20:2	0.189925193	-2.39649681	
AIA-Cold	Serum	PC 38:7	2.986995377	1.578695005	
AIA-Cold	Serum	Rac-Glycerol 1-Myristate	3.239348281	1.695703589	
AIA-Cold	Serum	Octadecanoic Acid	2.09543978	1.067253061	
AIA-Cold	Serum	1-Oleoyl-Rac-Glycerol	3.012716657	1.591064995	
AIA-Cold	Serum	Desmosterol	0.023735747	-5.39679475	
AIA-Cold	Serum	C18DH Cer	0.457969643	-1.12667612	
AIA-Cold	Serum	PG 38:3	3.593510032	1.845393713	
AIA-Cold	Serum	PS 28:2	0.0607965	-4.03986792	
AIA-Cold	Serum	Dihydroorotic Acid	0.38068243	-1.39334011	
AIA-Cold	Serum	C12 Sphingomyelin	2.135159006	1.094343512	
AIA-Cold	Serum	Beclomethasone	0.005371525	-7.54045257	
AIA-Cold	Serum	Geranyl Pyrophosphate	2.322803574	1.215867159	
AIA-Cold	Serum	PEp 36:4	2.741021464	1.454713626	
AIA-Cold	Serum	Citramalate	2.239983112	1.163487855	
AIA-Cold	Serum	L-Anserine	3.268028875	1.70842073	
AIA-Cold	Serum	C22DH Sphingomyelin	3.665688221	1.874084085	
AIA-Cold	Serum	MG (16:0/0:0/0:0)	2.151450087	1.10530937	
AIA-Cold	Serum	Quinate	0.2133233	-2.22888655	
AIA-Cold	Serum	Benzylamine	4.767364724	2.253192002	
AIA-Cold	Serum	Leukotriene B4	2.059564909	1.042339595	
AIA-Cold	Serum	PS 28:0	2.794710358	1.482698771	
AIA-Cold	Serum	Glycocholic Acid	0.356330801	-1.4887109	
AIA-Cold	Serum	LPC 18:0	0.12313413	-3.02169739	
AIA-Cold	Serum	Cholic Acid	6.584722518	2.719122646	
AIA-Cold	Serum	HexCer d18:1/16:0	0.080578544	-3.63346045	
AIA-Cold	Serum	Taurocholic Acid	21.06141075	4.39653017	

Table S5.3. Significantly differential metabolites of urine samples from AIA-Hot rats detected by LC/MS

Groups	Samples	Description	AIA-Hot/Con	Log2FC	Pathways
AIA-Hot	Urine	4-Aminobutanoate	2.044823916	1.031976615	Arginine and proline metabolism
AIA-Hot	Urine	Creatine	0.143997809	-2.79588124	Arginine and proline metabolism
AIA-Hot	Urine	4-Guanidinobutanoate	23.41724783	4.549499624	Arginine and proline metabolism
AIA-Hot	Urine	Phosphocreatine	2.90095955	1.536530179	Arginine and proline metabolism
AIA-Hot	Urine	S-(5'-Adenosyl)-L-Methionine	2.337731493	1.225109235	Arginine and proline metabolism
AIA-Hot	Urine	Glutamine	0.37495249	-1.41522029	D-Glutamine and D-glutamate metabolism/Purine metabolism/Nitrogen metabolism
AIA-Hot	Urine	Glutamic Acid	0.064917039	-3.94525899	D-Glutamine and D-glutamate metabolism/Arginine and proline metabolism/Nitrogen metabolism
AIA-Hot	Urine	Raffinose	0.314995055	-1.66659891	Galactose metabolism
AIA-Hot	Urine	Phenylacetaldehyde	24.91582189	4.638990259	Phenylalanine metabolism
AIA-Hot	Urine	Phenylacetic Acid	2.569597662	1.361542485	Phenylalanine metabolism
AIA-Hot	Urine	Phenylpyruvate	2.442241517	1.288205878	Phenylalanine metabolism
AIA-Hot	Urine	Hippurate	2.253015847	1.171857461	Phenylalanine metabolism
AIA-Hot	Urine	Hypoxanthine	0.46879196	-1.09298027	Purine metabolism
AIA-Hot	Urine	L-serine	0.06227038	-4.00531012	Sphingolipid metabolism
AIA-Hot	Urine	Sphinganine	8.302555194	3.053555409	Sphingolipid metabolism
AIA-Hot	Urine	Ceramide (d18:1/12:0)	2.44660438	1.290780834	Sphingolipid metabolism
AIA-Hot	Urine	Androstenedione	0.035752109	-4.80582784	Steroid hormone biosynthesis
AIA-Hot	Urine	Dehydroepiandrosterone	0.196578382	-2.34682342	Steroid hormone biosynthesis
AIA-Hot	Urine	Androsterone	0.128933709	-2.9552986	Steroid hormone biosynthesis
AIA-Hot	Urine	Progesterone	0.199561593	-2.325094	Steroid hormone biosynthesis
AIA-Hot	Urine	Dihydrotestosterone	0.19921094	-2.32763122	Steroid hormone biosynthesis
AIA-Hot	Urine	Pregnanolone	2.296139891	1.19921054	Steroid hormone biosynthesis
AIA-Hot	Urine	Pregnenolone	8.051131646	3.009191579	Steroid hormone biosynthesis
AIA-Hot	Urine	Cortisol	2.114742703	1.080482144	Steroid hormone biosynthesis
AIA-Hot	Urine	Corticosterone	0.189808554	-2.39738308	Steroid hormone biosynthesis
AIA-Hot	Urine	Tetrahydrocortisol	0.331638757	-1.59231548	Steroid hormone biosynthesis
AIA-Hot	Urine	Hypotaurine	2.639179228	1.400089328	Taurine and hypotaurine metabolism
AIA-Hot	Urine	Taurine	0.047043756	-4.40985294	Taurine and hypotaurine metabolism
AIA-Hot	Urine	Hydroquinone	2.673847718	1.418917303	
AIA-Hot	Urine	Aniline	5.60905543	2.48775784	
AIA-Hot	Urine	Succinic Acid	0.113926009	-3.13383095	
AIA-Hot	Urine	Threonine	0.056767252	-4.13879728	
AIA-Hot	Urine	Nicotinic acid	8.133289002	3.023838878	
AIA-Hot	Urine	4-Methylcatechol	9.910368025	3.308938633	
AIA-Hot	Urine	Benzyl Alcohol	6.3729099	2.671952264	
AIA-Hot	Urine	5,6-Dihydrouracil	0.305430436	-1.71108426	
AIA-Hot	Urine	Isoleucine	3.916838119	1.969689504	
AIA-Hot	Urine	Glutarate	4.446894019	2.152798022	
AIA-Hot	Urine	N,N-Dimethyl-1,4-Phenylenediamine	2.257826228	1.174934455	
AIA-Hot	Urine	5-Valerolactone	4.237056135	2.083062243	
AIA-Hot	Urine	Nonanoate	2.734816338	1.451443949	
AIA-Hot	Urine	L-Homocysteine Thiolactone	4.129844498	2.04608746	
AIA-Hot	Urine	4-Hydroxybenzaldehyde	3.19516483	1.675890358	
AIA-Hot	Urine	Mono-Methyl Glutarate	0.20149746	-2.31116644	
AIA-Hot	Urine	3-Methyl-2-Oxindole	2.762815524	1.466139235	
AIA-Hot	Urine	Heptanoic Acid	4.004637826	2.001671773	
AIA-Hot	Urine	Trans-Cinnamaldehyde	3.009911704	1.589721166	
AIA-Hot	Urine	Suberic Acid	5.668115903	2.502869259	
AIA-Hot	Urine	6-Carboxyhexanoate	0.173246851	-2.52909896	
AIA-Hot	Urine	D-(+)-Glucosamine	0.291727392	-1.77730724	
AIA-Hot	Urine	N-Acetyl-L-Cysteine	16.18121242	4.016247805	
AIA-Hot	Urine	4-Hydroxybenzaldehyde	9.813788521	3.294810183	
AIA-Hot	Urine	Meso-Tartaric Acid	2.138434115	1.096554758	
AIA-Hot	Urine	Pantolactone	0.076860272	-3.70161812	

AIA-Hot	Urine	Indole-3-Acetic Acid	2.042865901	1.030594505	
AIA-Hot	Urine	Carnitine	3.205074038	1.680357684	
AIA-Hot	Urine	Phosphorylcholine	2.427076115	1.279219353	
AIA-Hot	Urine	Azelaic Acid	0.277582461	-1.84901168	
AIA-Hot	Urine	1-Hydroxy-2-Naphthoate	4.511253056	2.173528215	
AIA-Hot	Urine	Glucuronic Acid	0.480368567	-1.05778634	
AIA-Hot	Urine	Ascorbate	0.48401382	-1.04687985	
AIA-Hot	Urine	D-Gulonic Acid Gama-Lactone	0.37980118	-1.39668371	
AIA-Hot	Urine	1-Methyl-6,7-Dihydroxy-1,2,3,4-Tetra	2.95857316	1.564901571	
AIA-Hot	Urine	2-Acetamido-2-Deoxy-Beta-D-Glucos	0.332403636	-1.58899193	
AIA-Hot	Urine	Indole-3-Acetaldehyde	2.087995174	1.062118377	
AIA-Hot	Urine	Methyl Indole-3-Acetate	2.013632644	1.00980051	
AIA-Hot	Urine	N-Acetyl-L-Phenylalanine	0.120533272	-3.05249666	
AIA-Hot	Urine	3-Dehydroshikimate	0.155273296	-2.68711836	
AIA-Hot	Urine	3-Methoxytyrosine	0.184629026	-2.43729871	
AIA-Hot	Urine	Gluconic Acid	2.065657947	1.046601378	
AIA-Hot	Urine	Indole-3-Acetamide	0.160798996	-2.63666969	
AIA-Hot	Urine	D-Pantothenic Acid	0.016419503	-5.92844577	
AIA-Hot	Urine	Acetylglucosamine	2.338322402	1.225473859	
AIA-Hot	Urine	Lipoamide	5.346355192	2.418555688	
AIA-Hot	Urine	S-Carboxymethyl-L-Cysteine	0.365937692	-1.45033007	
AIA-Hot	Urine	Nalpha-Acetyl-L-Lysine	2.18721332	1.129093934	
AIA-Hot	Urine	Ferulate	0.01262304	-6.30779681	
AIA-Hot	Urine	Sn-Glycero-3-Phosphocholine	0.262045838	-1.9321089	
AIA-Hot	Urine	2'-Deoxyguanosine	0.012572563	-6.31357739	Purine metabolism
AIA-Hot	Urine	Adenosine	0.012572563	-6.31357739	Purine metabolism
AIA-Hot	Urine	Deoxyuridine	2.523399475	1.335368615	
AIA-Hot	Urine	5'-Deoxyadenosine	0.326125918	-1.61649899	
AIA-Hot	Urine	10E-Heptadecenoic Acid	0.084533656	-3.56433034	Purine metabolism
AIA-Hot	Urine	Nandrolone	2.954798741	1.563059868	
AIA-Hot	Urine	Retinol	2.367473237	1.243348117	
AIA-Hot	Urine	Guanosine	0.149516904	-2.74161949	Purine metabolism
AIA-Hot	Urine	N-Acetylneuraminate	0.398791154	-1.32629469	
AIA-Hot	Urine	Inosine	0.023228621	-5.42795266	Purine metabolism
AIA-Hot	Urine	11Z-Eicosenoic Acid	0.376802829	-1.4081183	
AIA-Hot	Urine	cAMP	0.300084028	-1.73656156	
AIA-Hot	Urine	Leukotriene B4	0.154041837	-2.69860586	
AIA-Hot	Urine	2'-Deoxyadenosine 5'-Diphosphate	0.29679473	-1.75246262	Purine metabolism
AIA-Hot	Urine	Geranylgeranyl Pyrophosphate	0.21744592	-2.20127146	
AIA-Hot	Urine	PE(O-18:0/0:0)	4.995015709	2.320489215	
AIA-Hot	Urine	Dihydrofolate	0.212742014	-2.23282312	
AIA-Hot	Urine	Taurodeoxycholic Acid	3.700603069	1.887760399	
AIA-Hot	Urine	LysoPC(18:0/0:0)	4.756729071	2.249969856	
AIA-Hot	Urine	Protoporphyrin	0.060312046	-4.05141002	
AIA-Hot	Urine	Nicotinic Acid Adenine Dinucleotide	3.467244377	1.793789524	
AIA-Hot	Urine	SM(d18:1/18:0)	0.371428486	-1.42884363	

Table S5.4. Significantly differential metabolites of serum samples from AIA-Hot rats detected by LC/MS

Groups	Samples	Description	AIA-Hot/Con	Log2FC	Pathways
AIA-Hot	Serum	S-(5'-Adenosyl)-L-Methionine	0.341507605	-1.55001039	Arginine and proline metabolism
AIA-Hot	Serum	Citrate	0.091899588	-3.44379779	Citrate cycle (TCA cycle)
AIA-Hot	Serum	Glutamine	0.036014934	-4.79526094	D-Glutamine and D-glutamate metabolism
AIA-Hot	Serum	N-Acetyl-Dl-Glutamic Acid	0.085949572	-3.54036573	D-Glutamine and D-glutamate metabolism
AIA-Hot	Serum	Phenylacetaldehyde	0.060179822	-4.05457635	Phenylalanine metabolism
AIA-Hot	Serum	3-O-Sulfogalactosylceramide (d18:1/2	0.335489238	-1.57566161	Sphingolipid metabolism
AIA-Hot	Serum	Galactosylceramide (d18:1/16:0)	2.247247372	1.168158942	Sphingolipid metabolism
AIA-Hot	Serum	Androsterone	0.025904467	-5.2706553	Steroid hormone biosynthesis
AIA-Hot	Serum	Dehydroepiandrosterone	0.16542747	-2.59572927	Steroid hormone biosynthesis
AIA-Hot	Serum	Tetrahydrodeoxycorticosterone	0.450009036	-1.15197413	Steroid hormone biosynthesis
AIA-Hot	Serum	Pregnenolone	0.051423386	-4.28143158	Steroid hormone biosynthesis
AIA-Hot	Serum	Tetrahydrodeoxycortisol	0.3733819	-1.4212761	Steroid hormone biosynthesis
AIA-Hot	Serum	17-hydroxypregnenolone	0.009701071	-6.68764028	Steroid hormone biosynthesis
AIA-Hot	Serum	Dehydroepiandrosterone sulfate	0.028546136	-5.13056069	Steroid hormone biosynthesis
AIA-Hot	Serum	Cortisol	68.74960866	6.103279596	Steroid hormone biosynthesis
AIA-Hot	Serum	Menaquinone	0.012833939	-6.28389215	Ubiquinone biosynthesis
AIA-Hot	Serum	Phylloquinone	0.012826729	-6.28470293	Ubiquinone biosynthesis
AIA-Hot	Serum	3-Methyl-2-Oxovaleric Acid	0.246900986	-2.0179955	
AIA-Hot	Serum	Hexanoate	0.082109664	-3.60630416	
AIA-Hot	Serum	Proline	0.355276248	-1.49298686	
AIA-Hot	Serum	S-Carboxymethyl-L-Cysteine	0.486953058	-1.03814539	
AIA-Hot	Serum	N,N-Dimethyl-1,4-Phenylenediamine	0.194795837	-2.35996525	
AIA-Hot	Serum	Pyridines-2,3-Dicarboxylate	0.1633833	-2.61366756	
AIA-Hot	Serum	L-Anserine	6.718563555	2.748152814	
AIA-Hot	Serum	Uridine	0.334721973	-1.57896483	
AIA-Hot	Serum	Myristic Acid	0.467558479	-1.09678128	
AIA-Hot	Serum	Quinate	0.332870835	-1.58696562	
AIA-Hot	Serum	Caffeine	0.202977456	-2.30060859	
AIA-Hot	Serum	O-Succinyl-L-Homoserine	0.009647507	-6.6956281	
AIA-Hot	Serum	9Z-Hexadecenoic Acid	0.039263951	-4.67065085	
AIA-Hot	Serum	9E-Octadecenoic Acid	0.079611808	-3.65087377	
AIA-Hot	Serum	Retinol	0.031845181	-4.97278112	
AIA-Hot	Serum	Geranyl Pyrophosphate	0.395528879	-1.33814506	
AIA-Hot	Serum	Rac-Glycerol 1-Myristate	0.061723765	-4.01803013	
AIA-Hot	Serum	MG (16:0/0:0/0:0)	0.063881237	-3.96846394	
AIA-Hot	Serum	D-(+)-Cellobiose	0.153716799	-2.70165326	
AIA-Hot	Serum	Amylose	0.067385014	-3.89142842	
AIA-Hot	Serum	Farnesyl Diphosphate	0.040490844	-4.62626046	
AIA-Hot	Serum	Desmosterol	0.008078883	-6.95162853	
AIA-Hot	Serum	Beclomethasone	0.24175769	-2.04836631	
AIA-Hot	Serum	2-Arachidonoylglycerol	0.00212198	-8.88037343	
AIA-Hot	Serum	Cholic Acid	0.223824238	-2.15956182	
AIA-Hot	Serum	LPE 16:1	0.347283361	-1.5258148	
AIA-Hot	Serum	LPC 18:0	0.289924559	-1.78625055	
AIA-Hot	Serum	Taurocholic Acid	0.164253952	-2.60600001	
AIA-Hot	Serum	Protoporphyrin	0.247168729	-2.01643186	
AIA-Hot	Serum	Uridine 5'-Diphosphoglucuronic Acid	0.036345867	-4.78206488	
AIA-Hot	Serum	PS 28:2	0.001490531	-9.3899578	
AIA-Hot	Serum	SM(d18:1/16:0)	0.321068041	-1.63904903	
AIA-Hot	Serum	PCe 34:1	0.120705248	-3.0504397	
AIA-Hot	Serum	PEp 36:1	0.45073195	-1.14965838	
AIA-Hot	Serum	PC 34:2	0.305172607	-1.71230263	
AIA-Hot	Serum	PE 38:6-A	0.364154395	-1.45737784	
AIA-Hot	Serum	SM(d18:1/18:0)	0.34730691	-1.52571698	
AIA-Hot	Serum	PC 36:1	0.244286036	-2.0333567	
AIA-Hot	Serum	PCe 36:4	0.306954399	-1.70390375	
AIA-Hot	Serum	PC 38:0 (19:0/19:0)	4.880924848	2.287154538	
AIA-Hot	Serum	FAD	0.417088056	-1.2615761	
AIA-Hot	Serum	C22DH Sphingomyelin	0.186360537	-2.42383171	
AIA-Hot	Serum	PCe 36:3	2.831834888	1.50173715	
AIA-Hot	Serum	PC 38:7	0.358681796	-1.47922357	
AIA-Hot	Serum	PG 38:3	0.41592409	-1.26560785	
AIA-Hot	Serum	PC 40:6	0.386014985	-1.37327124	
